# Exploiting the 2‑(1,3,4,9-tetrahydropyrano[3,4‑*b*]indol-1-yl)acetic Acid Scaffold to Generate COXTRANs: A New Class of Dual Cyclooxygenase Inhibitors-Thromboxane Receptor Antagonists

**DOI:** 10.1021/acs.jmedchem.5c02068

**Published:** 2025-10-22

**Authors:** Federica Blua, Francesca Boccato, Carola Buccellati, Patrizia Risè, Silvia Barbieri, Laura Castiglioni, Annika Balzulat, Barbara Rolando, Elisabetta Marini, Marta Balestra, Maria Luisa Introvigne, Marta Giorgis, Luigi Sironi, Stefania Tacconelli, Kerstin Hiesinger, Paola Patrignani, Achim Schmidtko, Dieter Steinhilber, Ewgenij Proschak, Angelo Sala, G. Enrico Rovati, Massimo Bertinaria

**Affiliations:** † Department of Drug Science and Technology, 9314 University of Turin, Via P. Giuria 9, Turin 10125, Italy; ‡ Institute of Pharmaceutical Chemistry, 9173 Goethe-University, Max-von-Laue-Str. 9, Frankfurt am Main D-60438, Germany; § Department of Neuroscience, Imaging and Clinical Sciences, “G. d’Annunzio” University, Chieti 66100, Italy; ∥ Department of Pharmaceutical Sciences, 9304 University of Milan, Milan 20122, Italy; ⊥ Laboratory of Systems Pharmacology and Translational Therapies, Center for Advanced Studies and Technology (CAST), ″G. d’Annunzio″ University, Chieti 66100, Italy; # Institute of Pharmacology and Clinical Pharmacy, Goethe University, Max-von-Laue-Str. 9, Frankfurt am Main D-60438, Germany; ¶ 18607 Centro Cardiologico Monzino IRCCS, Milan 20138, Italy

## Abstract

The use of traditional nonsteroidal anti-inflammatory drugs (NSAIDs) and coxibs is effective for the treatment of inflammatory pain and chronic inflammatory conditions. However, their use is associated with enhanced risk of cardiovascular toxicity and thrombotic events, particularly for the latter. The vascular side effects of these drugs could be mitigated by pharmacological inhibition of the thromboxane A_2_ receptor (TP). Here we describe the development of a new class of dual cyclooxygenase (COX) inhibitors/thromboxane receptor antagonists (COXTRANs) based on the 2-(1,3,4,9-tetrahydropyrano­[3,4-*b*]­indol-1-yl)­acetic acid scaffold. The in vitro evaluation of 50 newly synthesized compounds resulted in a set of well-balanced compounds exhibiting nanomolar activity on both COX-2 and TP receptor. Further studies in human whole blood and physicochemical profiling allowed the prioritization of **51** (CXT29) as a suitable candidate for in vivo studies. Compound **51**, after oral administration, was able to prevent TP receptor-mediated platelet aggregation and to reduce inflammatory pain in mice.

## Graphical abstract

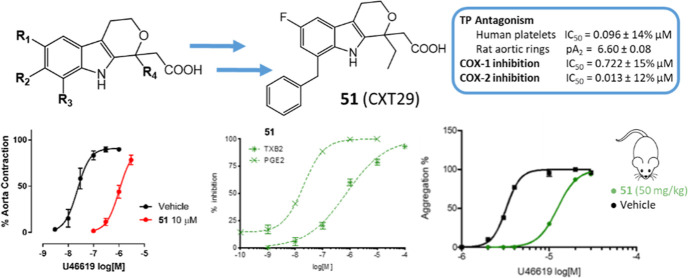

## Introduction

Chronic inflammation is a major global health issue that affects millions of individuals worldwide. Inflammation is a part of the innate response to offending stimuli, and it is necessary for maintaining the body’s integrity. However, unresolved tissue inflammation leads to chronic inflammation, which is associated with the development of various pathological conditions.^1–3^ According to a recent GBD (Global Burden of Disease) report, chronic inflammatory diseases have been recognized as the most significant cause of death, with more than 50% of all deaths being attributable to inflammation-related disorders such as ischemic heart disease, stroke, cancer, diabetes, nonalcoholic fatty liver disease, and autoimmune and neurodegenerative conditions.^4^ The management of chronic inflammation associated with various chronic diseases is a complex task, as it is often complicated by the lack of knowledge about the precise identity of the inflammatory stimulus. Different pharmacological approaches can be used to target various signaling pathways.^5^ Nonsteroidal anti-inflammatory drugs (NSAIDs) are among the most frequently prescribed and consumed drugs and represent the first and most common option for the symptomatic treatment of mild to moderate inflammatory pain^6,7^ and the management of chronic inflammatory diseases such as osteoarthritis,^8,9^ rheumatoid arthritis, and musculoskeletal injuries.^10,11^ NSAIDs exert their anti-inflammatory, analgesic, and antipyretic actions by inhibiting the biosynthesis of prostanoids generated from arachidonic acid (AA) via the activity of cyclooxygenase (COX)-1 and COX-2.^12^ COX-1 and COX-2 generate the same prostanoids, but their expression is differently regulated, thus contributing to distinct time and tissue-specific functions in vivo.^13^ Prostanoids act in an autocrine/paracrine manner on their molecular targets through the interaction with G protein-coupled receptors (GPCRs).^14–16^

COX-1 is a housekeeping enzyme constitutively expressed in most tissues, generating prostanoids that are mainly involved in physiological functions, such as gastric cytoprotection and hemostatic integrity.^17^ However, platelet COX-1, by generating thromboxane (TX) A_2_, contributes to atherothrombosis due to its capacity to activate the receptors (named TP) expressed in platelets and vascular cells involved in platelet activation and vasoconstriction. Novel roles of TXA_2_ have emerged in inflammation/fibrosis/tumorigenesis due to its capacity to activate stromal cells such as fibroblasts, thus inducing morphological and functional changes and the induction of COX-2 and its major product, prostaglandin (PG)­E_2_.^18–20^

COX-2, although constitutively expressed in some cells (such as vascular cells), is highly regulated, and its expression can be induced in response to inflammatory and mitogenic stimuli.^17^ However, posttranscriptional regulation of COX-2 expression is also involved in controlling the protein levels in inflammation and cancer.^13^ Enhanced COX-2-dependent PGE_2_ in the tumor microenvironment promotes tumor growth and metastatic spread by inhibiting immunosurveillance and inducing angiogenesis.^21^ Moreover, PGE_2_ can activate tumor epithelial cells causing proliferation, survival, migration/invasion, and epigenetic changes.^21^ However, COX-2, constitutively expressed in the vascular cells, exerts protective effects on the cardiovascular system by generating PGI_2_ (prostacyclin) and activating its receptor IP.^22^ Prostacyclin acts as a general restraint on endogenous stimuli, including platelet COX-1-derived TXA_2_, platelet activation, vascular proliferation and remodelling, hypertension, atherogenesis, and cardiac function.^22^

NSAIDs comprise a chemically heterogeneous family of compounds that are classified into two groups based on their impact on COX-1: the traditional (t)­NSAIDs (also known as nonselective NSAIDs), which affect both COX-1 and COX-2 activity, and the selective COX-2 inhibitors (also known as coxibs).^13^ Coxibs and tNSAIDs share the same clinical efficacy (analgesic and anti-inflammatory effects) due to the inhibition of COX-2; yet, they exhibit different gastrointestinal side effects. Coxibs have a safer gastrointestinal profile due to the sparing of the biosynthesis of COX-1-dependent PGE_2_ and PGI_2_, which have cytoprotective effects on the gastrointestinal system.^23^ In clinical studies, coxibs are associated with a decreased incidence of serious gastrointestinal side effects compared to tNSAIDs, specifically upper gastrointestinal bleeding (UGIB).^24^ Traditional NSAIDs, but not coxibs, inhibit COX-1-dependent prostanoids in the gastrointestinal mucosa in many individuals and are associated with a profound but transient inhibition of COX-1 activity in platelets, thus altering hemostasis in a small number of individuals.^13^

However, both coxibs and tNSAIDs are associated with an enhanced risk of cardiovascular toxicity, including hypertension, edema, heart failure, and thrombotic events.^13,24^ These effects depend on the inhibition of COX-2 in the kidney and vascular cells. It is noteworthy that tNSAIDs, except low-dose aspirin, are not protective for the cardiovascular system either, because of their transient and incomplete inhibition of platelet COX-1-dependent TXA_2_. This pathway must be inhibited almost completely to achieve an antiplatelet effect; indeed, a low concentration of TXA_2_ can result in complete platelet activation induced by subthreshold concentrations of other platelet agonists. Similarly, coxibs do not affect platelet function since platelets do not express COX-2.

Experimental evidence suggests that the role of COX-1-dependent TXA_2_ is in the enhancement of blood pressure (BP) and overload-induced cardiac fibrosis and intestinal inflammation.^18–20^ TXA_2_ produced in platelets induces profibrotic genes, including TGF-β1, and COX-2 expression, a proinflammatory pathway, in myofibroblasts. In a mouse model of salt-sensitive hypertension (high-salt diet in mice with IP deletion [IPKO]), aspirin prevents cardiac myofibroblast accumulation and platelet extravasation in the heart, thereby reducing cardiac fibrosis, when TXA_2_ is unconstrained.^19^ These findings suggest that the inhibition of platelet-derived TXA_2_ by low-dose aspirin could attenuate the hypertensive and profibrotic effects of COX-2 inhibition.

Recently, it has been shown that non-platelet TXA_2_ generation is an independent risk factor for long-term all-cause and cardiovascular mortality in an unselected cohort of older individuals.^25^ Persistent TXA_2_ generation in aspirin users with cardiovascular disease can also originate from non-platelet sources and is linked to adverse outcomes. Unlike low-dose aspirin, which mainly affects platelets, TP receptor antagonists can inhibit the effects resulting from increased TXA_2_ production in both platelet and non-platelet cells without affecting other prostaglandin pathways. Although originally developed as antiplatelet agents, TP receptor antagonists are currently under evaluation in human clinical trials aimed at improving endothelial function, reducing cardiac and pulmonary fibrosis, and suppressing cancer metastases (NCT03962855, NCT03340675, NCT02682511, NCT03694249). Thus, having a new drug that impacts the biological effects of increased TXA_2_ production from both platelets and non-platelet sources could enable coxib use in higher-risk patients and/or for longer periods.

Over the years, the development of new agents as a strategy to mitigate the side effects of NSAIDs, especially gastrointestinal and vascular effects, has been proposed. The first approach involved the development of NSAIDs prodrugs, primarily designed to mitigate gastrointestinal side effects. These prodrugs were obtained by conjugating the acidic function of a selected NSAID with phosphoric acid derivatives (Phospho-NSAIDs),^26^ natural antioxidants,^27,28^ or sugar derivatives through an ester bond.^28^

By employing the polypharmacology-by-design approach,^29^ medicinal chemists have developed several classes of modified multitarget NSAIDs. Multitarget NSAIDs have been designed by joining a COX inhibitor (COXi) to another moiety with appropriate vasodilatory and gastroprotective properties. Among others, COXi/NO-donors (NO-NSAIDs, NO-COXIBs) and COXi/H_2_S donors (HS-NSAIDs) have been obtained.^30–34^ Finally, a two-pronged approach for a multitarget intervention at several levels of the arachidonic acid (AA) cascade was applied.^35^ Examples of this approach are represented by the generation of dual COXi/5-lipoxygenase­(LOX)­i, COXi/leukotriene­(LT)­A_4_Hi, COXi/sEHi and COXi/FAAHi.^36–38^ An alternative two-pronged strategy consists of the inhibition of enzymes and receptors more downstream in the AA cascade, such as sEH/PPARγ or COX-2/TP receptors.^35^

As reported above, the TP receptor is a target of particular interest in the vascular system and platelets.^39^ Although TXA_2_ is the most potent ligand of TP receptor, other prostaglandins can activate this G protein-coupled receptor with varying ranges of potencies.^35,40^ For example, PGE_2_ has been shown to activate TP receptors, eliciting a vasoconstrictor response in rats,^41^ and isoprostanes, nonenzymatic products of AA metabolism that are not blocked by aspirin or NSAIDs, have been demonstrated to contract the rat aorta via TP receptor activation.^42,43^ Paradoxically PGI_2_, a vasodilator prostanoid, can also activate the TP receptor eliciting a vasoconstrictive effect.^44–46^ Finally, the TP antagonist terutroban has been shown to inhibit atherogenesis in Apoe^–/–^ mice^47^ and, in a phase III clinical trial, proved as effective as aspirin in preventing ischemic stroke in the general population and was yet superior in preventing a recurrent event in patients with an history of ischemic stroke.^48,49^

Our research group has recently exploited the design of dual COX-2i/TP antagonists (COXTRANs, i.e. COX inhibitors ThRomboxane ANtagonists).^50^ In our early work, we modulated the structure of lumiracoxib, a selective COX-2 inhibitor showing micromolar activity as TP antagonist obtaining some dual COX-2i/TP antagonists.^51,52^ The obtained prototypes showed a fairly balanced activity, with an improved potency as TP receptor antagonist, although with a reduced ability to block COX enzymes. However, their potencies were still unsatisfactory for in vivo application.

In our ongoing effort, we identified the 2-(1,3,4,9-tetrahydropyrano­[3,4-*b*]­indol-1-yl)­acetic acid scaffold of etodolac (**1**, Chart 1) as a potential privileged template for generating new COXTRANs. This scaffold was chosen due to its high structural similarity to the nonselective TP antagonist laropiprant (MK-0524, **2**) and the selective TP antagonist ramatroban (**3**), both exhibiting a tricyclic annulated indole scaffold. Etodolac is an FDA-approved NSAID used for the treatment of mild to moderate pain, osteoarthritis, and rheumatoid arthritis.^53^ It is endowed with a preferential COX-2 vs COX-1 inhibition, being 8–10-fold more potent on COX-2.^54^ Etodolac was developed in the 1980s, and its structure-anti-inflammatory activity relationship was studied in vivo using a chronic adjuvant arthritis model in rats.

**Chart 1:**
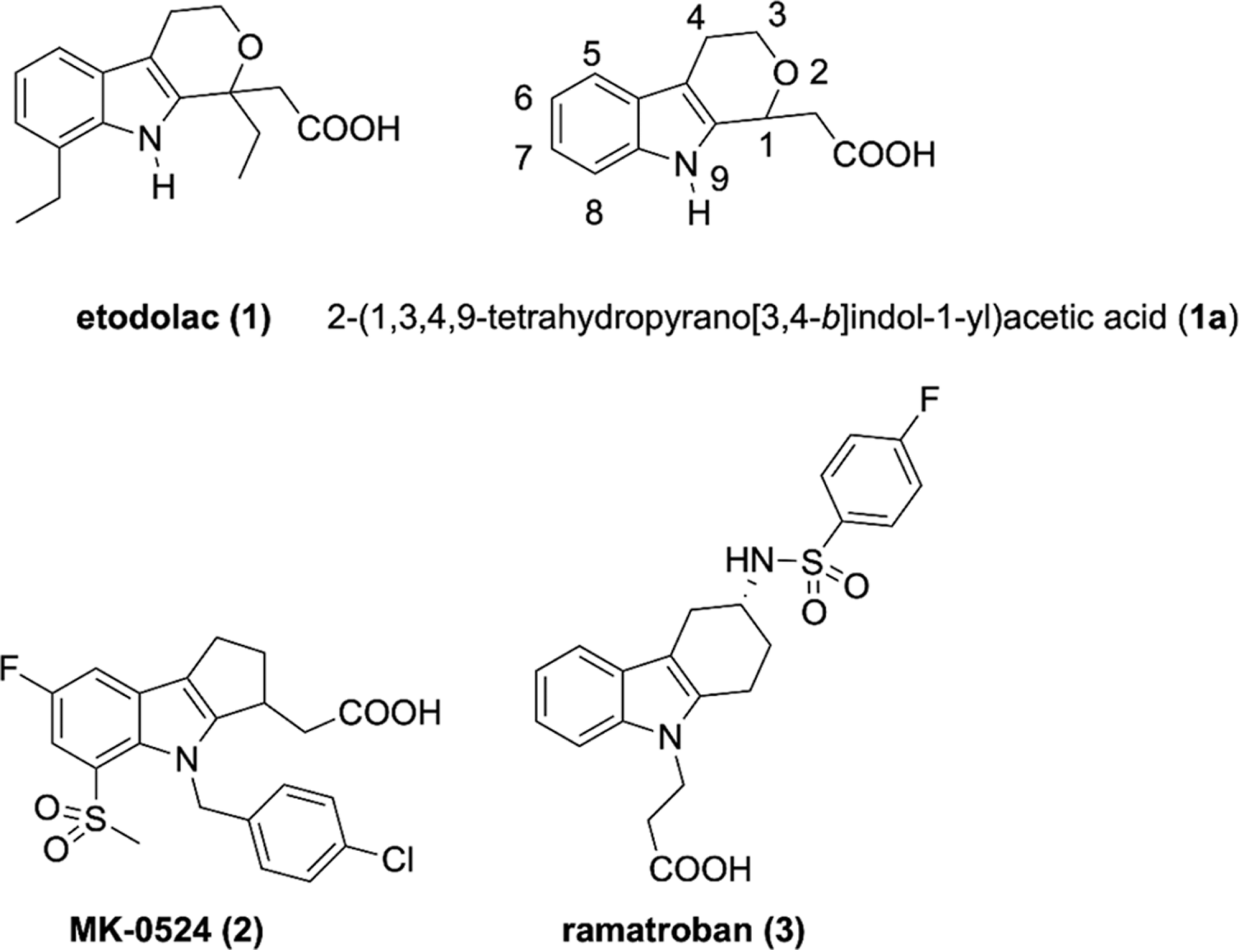
Structure of Reference Compounds Etodolac (**1**), MK-0524 (**2**), Ramatroban (**3**) and of the Chemical Scaffold (**1a**) Modulated in This Study

Taking into account the already established SAR for the anti-inflammatory activity of etodolac^55^ and having some hints from previous modulation of MK-0524 (**2**),^56^ we chose to modulate positions 1, 6, 7, and 8 of the 2-(1,3,4,9-tetrahydropyrano­[3,4-*b*]­indol-1-yl)­acetic acid scaffold (**1a**) in order to obtain new dual COX-2 inhibitors/TP antagonists. A summary of the designed molecules is shown in Figure 1, the logic of the subsequent modulations made will be discussed in the text.

**Figure 1:**
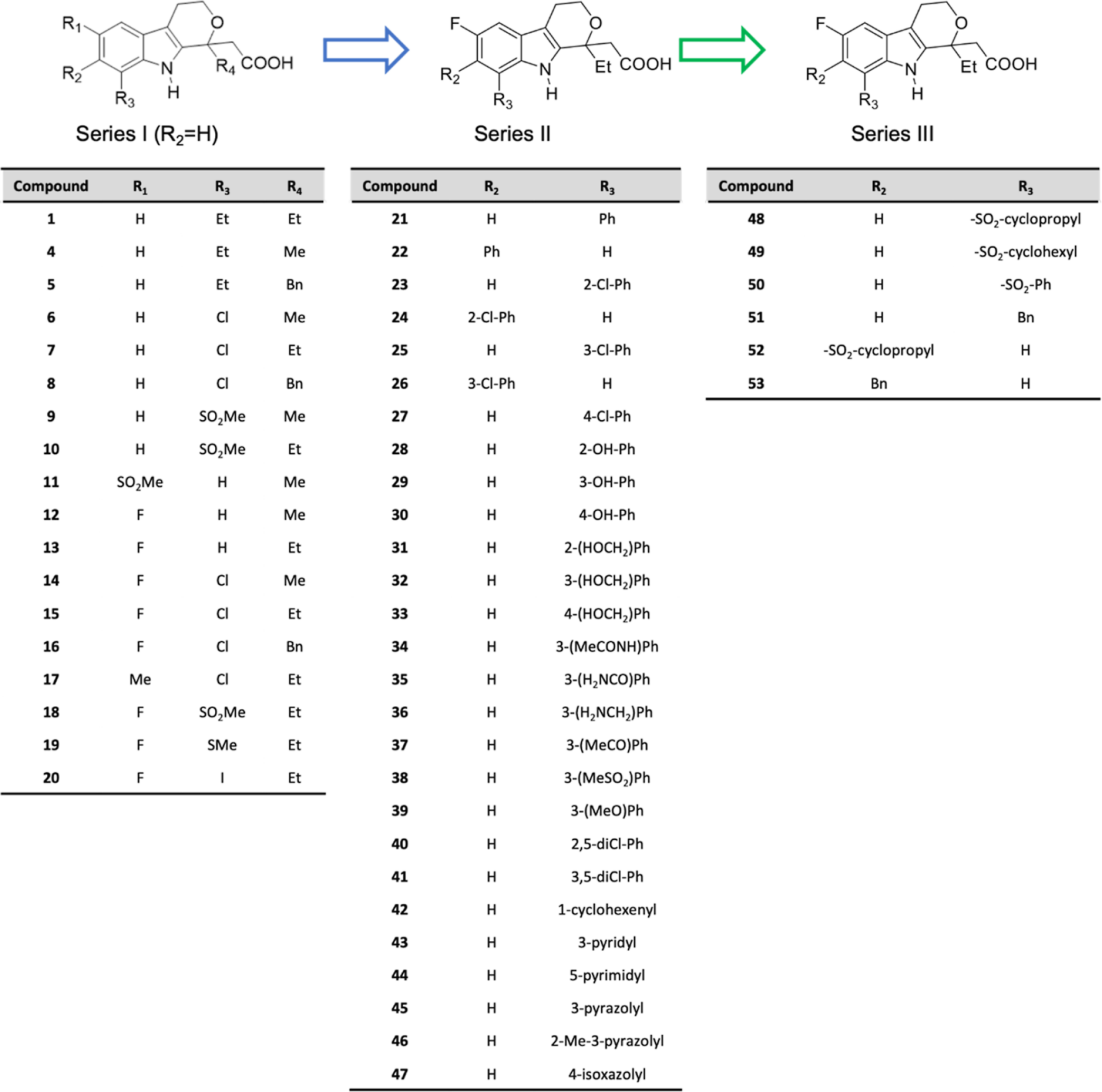
Summary of the designed etodolac derivatives.

In this work, we describe the design, synthesis, in vitro pharmacological screening, and preliminary structure–activity relationships (SAR) of the 2-(1,3,4,9-tetrahydropyrano­[3,4-*b*]­indol-1-yl)­acetic acid scaffold, which enabled us to identify some etodolac derivatives that show both COX-2 inhibition and TP antagonism at nanomolar concentrations in vitro. We also report the physicochemical properties and the inhibition of COXs in human whole blood assays, as well as platelet aggregation, which allowed the selection of compound **51** (CXT29). Finally, the pharmacokinetics, ex vivo characterization of TP antagonism, and in vivo antinociceptive activity after oral administration in mice for the well-balanced **51** are also presented.

## Results and Discussion

### Design and Synthesis of Novel COXTRANs

As the first step, we tested etodolac (**1**) to verify its ability to act as a competitive TP antagonist by measuring the aggregation of human washed platelets stimulated with U-46619, a reference TP agonist, as previously reported.^52^ Consistently with our working hypothesis, the lead compound **1** proved capable of preventing platelet aggregation with a calculated IC_50_ value of 18.42 μM ± 10% CV. COX-1 and COX-2 inhibition, measured through the determination of TXB_2_ in isolated platelets and of PGE_2_ in lymphomonocytes suspension respectively, showed a potent inhibition of COX-2 with an IC_50_ of 0.037 μM ± 17% CV with approximately 70-fold selectivity over COX-1 (IC_50_ = 2.59 μM ± 13% CV) in these test systems (Table 1). To improve the TP antagonism of the lead compound **1**, we designed a series of etodolac analogues. Interestingly, the 2-(1,3,4,9-tetrahydropyrano­[3,4-*b*]­indol-1-yl)­acetic acid scaffold (Chart 1) typical of etodolac shares a certain structural similarity with the 2-(1,2,3,4-tetrahydrocyclopenta­[*b*]­indol-3-yl)­acetic acid scaffold present in MK-0524 which showed a *k*_i_ of 2.95 nM at the TP receptor and was also able to inhibit U-46619-induced platelet-rich plasma (PRP) aggregation (IC_50_ = 0.77 μM).^56^

**Table 1:** TP Antagonism, COX-1 and COX-2 Inhibitory Activities, COX-2 Over COX-1 Selectivity (SI) and TP/COX-2 Balance for Synthesized Compounds **4-20**, Etodolac (**1**) and MK-0524

	TP antagonism	COX-1 inhibition	COX-2 inhibition		
compound	IC_50_ (μM) ± CV %^a^	IC_50_ (μM) ± CV %^b^	IC_50_ (μM) ± CV %^c^	SI^d^	TP/COX-2 balance^e^
**1**	18.4 ± 10%	2.59 ± 13%	0.037 ± 17%	70	497
**4**	15.2 ± 14%	24.7 ± 23%	1.64 ± 17%	15	9.27
**5**	7.88 ± 14%	12.3 ± 27%	0.299 ± 12%	41	26.3
**6**	4.31 ± 9.5%	13.8 ± 19%	0.198 ± 14%	70	21.8
**7**	5.31 ± 34%	1.30 ± 11%	0.018 ± 16%	72	295
**8**	11.0 ± 8%	59 ± 77%	0.44 ± 93%	134	25
**9**	1.64 ± 12%	3.77 ± 46%	10.2 ± 14%	0.44	0.16
**10**	6.12 ± 17%	20.4 ± 18%	0.679 ± 14%	30	9.01
**11**	>100	>100	5.8 ± 63%	ND^f^	
**12**	13.4 ± 9.7%	26.0 ± 48%	14.9 ± 23%	1.7	0.90
**13**	1.40 ± 89%	10.3 ± 42%	inactive	ND^f^	
**14**	1.39 ± 11%	13.0 ± 16%	0.500 ± 25%	26	2.78
**15**	1.79 ± 14%	5.19 ± 13%	0.020 ± 30%	250	90
**16**	6.10 ± 17%	16 ± 44%	0.75 ± 90%	21	8.13
**17**	2.00 ± 31%	7.30 ± 18%	0.039 ± 40%	187	51.3
**18**	3.36 ± 15%	7.93 ± 68%	1.11 ± 13%	7.1	3.02
**19**	1.35 ± 19%	2.77 ± 17%	0.023 ± 29%	120	58.7
**20**	0.57 ± 24%	0.51 ± 9.7%	0.011 ± 15%	46	51.8
**MK-0524**	0.003 ± 24%	9.14 ± 52%	2.84 ± 23%	2.7	0.001

a TP antagonism evaluated as inhibition of platelet aggregation. Compounds were incubated with isolated human platelets for 5 min at 37 °C and platelet aggregation was induced with U-46619 (0.1 μM). Aggregation was monitored with a PAP-8 aggregometer, using the Born turbidimetric assay at 37 °C for 6 min.

b Human platelets from PRP were treated with increasing concentration of test compounds or vehicle. TXB_2_ production was stimulated with the calcium ionophore A23187 (2 μM) for 10 min at 37 °C. TXB_2_ was evaluated in the supernatant by liquid chromatography-tandem mass spectrometry.

c Lympho-monocytes were isolated from buffy coat, washed and suspended in HBSS. The suspension was treated with aspirin (10 μg/mL) and with increasing concentration of test compounds. After LPS-stimulation (10 μg/mL, 24 h), COX-2 activity was evaluated quantifying PGE_2_ production by liquid chromatography-tandem mass spectrometry.

d Selectivity index = IC_50_ COX-1/IC_50_ COX-2.

e Balance = IC_50_ TP/IC_50_ COX-2.

f ND = not determined. All the biological data are expressed as mean ± CV % of 3–6 separate experiments run in triplicate. DMSO used as the cosolvent had no effect.

The first series of compounds (Series I, Figure 1) was designed to explore the role of different substituents at positions 1, 6, and 8 of the 2-(1,3,4,9-tetrahydropyrano­[3,4-*b*]­indol-1-yl)­acetic acid scaffold. A methyl, ethyl or benzyl substituent was used in position 1, a fluorine atom, a methyl and methyl sulfonyl group were used in position 6, while ethyl, chlorine, iodine, methylthio and methylsulfonyl moieties were introduced at position 8. After a preliminary screening, we realized that an ethyl group was the optimal substituent at position 1 and the fluorine atom was preferred at position 6. The screening of the first series of compounds also allowed us to conclude that a lipophilic substituent at position 8 could confer the best pharmacological properties. In the second series (Series II, Figure 1), we kept the ethyl and the fluorine groups at 1 and 6 positions, and modulated position 7 and 8 of the scaffold by direct attachment of differently substituted aromatic, heteroaromatic as well as an unsaturated cycloalkyl ring. In the third series (Series III, Figure 1), we introduced a bridging group connecting the aromatic or cycloalkyl substituent to position 8 in order to increase the flexibility of this part of the molecule.

The final compounds **4–8**, **12–17**, **19–20**, belonging to series I, were synthesized according to the synthetic route depicted in Scheme 1. The substituted anilines **54**–**57** were converted into the corresponding hydrazines using classical diazotization/reduction procedure employing sodium nitrite and tin­(II) chloride in HCl 37% to afford **58–61**, while **62–63** were commercially available. The intermediates **58**–**63** were submitted to a Fisher-type cyclization with 2,3-dihydrofuran to afford the desired 2-(1*H*-indol-3-yl)­ethan-1-ol derivatives **64–69**, while compounds **70–72** were commercially available. This reaction furnished a complex mixture of compounds which required chromatographic purification to isolate the pure intermediates **64**–**69** in low yields (20–30%) over two steps. In attempt to increase the yields and minimize the formation of byproducts, different procedures were employed to catalyze the reaction. However, the use of 4% H_2_SO_4_ in dimethylacetamide (DMA) at 55 °C,^57^ or of a melted mixture of dimethylurea (DMU): tartaric acid (6:4)^58^ or of the hydrazine hydrochloride itself heated at 95 °C did not prove very much different in the outcome of the reaction. After purification, the 2-(1H-indol-3-yl)­ethan-1-ol derivatives were reacted with the appropriate 1,3-dicarbonyl compounds **73–75** in a boron trifluoride diethyl etherate-catalyzed oxa-Pictet-Spengler reaction to afford the substituted methyl 2-(1,3,4,9-tetrahydropyrano­[3,4-*b*]­indol-1-yl)­acetates **76–91**.^59^ Finally, hydrolysis of the methyl ester with LiOH in 1,4-dioxane at room temperature (rt) furnished the final compounds **4–8**, **12–17**, **19** and **20**.

**Scheme 1:**
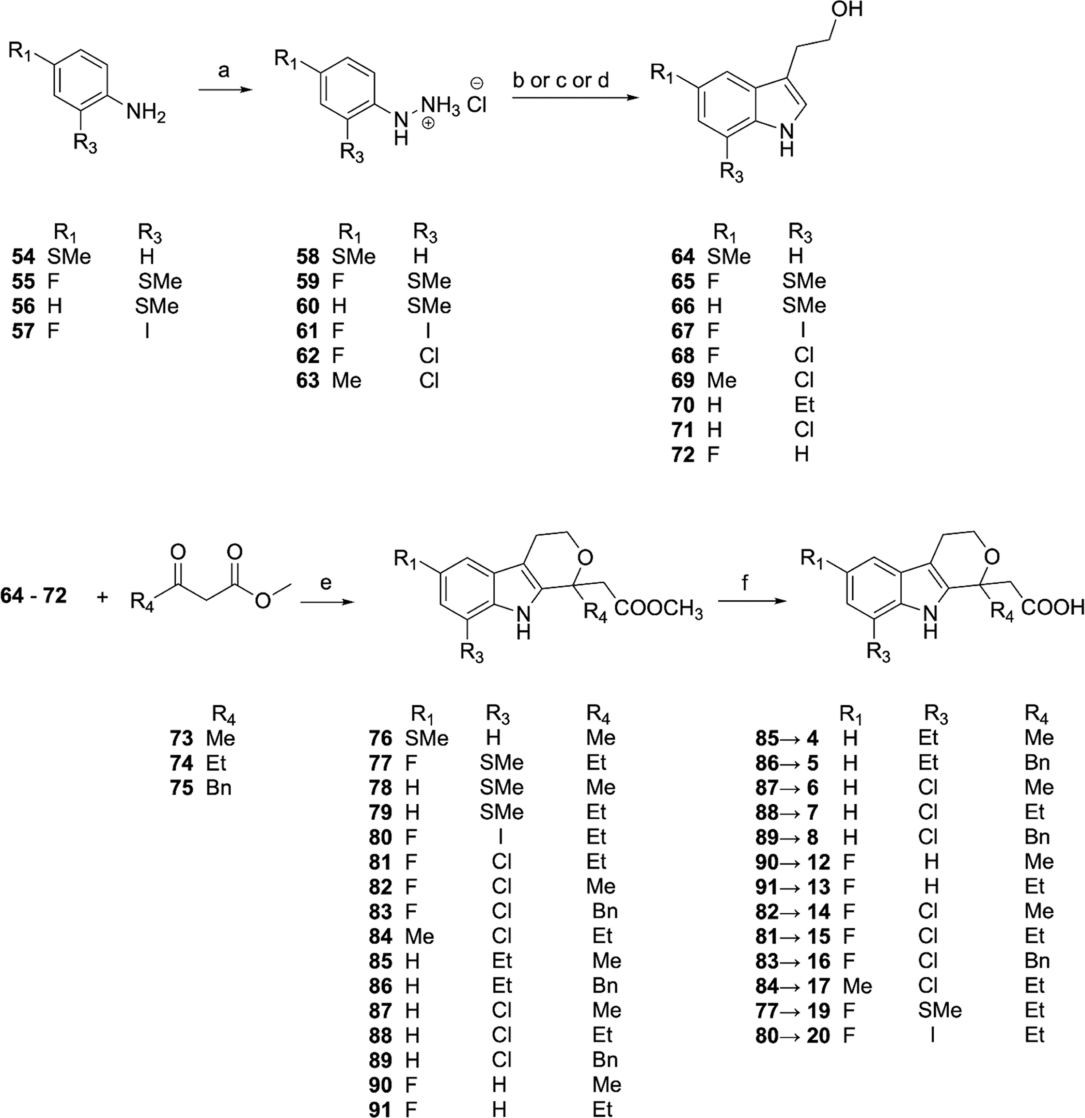
Synthesis of Final Compounds **4–8**, **12–17**, **19–20** ^a^

a Reagents and conditions: (a) (i) NaNO_2_, HCl 37%, 0 °C, 30 min; (ii) SnCl_2_·2H_2_O, HCl 37%, 0 °C, 1 h-3 h; (b) 2,3-dihydrofuran, H_2_SO_4_ 4%/DMA 1:1, 55 °C, 6 h; (c) 2,3-dihydrofuran, DMU/tartaric acid 6:4, 70 °C, overnight; (d) 2,3-dihydrofuran, 1,4-dioxane/H_2_O 10:1, 95 °C, 4 h, (10–49% over two steps); (e) BF_3_·Et_2_O, dry DCM, rt, 5 h, (64–99%); (f) LiOH 3M, 1,4-dioxane, rt, 16 h, (75–100%).

To obtain the methylsulfonyl-substituted compounds **9**–**11** and **18**, the methylthio-substituted intermediates **76**–**79** were oxidized using *meta*-chloroperoxybenzoic acid in dichloromethane and the obtained methyl esters **92**–**95** were hydrolyzed under basic conditions (Scheme 2).

**Scheme 2:**
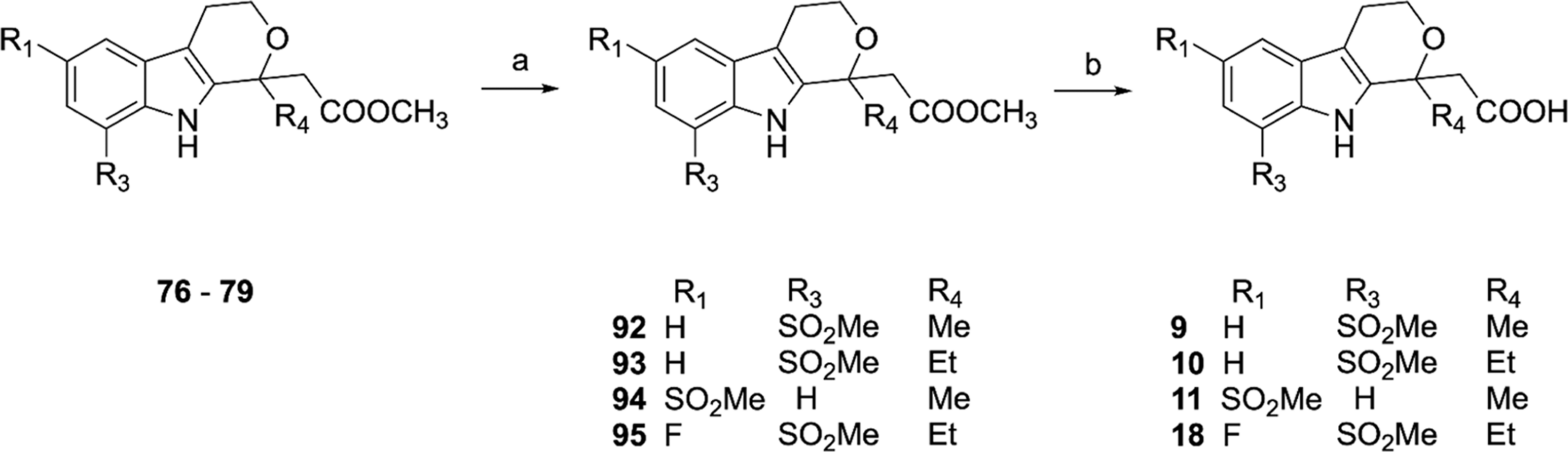
Synthesis of Compounds **9–11** and **18** ^a^

a Reagents and conditions: (a) *m*CPBA, DCM, rt, 18 h, (22–52%); b) LiOH 3M, 1,4-dioxane, rt, 16 h, (43–90%).

Most of the compounds belonging to the second series, bearing a substituted phenyl ring, a heterocyclic ring or the cycloalkenyl substituent at the 8 position of the 1,3,4,9-tetrahydropyrano­[3,4-*b*]­indole scaffold were synthesized by palladium-mediated Suzuki coupling of the iodinated derivative **80** with the appropriate boronic acids (**96a**–**r** or **98a**–**f**) as reported in Schemes 3 and 4. Final compounds **21**, **23**, **25**, **27–41** (Scheme 3), were obtained using either Pd­(dba)_2_, Pd­(PPh_3_)_4_ and Pd­(PPh_3_)_2_(OAc)_2_ as the preferred catalysts in dioxane/water 9/1 mixture, using potassium carbonate or potassium phosphate as the base. In these conditions, the coupling reactions generally proceeded in good yields (75–89%) affording the protected compounds **97a**–**r**, which were subsequently hydrolyzed in basic medium to afford the designed compounds. To obtain the final compound **36**, bearing the aminomethyl group in meta-position of the phenyl ring, the Boc-protected intermediate **97m** was subjected to a first hydrolysis in CF_3_COOH 10% in dichloromethane before the removal of the methyl ester. Compounds **42–47** (Scheme 4) were obtained similarly in 15–76% overall yields from **80**.

**Scheme 3:**
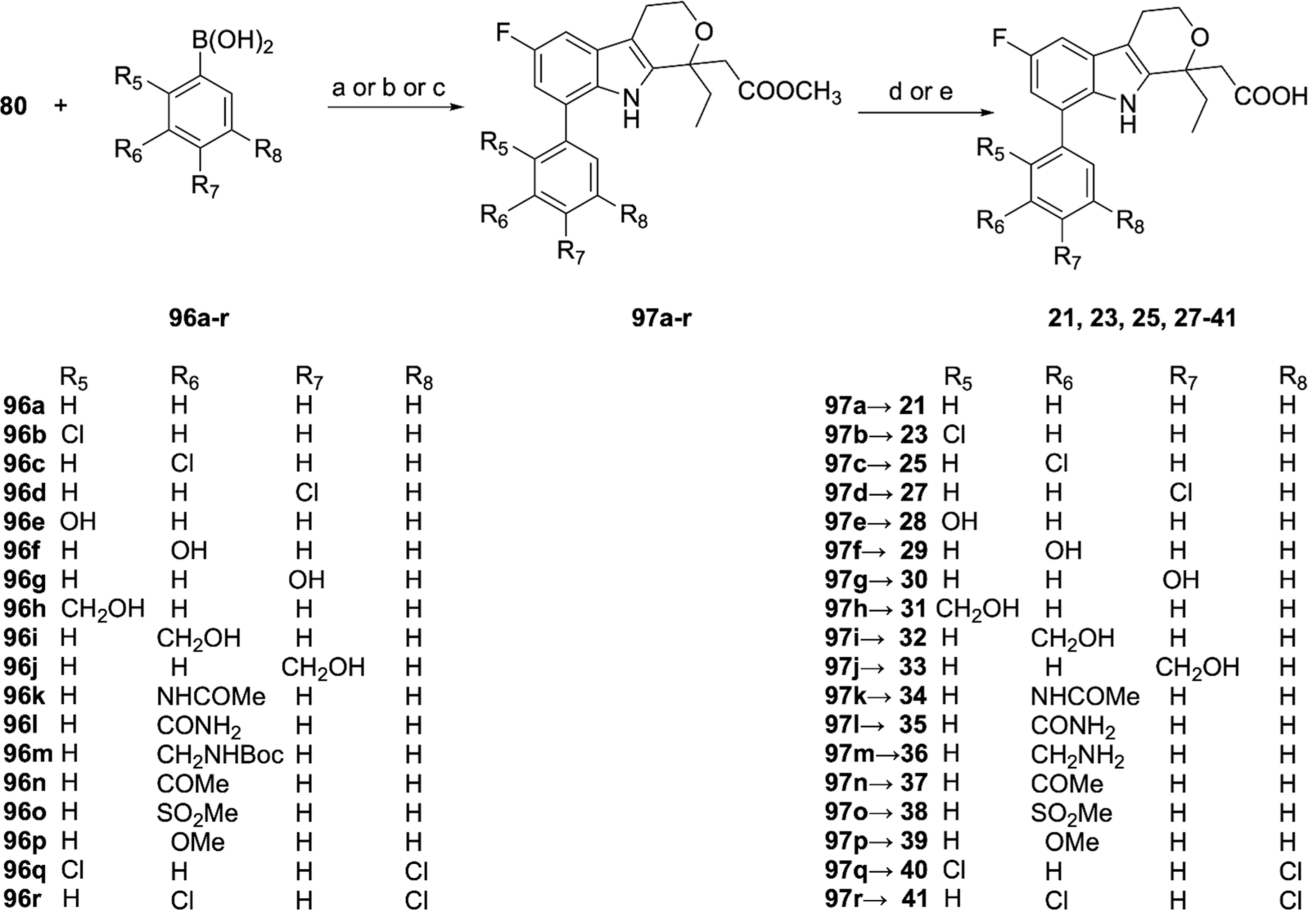
Synthesis of Compounds **21**, **23**, **25**, **27**–**41** ^a^

a Reagents and conditions: (a) K_2_CO_3_, Pd­(dba)_2_, DME/H_2_O 1:1, 90 °C, 30 min, (41–99%); b) Pd­(PPh_3_)_4_, K_2_CO_3_, 1,4-dioxane/H_2_O 9:1, 90 °C, 3 h, (95%); c) Pd­(PPh_3_)_2_(OAc)_2_, K_3_PO_4_, 1,4-dioxane/H_2_O 9:1, 90 °C, 2–3 h, (75–89%); d) for **97m** (i) TFA, DCM, rt, 45 min, (66%); (ii) LiOH 3M, 1,4-dioxane, rt, 16 h, (95%); e) for **97a–l**, **97n–r**: LiOH 3M, 1,4-dioxane, rt, 16 h, (41–100%).

**Scheme 4:**
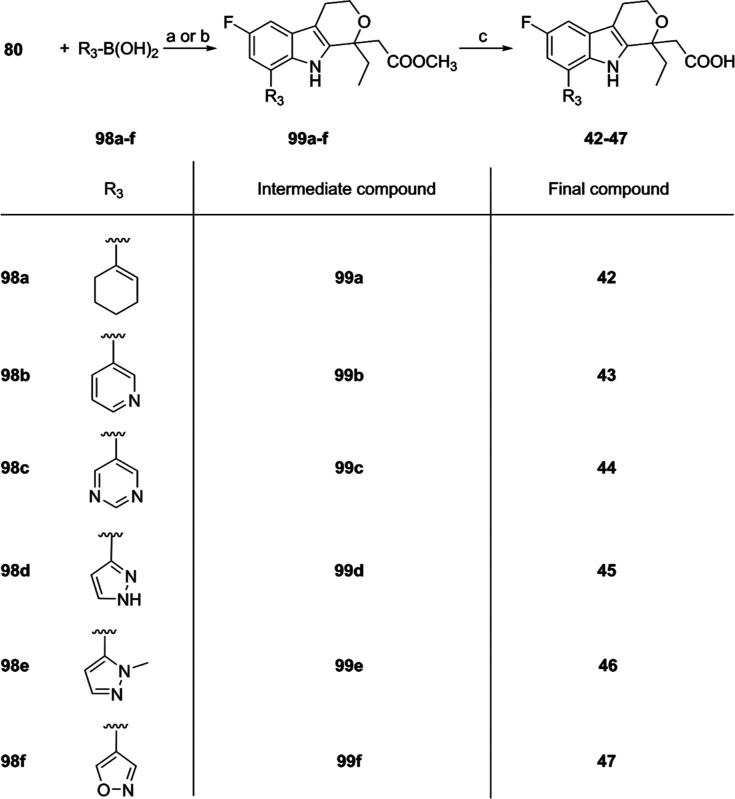
Synthesis of Compounds **42–47** ^a^

a Reagents and conditions: (a) K_2_CO_3_, Pd­(dba)_2_, 1,4-dioxane/H_2_O 9:1, 90 °C, 2 h, (31%); b) Pd­(PPh_3_)_2_(OAc)_2_, K_3_PO_4_, 1,4-dioxane/H_2_O 9:1, 90 °C, 2–3 h, (37–92%); c) LiOH 3M, 1,4-dioxane, rt, 16 h, (48–98%).

The cyclopropylsulfonyl- and cyclohexylsulfonyl-substituted compounds **48** and **49** were obtained by copper­(II) acetate-mediated nucleophilic substitution of iodine on compound **20** using the corresponding sulfinic acids **100** and **101** in DMSO at 120 °C (Scheme 5A). The harsh reaction conditions gave rise to a complex mixture which required difficult chromatographic purification or preparative HPLC to obtain the desired pure final compounds. The 2-(1-ethyl-6-fluoro-8-(phenylsulfonyl)-1,3,4,9-tetrahydropyrano­[3,4-*b*]­indol-1-yl)­acetic acid (**50**) was synthesized through a similar procedure; in this case, sodium benzenesulfinate (**102**) was reacted with the 2-(5-fluoro-7-iodo-1*H*-indol-3-yl)­ethan-1-ol **67** to afford the indolyl-ethanol intermediate **103** in fair yields (56%). This intermediate was then cyclized to **104** and hydrolyzed by the usual procedure to afford the desired **50** in good yields (Scheme 5B). The synthesis of 2-(8-benzyl-1-ethyl-6-fluoro-1,3,4,9-tetrahydropyrano­[3,4-*b*]­indol-1-yl)­acetic acid (**51**), bearing a benzyl group in position 8, was achieved by Suzuki coupling of **80** with benzyl boronic acid **105**; in this case, the use of Pd­(dppf)­Cl_2_ as the catalyst was required to obtain reasonable yields (44%) of the coupled product **106**, which was then hydrolyzed to **51** (Scheme 5C). All the synthesized final compounds showed a purity higher than 95% by HPLC analysis (see Supporting Information).

**Scheme 5:**
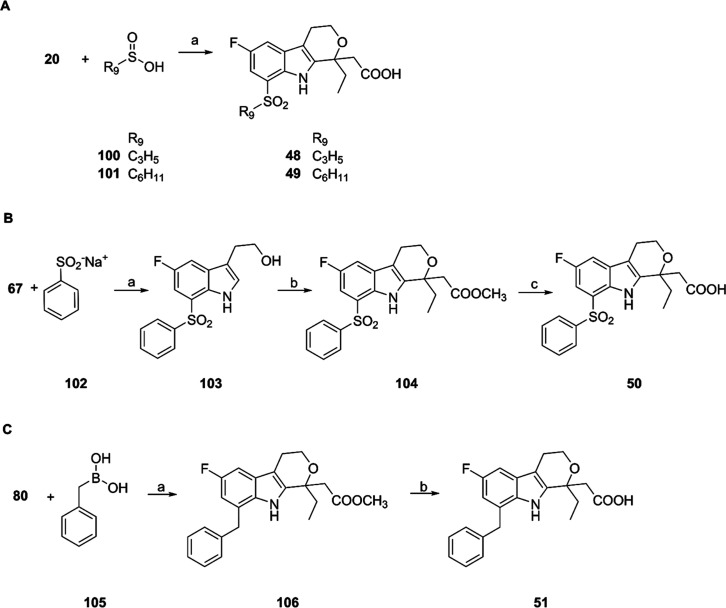
Synthesis of Compounds **48–51** ^a^

a Reagents and conditions. Panel A: (a) Cu­(OAc)_2_, NaOH 5M, DMSO, 120 °C, 3–4 h, (15–78%); Panel B: (a) Cu­(OAc)_2_·H_2_O, NaOH 5M, DMSO, 120 °C, 4 h, (56%); (b) **74**, BF_3_·Et_2_O, dry DCM, rt, 5 h, (56%); (c) LiOH 3M, 1,4-dioxane, rt, 16 h, (84%); Panel C: (a) Pd­(dppf)­Cl_2_, K_2_CO_3_, 1,4-dioxane/H_2_O 9:1, 90 °C, 18 h, (44%); (b) LiOH 3M, 1,4-dioxane, RT, 16 h, (75%).

The final compounds **22**, **24**, **26**, **52** and **53**, substituted at position 7 of the 1,3,4,9-tetrahydropyrano­[3,4-*b*]­indole scaffold, were synthesized according to a different route (Scheme 6). The commercially available 6-bromo-5-fluoro-1*H*-indole (**107**) was reacted with oxalyl chloride and the obtained intermediate **108** was reduced with borane-dimethyl sulfide complex to afford the indolyl-ethanol derivative (**109**). Ring closing with methyl propionyl acetate (**74**) afforded the methyl 2-(7-bromo-1-ethyl-6-fluoro-1,3,4,9-tetrahydropyrano­[3,4-*b*]­indol-1-yl)­acetate (**110**) that was submitted to Suzuki coupling with boronic acids **96a**–**c** to give compounds **111a**–**c** in satisfactory yields (44–74%). Hydrolysis by LiOH afforded the desired **22**, **24**, and **26**. To obtain compound **52**, the intermediate **110** was hydrolyzed to the corresponding acid **112** and reacted with cyclopropyl sulfinic acid **100** using copper­(I) iodide in *N*-methyl pyrrolidone (NMP) at 150 °C to afford a complex mixture from which **52** was recovered by preparative HPLC. Finally, coupling of **110** with benzyl boronic acid **105** using the same reaction conditions described for **106** (Scheme 5C) and basic hydrolysis afforded the 2-(7-benzyl-1-ethyl-6-fluoro-1,3,4,9-tetrahydropyrano­[3,4-*b*]­indol-1-yl)­acetic acid (**53**).

**Scheme 6:**
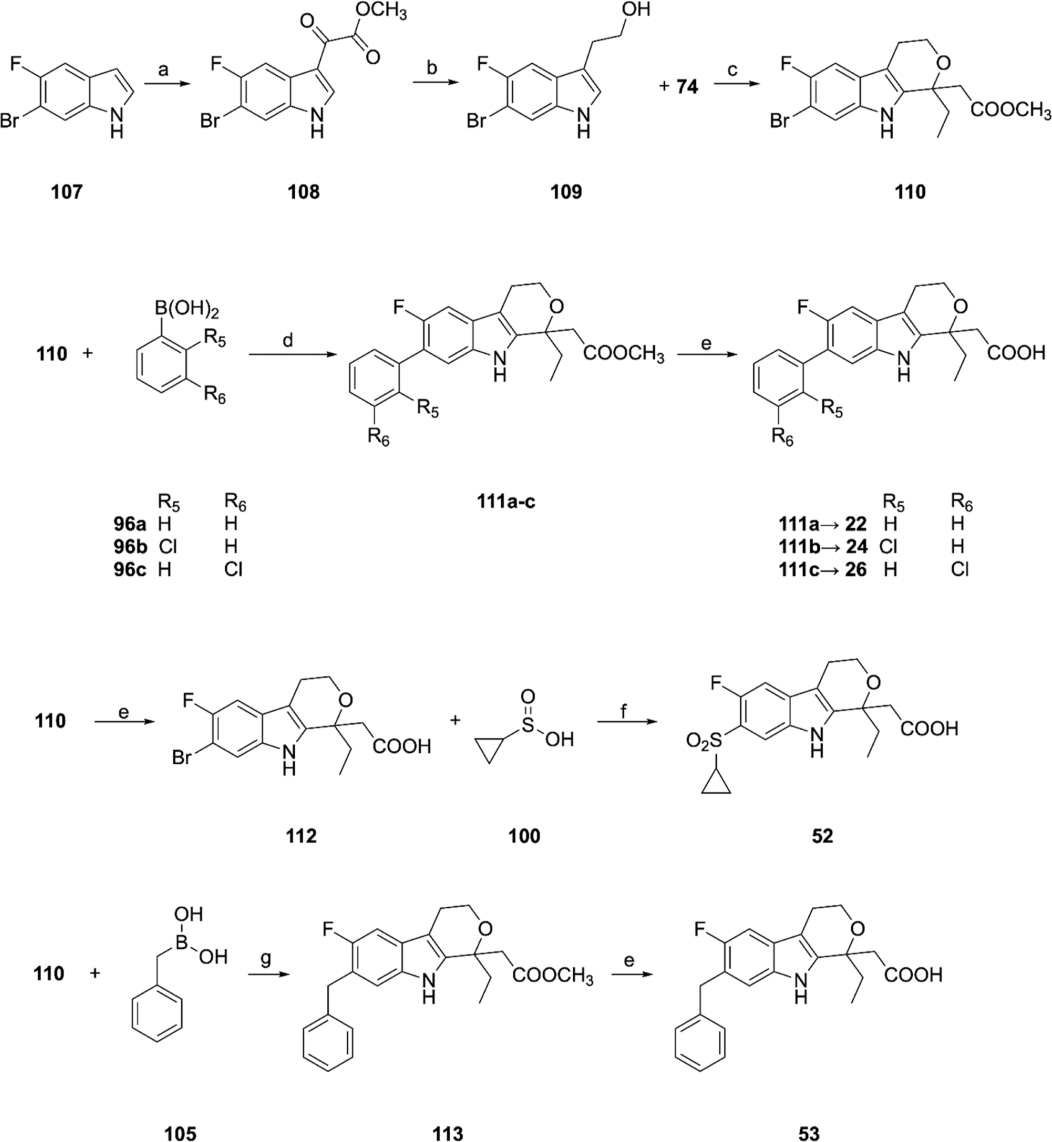
Synthesis of Compounds **22**, **24**, **26**, **52** and **53** ^a^

a Reagents and conditions: (a) Oxalyl chloride, Et_2_O, rt, 1 h then MeOH, rt, 24 h, (76%); (b) BH_3_·SMe_2_, THF, reflux, 4 h, (45%); (c) **74**, BF_3_·Et_2_O, CH_2_Cl_2_, rt, 5 h, (67%); (d) Pd­(PPh_3_)_2_(OAc)_2_, K_3_PO_4_, 1,4-dioxane/H_2_O 9:1, 90 °C, 2–3 h, (44–74%); (e) LiOH 3M, 1,4-dioxane, rt, 16 h, (12–92%); (f) CuI, NMP, 150 °C, 18 h, (25%); (g) Pd­(dppf)­Cl_2_, K_2_CO_3_, 1,4-dioxane/H_2_O 9:1, 90 °C, 18 h, (71%).

### Assessment of TP Antagonism and Cyclooxygenases Inhibition

All the synthesized compounds were assessed for their ability to act as TP antagonists on washed platelets from healthy human volunteers, in which the TPα isoform is extensively expressed.^60^ Washed platelets samples were challenged with U-46619 (0.1 μM), a stable TXA_2_ analogue, to induce platelet aggregation as previously reported.^52^ Compounds at different concentrations (1 nM to 100 μM) or vehicle (DMSO max 0.2% v/v) were incubated for 5 min before addition of U-46619. The antiaggregating activity of the compounds, reported as IC_50_ ± CV % is collected in Tables 1–3. The ability of the synthesized compounds to inhibit COX-1 and COX-2 activity was measured in platelets and lymphomonocyte suspensions, respectively. To measure COX-1 inhibition, human platelets were isolated from platelet-rich plasma (PRP) by centrifugation and incubated with increasing concentrations of test compounds (1 nM to 100 μM) or vehicle for 30 min. The production of TXB_2_, which in this system is dependent on COX-1 activation, was triggered by the calcium ionophore A23187 (2 μM; 10 min) and the TXB_2_ released in the supernatant was evaluated by liquid chromatography-tandem mass spectrometry using the isotopic dilution of the internal standard [d_4_]­TXB_2_. For the determination of COX-2 inhibition, lympho-monocytes obtained from buffy coat were washed and resuspended in Hank’s Balanced Salt Solution (HBSS). The suspension was treated with increasing concentration of test compounds (1 nM to 100 μM, 30 min). COX-2-dependent PGE_2_ production was then triggered with LPS (10 μg/mL; 24 h). PGE_2_ production was quantified by liquid chromatography tandem mass spectrometry using [d_4_]­PGE_2_ as the internal standard. The obtained results, expressed as IC_50_ values for COX-1 and COX-2 inhibition, are collected in Tables 1–3. The selectivity toward COX-2 (SI), calculated as the ratio of the IC_50_s for COX-1 and COX-2, is also reported.

**Table 2:** TP Antagonism, COX-1 and COX-2 Inhibitory Activities, COX-2 Over COX-1 Selectivity (SI) and TP/COX-2 Balance for Synthesized Compounds **21–47** and Etodolac (**1**)

compound	TP-antagonism inhibition of platelet aggregation	COX-1 inhibition	COX-2 inhibition	SI^d^	TP/COX-2 balance^e^
	IC_50_ (μM) ± CV %^a^	IC_50_ (μM) ± CV %^b^	IC_50_ (μM) ± CV %^c^		
**1**	18.4 ± 10	2.59 ± 13	0.037 ± 17	70	497
**21**	0.086 ± 34	0.055 ± 9.2	0.070 ± 45	0.78	1.23
**22**	21.90 ± 15	NT	NT		
**23**	0.38 ± 19	0.097 ± 42	0.18 ± 12	0.54	2.1
**24**	26.4 ± 30	NT	NT		
**25**	0.034 ± 44	0.088 ± 4.8	0.047 ± 17	1.9	0.72
**26**	12.68 ± 9	NT	NT		
**27**	0.15 ± 32	0.118 ± 22	0.086 ± 34	1.4	1.70
**28**	0.22 ± 38	6.31 ± 25	1.58 ± 13	3.99	0.14
**29**	0.12 ± 26	0.86 ± 9	0.41 ± 17	2.1	0.29
**30**	0.389 ± 16	14.06 ± 28	NT		
**31**	0.11 ± 38	92.2 ± 38	16.8 ± 32	5.5	0.0065
**32**	0.085 ± 19	12.2 ± 14	0.783 ± 17	16	0.11
**33**	0.31 ± 21	209 ± 36	1.41 ± 30	148	0.22
**34**	0.026 ± 44	19.5 ± 6.7	7.13 ± 14	2.7	0.0036
**35**	0.180 ± 30	27.6 ± 12	0.649 ± 16	42.5	0.28
**36**	0.18 ± 54	NT	inactive@10 mM	ND	
**37**	0.072 ± 18	0.58 ± 10	0.61 ± 26	0.95	0.12
**38**	0.239 ± 12	16.2 ± 28	1.25 ± 23	12.9	0.19
**39**	0.189 ± 20	0.46 ± 19	0.29 ± 17	1.6	0.65
**40**	0.034 ± 17	2.55 ± 13	0.282 ± 31	9.0	0.12
**41**	0.016 ± 12	1.37 ± 25	0.132 ± 48	10	0.12
**42**	0.119 ± 8.0	0.089 ± 17	0.072 ± 12	1.2	1.65
**43**	0.358 ± 6	3.13 ± 43	0.45 ± 51	6.9	0.79
**44**	1.48 ± 11	9.47 ± 30	0.36 ± 95	26	4.11
**45**	2.58 ± 38	59.2 ± 51	0.39 ± 28	151	6.61
**46**	0.410 ± 11	6.76 ± 69	0.21 ± 40	32	1.95
**47**	2.16 ± 94	39.6 ± 18	0.075 ± 32	528	28.8

a TP antagonism evaluated as inhibition of platelet aggregation. Compounds were incubated with isolated human platelets for 5 min at 37 °C and platelet aggregation was induced with U-46619 (0.1 μM). Aggregation was monitored with a PAP-8 aggregometer, using the Born turbidimetric assay at 37 °C for 6 min.

b Human platelets from PRP were treated with increasing concentration of test compounds or vehicle. TXB_2_ production was stimulated with the calcium ionophore A23187 (2 μM) for 10 min at 37 °C. TXB_2_ was evaluated in the supernatant by liquid chromatography-tandem mass spectrometry.

c Lympho-monocytes were isolated from buffy coat, washed and suspended in HBSS. The suspension was treated with aspirin (10 μg/mL) and increasing concentration of test compounds. After LPS-stimulation (10 μg/mL, 24 h), COX-2 activity was evaluated quantifying PGE_2_ production by liquid chromatography-tandem mass spectrometry.

d Selectivity index = IC_50_ COX-1/IC_50_ COX-2.

e Balance = IC_50_ TP/IC_50_ COX-2. All the biological data are expressed as mean ± CV % of 3–6 separate experiments run in triplicate. NT = not tested. DMSO used as the cosolvent had no effect.

**Table 3:** TP Antagonism, COX-1 and COX-2 Inhibitory Activities, COX-2 Over COX-1 Selectivity (SI) and TP/COX-2 Balance for Synthesized Compounds **48-53** and Etodolac (**1**)

	TP-antagonism inhibition of platelet aggregation	COX-1 inhibition	COX-2 inhibition		
compound	IC_50_ (μM) ± CV %^a^	IC_50_ (μM) ± CV %^b^	IC_50_ (μM) ± CV %^c^	SI^d^	TP/COX-2 balance^e^
**1**	18.4 ± 10%	2.59 ± 13%	0.037 ± 17%	70	497
**48**	0.395 ± 35%	1.67 ± 11%	0.060 ± 45%	28	6.58
**49**	0.11 ± 18%	81.0 ± 57%	0.44 ± 14%	184	0.25
**50**	0.15 ± 13%	18.7 ± 16%	0.19 ± 14%	98	0.79
**51 (CXT29)**	0.096 ± 14%	0.722 ± 15%	0.013 ± 12%	56	7.38
**52**	Inactive@30 μM	NT	NT		
**53**	14.2 ± 13%	0.28 ± 61%	0.076 ± 12%	3.7	186

a TP antagonism evaluated as inhibition of platelet aggregation. Compounds were incubated with isolated human platelets for 5 min at 37 °C and platelet aggregation was induced with U-46619 (0.1 μM). Aggregation was monitored with a PAP-8 aggregometer, using the Born turbidimetric assay at 37 °C for 6 min.

b Human platelets from PRP were treated with increasing concentration of test compounds or vehicle. TXB_2_ production was stimulated with the calcium ionophore A23187 (2 μM) for 10 min at 37 °C. TXB_2_ was evaluated in the supernatant by liquid chromatography-tandem mass spectrometry.

c Lympho-monocytes were isolated from buffy coat, washed and suspended in HBSS. The suspension was treated with aspirin (10 μg/mL) and increasing concentration of test compounds. After LPS-stimulation (10 μg/mL, 24h), COX-2 activity was evaluated quantifying PGE_2_ production by liquid chromatography-tandem mass spectrometry.

d Selectivity index = IC_50_ COX-1/IC_50_ COX-2.

e Balance = IC_50_ TP/IC_50_ COX-2. All the biological data are expressed as mean ± CV % of 3–6 separate experiments run in triplicate. NT = not tested. DMSO used as the cosolvent had no effect.

The analysis of the results obtained from the first series of compounds (Table 1, compounds **4**–**20**) gave a useful indication for the development of COXTRANs. The introduction of a methyl, ethyl or benzyl group as the R_4_ substituent in position 1 (compounds **1**, **4** and **5**) of the 1,3,4,9-tetrahydropyrano­[3,4-*b*]­indole scaffold indicated that the use of the ethyl group in this position is optimal for preserving COX-2 inhibition, while the benzyl substitution apparently slightly increases TP antagonism. When these substitutions were performed on derivatives bearing a chlorine atom as the R_3_ substituent at position 8 of the 1,3,4,9-tetrahydropyrano­[3,4-*b*]­indole scaffold (compounds **6**–**8**) the same observations applies for COX-2 and COX-1 inhibition: compound **7**, was 11 to 24-fold more potent than **6** and **8** respectively, and also maintained a good in vitro COX-2 selectivity. Looking at the TP antagonism, the use of a chlorine atom allowed to increase the potency (3-fold increase with respect to **1**) albeit still not enough to obtain a balanced multitarget compound. The use of a methylsulfonyl group as the R_3_ substituent (compounds **9** and **10**) as in MK-0524 prototype, proved detrimental for COX inhibition, while the same group shifted to position 6 (R_1_ in compound **11**) abolished the activity both at the TP receptor and against COXs. We then designed and tested compounds **12**–**16** where a fluorine atom was introduced at position R_1_. The obtained results indicated that when no substitution is present at position R_3_, the introduction of a fluorine atom alone had no effect (compounds **12** and **13**) as both the compounds showed a reduced potency as COXi. However, the concomitant introduction of a chlorine atom in position R_3_ and a fluorine in R_1_ conferred an interesting profile to this class of 1,3,4,9-tetrahydropyrano­[3,4-*b*]­indole derivatives. Compound **15** was 10-fold more potent than etodolac as TP antagonist (IC_50_ 1.79 vs 18.4 μM) and maintained the same potency against COX-2 showing also an improved selectivity vs COX-1. Also, among compounds bearing the 6-chloro-8-fluoro-disubstitution (**14**–**16**), the ethyl group at position 1 proved superior to the use of a methyl or a benzyl group, especially with respect to COX-2 inhibition; therefore, we decided to use the ethyl group as the preferred R_4_ substituent in further development of this class of compounds. We then synthesized compounds **17**–**20** by modulating the R_1_ and R_3_ positions. The use of a methyl group in place of a fluorine atom at position 6 (R_1_ substituent) gave **17**, which showed similar properties with respect to **15**. In compounds **18**–**20**, the R_3_ substituent was modulated using the methylsulfonyl, methylthio or iodine residues; the obtained results showed that an increase in lipophilicity (cLogP 1.42, 3.29, 3.90 for **18**, **19**, **20**, respectively) in this position appears favorable to confer TP antagonism while conserving COX-2 inhibition and selectivity, with compound **20** showing a 32-fold increased TP antagonism with slightly improved COX-2 inhibition with respect to **1**. Overall, the synthesis and the pharmacological screening of the first series of etodolac derivatives allowed us to draw some preliminary SAR for dual TP antagonism and COX-2 inhibition. The use of R_1_ = F, R_4_ = Et is favorable for activity both at the TP receptor and at COX-2, while the use of a lipophilic R_3_ residue improves TP antagonism.

On this basis, we designed and synthesized compound **21**, bearing a phenyl ring at position 8 (Figure 1, Table 2). When tested for TP antagonism, **21** proved very potent, being over 200-fold more potent than etodolac with an IC_50_ value of 0.086 ± 34% μM for the inhibition of U-46619-stimulated platelet aggregation. The ability of **21** to inhibit COX-1 and COX-2 activity also laid in the nanomolar range (COX-1 IC_50_ = 0.055 ± 9.2% and COX-2 IC_50_ = 0.070 ± 45% μM); unfortunately, the selectivity on COX-2 was completely lost. The introduction of a chlorine atom in the ortho, meta and para position of the phenyl ring (compounds **23**, **25** and **27**) showed that *m*-chloro substitution was tolerated, while substitutions at other positions decreased TP antagonism compared to **21** (Table 2). These derivatives also demonstrated a good inhibition of COXs activity with compounds **21** and **25** showing the highest potency; however, the selectivity toward COX-2 was negligible (Table 2). When the phenyl, the *o*-chlorophenyl and the *m*-chlorophenyl rings were shifted to the 7 position of the 1,3,4,9-tetrahydropyrano­[3,4-*b*]­indole scaffold (R_2_ substituent in Figure 1) obtaining derivatives **22**, **24** and **26**, a large drop in the TP antagonism was evidenced. Introduction of a hydroxyl or hydroxymethyl group in any position of the benzene ring (compounds **28–33**) resulted in a slight decrease in TP antagonism with respect to **21** or **25**. Interestingly in these three series of positional isomers the meta-substitution proved the more favorable for TP antagonism. Moreover, the *m*-hydroxymethyl-substituted compound (**32**) also retained a certain degree of selectivity toward COX-2 inhibition, therefore, we further explored this position by introduction of larger substituents endowed with different lipophilicity and hydrogen bonding properties. Compounds **34**–**39** showed submicromolar IC_50_ values for TP antagonism with *m*-acetamido- (**34**) and *m*-acetyl-substituted (**37**) being the most potent. However, only **35**, **37** and **39** showed submicromolar inhibition of COX-2; among them, **35** proved quite selective vs COX-2. However, **35** was approximately 17-fold less active than reference **1** as a COX-2 inhibitor. The introduction of a second chlorine atom on the phenyl ring to obtain 2′,5′- or 3′,5′-substituted compounds **40** and **41** gave rise to potent TP antagonists endowed with submicromolar potencies as COX-2 inhibitors. Both compounds demonstrated a preferential COX-2 inhibition (SI = 10), however, they showed about eightfold greater ability to inhibit TP receptor with respect to COX-2 and a reduced COX-2 inhibition with respect to **1** as judged by their IC_50_ values. We then explored the introduction of other R_3_ substituents. The use of a cycloalkenyl ring (**42**) or different heterocyclic rings (**43**–**47**) in this position furnished compounds with a mixed profile. Among them **42**, **43** and **46** were submicromolar inhibitors of both targets, however still not possessing an ideal profile because of the lack of COX-2 vs COX-1 selectivity as in the case of compounds **42** and **43**. Interestingly, the introduction of the 4-isoxazolyl substituent (**47**) gave a compound with a satisfactory potency against COX-2 and good selectivity (IC_50_ = 0.075 μM; SI = 528) albeit with poor activity at TP receptor.

Finally, we decided to insert a bridging group between the lipophilic tail and the position 8 of the 1,3,4,9-tetrahydropyrano­[3,4-*b*]­indole scaffold synthesizing compounds **48**–**51** (series III, Figure 1). We reasoned that this modulation should confer an increased flexibility to this part of the molecule thus potentially allowing an easier fit of the lipophilic moiety in the binding pockets. The synthesized compounds revealed an interesting profile as the introduction of a cyclopropylsulfonyl (**48**), a cyclohexylsulfonyl (**49**), a phenylsulfonyl (**50**) or a benzyl (**51**) residue conferred a submicromolar potency at the TP receptor (Table 3) with compounds **48**, **50** and **51** maintaining also an ability to inhibit COX-2 similar to that of the reference **1** (Table 3). Also in this case, the shift of the best performing substituents to the 7-position of the 1,3,4,9-tetrahydropyrano­[3,4-*b*]­indole scaffold (compounds **52** and **53**) abrogated the TP antagonism, despite the greater flexibility of the introduced moiety.

Examination of the concentration–response curve for some selected compounds with a fairly increased potency for TP antagonism with respect to etodolac (>100 fold) and a reasonable balance activity between TP antagonism and COX-2 inhibition, led us to focus on two compounds, i.e. **50** and **51** (Table 3 and Figures 2 and 3) for further evaluation and studies. It is clear that, despite other compounds had similar or sometimes better parameters for TP antagonism or COX-2 potencies (e.g., **21**, **25**, **27**, **32**, **42**, **48**), they lacked in selectivity between COX-1 and COX-2 and, therefore, were not further considered.

**Figure 2:**
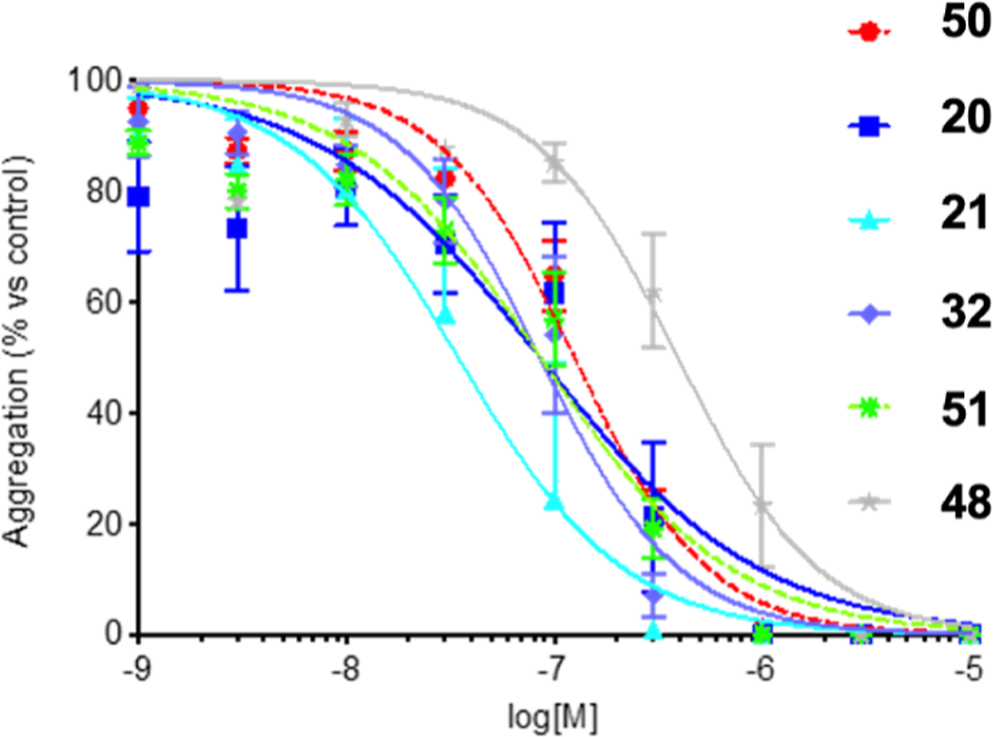
Antagonism of human platelet aggregation induced by U-46619 by the indicated compounds. Concentration-inhibition curves of the indicated compounds in washed platelet aggregation from human blood. Values shown represent platelet aggregation (mean ± SE) expressed as % maximal aggregation induced by 10 μM U-46619. Experiments have been performed at least three times in duplicates. All curves shown were computer-generated using GraphPad Prism v.5.

**Figure 3:**
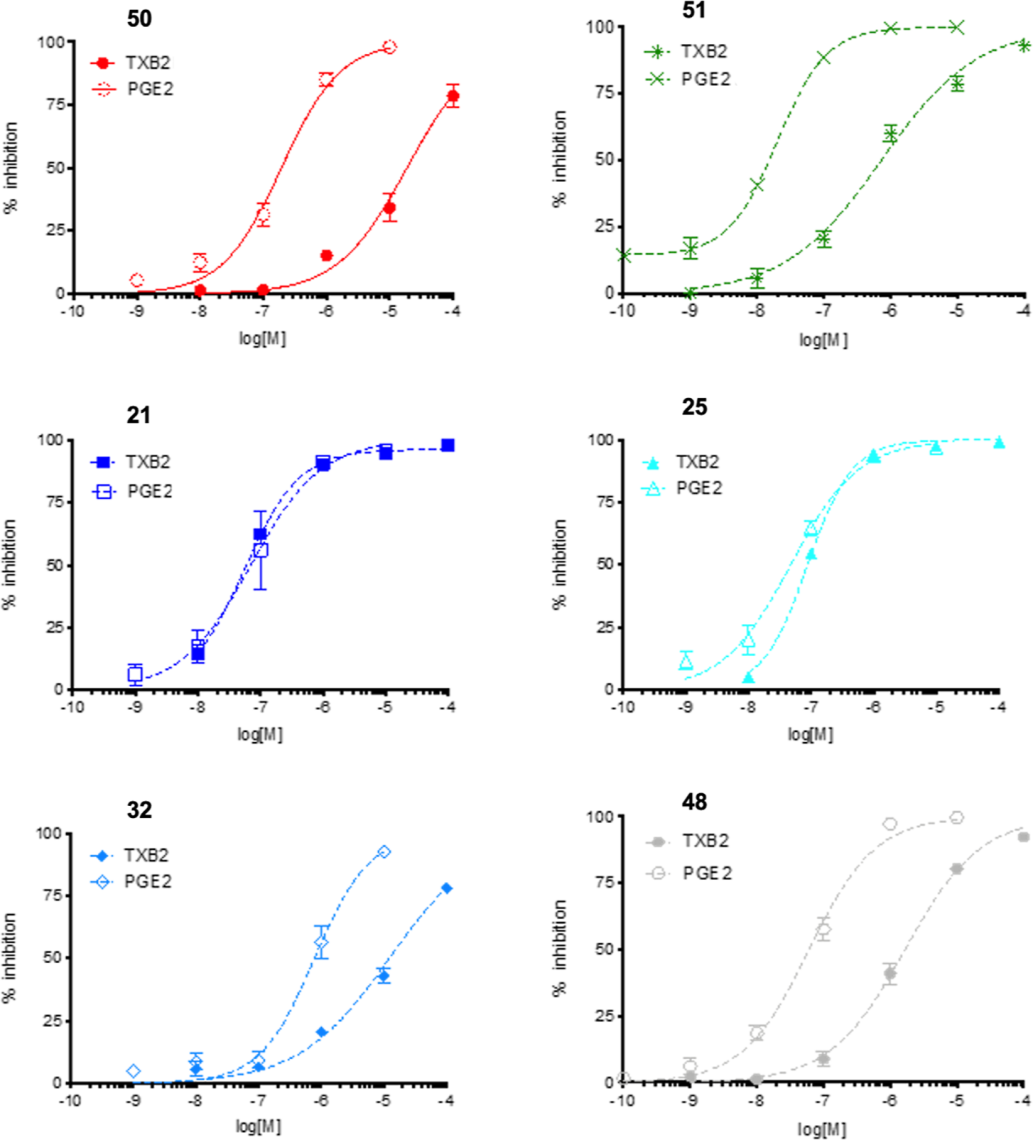
Inhibition of COX-1 and COX-2 activity by the indicated compounds. COX-1 activity was assessed in terms of inhibition of TXB_2_ production induced by calcium ionophore A23187 in human washed platelets; COX-2 activity was assessed in terms of inhibition of PGE_2_ production induced by LPS in isolated human monocytes. Data are expressed as percent inhibition of TXB_2_ or PGE_2_ release versus untreated controls. Error bars represent mean ± SE of at least three independent experiments, each performed in duplicate. All curves shown were computer generated using GraphPad Prism v.5.

While this work was in progress, a crystal structure of ramatroban (Chart 1, compound **3**) bound to human TP receptor (PDB: 6IIU) was published.^61^ Here we propose a binding mode of the etodolac series in complex with the TP receptor, which is exemplified by one of the most potent TP antagonists of the series, compound **21** (Figure 4A). The acidic headgroup is tightly involved in ionic and directed H-bond interactions toward amino acids Arg295, Ser181, and His89. The annulated indole core nicely fits into the flat lipophilic pocket formed by Val85. The phenyl moiety points toward a deep hydrophobic pocket indicating additional space which can be used by lipophilic substituents. This observation fits to the SAR of the 8-position, which tolerated a wide variety of lipophilic substituents, while substituents in the 7-position were not tolerated due to steric restriction. Although the benzylic group in the 8-position of compound **51** exhibits different geometry, it is also suitable to occupy the deep hydrophobic pocket of the TP receptor (Figure 4B).

**Figure 4:**
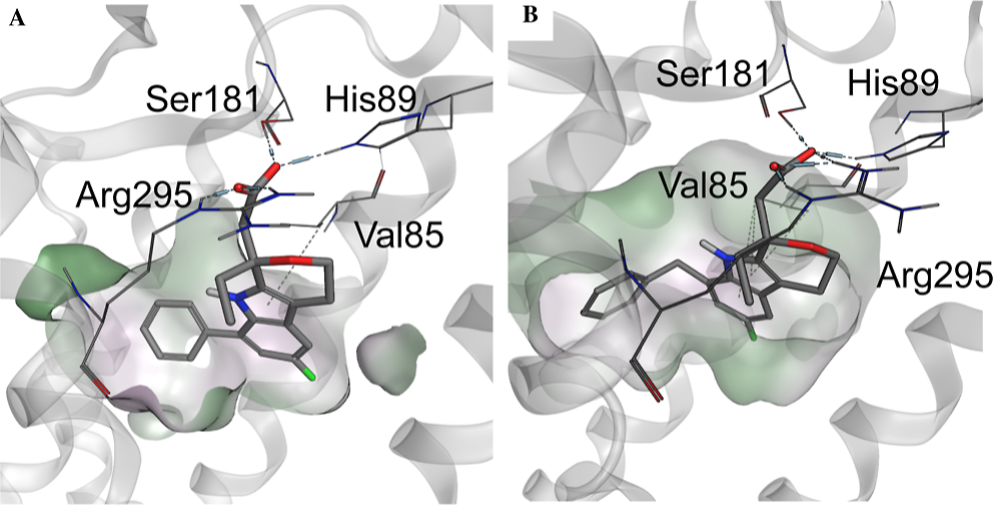
Proposed binding modes of COXTRANs to the TP receptor (PDB: 6IIU). (A) Compound **21** in complex with TP receptor. (B) Compound **51** in complex with TP receptor.

We also proposed the binding mode of compound **51** in complex with COX-2 based on the cocrystal structure of diclofenac (PDB: 1PXX).^62^ The acidic headgroup is involved in directed H-bond interactions toward Ser530 and Tyr385 (Figure 5). Furthermore, like diclofenac, the intramolecular hydrogen bond toward the indole NH is formed. The indole scaffold fits tightly in the flat pocket formed by Ala527. In contrast to the lipophilic pocket of TP receptor, the COX-2 binding pocket offers additional space for various substitution patterns, which is reflected by the SAR.

**Figure 5:**
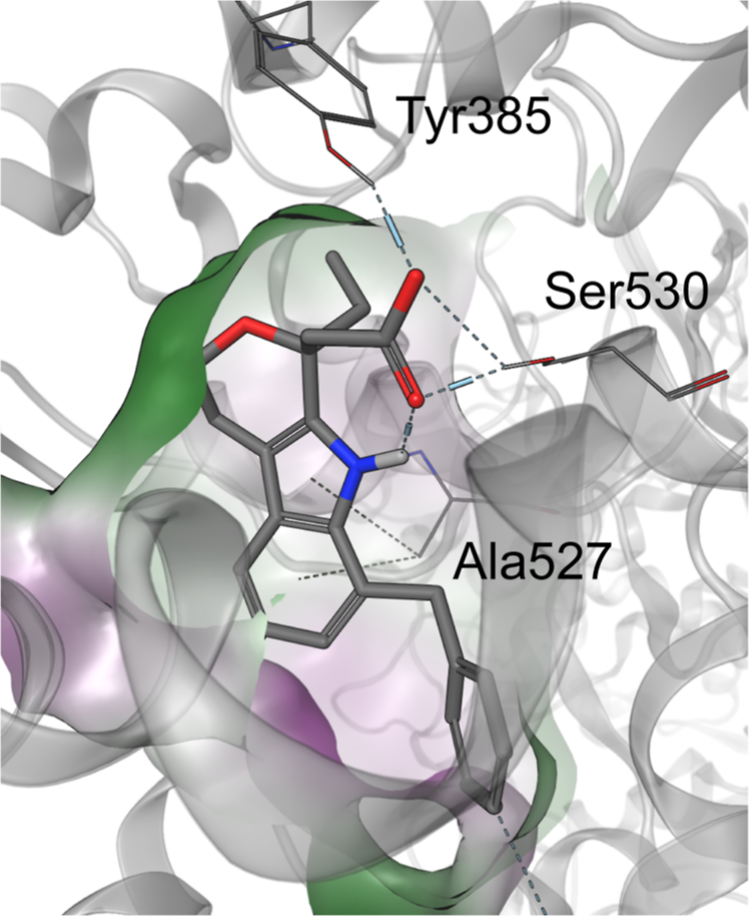
Proposed binding pose of compound **51** into COX-2 binding pocket (PDB: 1PXX).

### TP Antagonism in Rat Aorta

The TP antagonist effects were also confirmed in a different pharmacological model. The TP antagonism for representative compounds **21**, **25**, **32**, **48**, **50** and **51**, selected on the basis of their biological activities, was determined in isolated rat aortic rings stimulated with U-46619 according to a previously published procedure with some modifications.^52^ The rat aortic rings were pretreated with indomethacin to block endogenous prostanoid formation. Cumulative concentration–response curves for U-46619 were established in the absence (control) or in the presence of selected compounds, added to the organ bath fluid 20 min before the concentration–response curves for U-46619 were determined. All responses were expressed as percent of maximum contraction, induced by KCl 50 mM. For each inhibitor the pA_2_ value was calculated (Figure 6).

**Figure 6:**
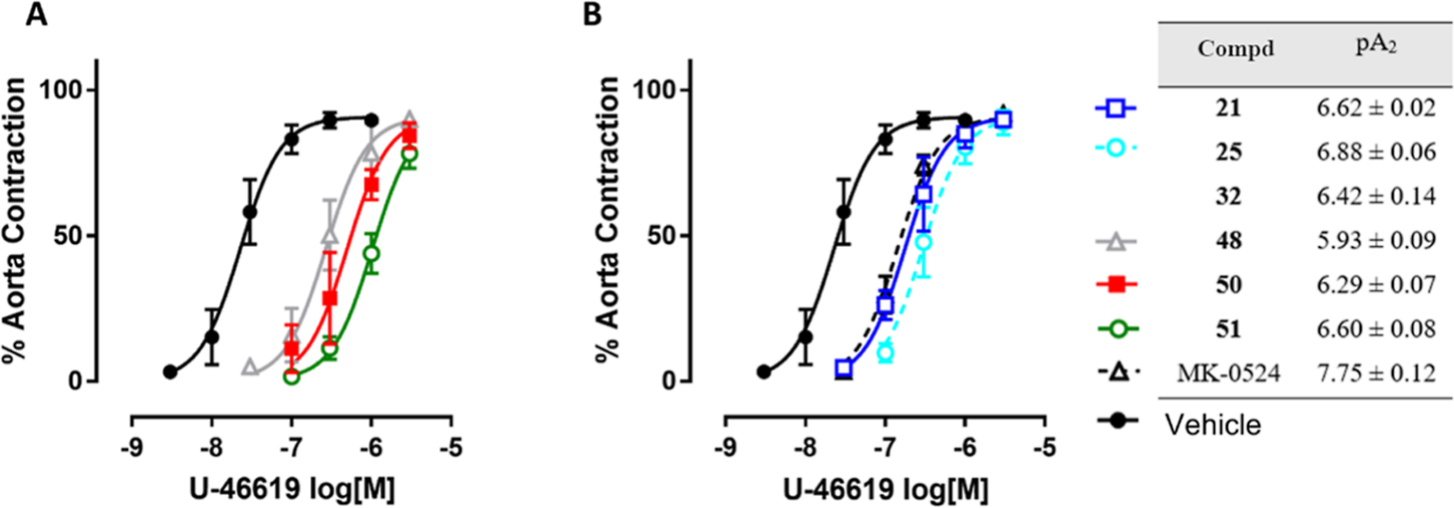
(A): Evaluation of TP antagonism in isolated rat aortic rings pretreated with 10 μM indomethacin and contracted with U-46619 in the presence of compounds **48** (10 μM), **50** (10 μM), **51** (10 μM) or vehicle alone (control). (B): Evaluation of TP antagonism in isolated rat aortic rings pretreated with 10 μM indomethacin and contracted with U-46619 in the presence of compounds **21** (1 μM), **25** (1 μM), MK-0524 (0.1 μM, dotted line) or vehicle alone (control). Error bars represent mean ± SEM of at least three independent experiments. Curves were computer generated from the simultaneous analysis of independent experiments using GraphPad Prism version 7.0. pA_2_ values were calculated using Gaddum equation: pA_2_ = log­[CR – 1] – log­[*B*], where CR = ratio of EC_50_ with and without antagonist; [*B*] = antagonist concentration. The concentration–response curve for compound **32** is omitted for clarity.

All the compounds were able to right-shift the concentration–response curve for U-46619, with different potencies. The potencies expressed by pA_2_ values ± SEM were calculated with the Gaddum equation. In this assay, compounds **25**, **21**, and **51** (pA_2_ = 6.88 ± 0.06; 6.62 ± 0.02; 6.60 ± 0.08, respectively) proved more potent than **32** and **50** (pA_2_ = 6.42 ± 0.14; 6.29 ± 0.07) followed by **48** (pA_2_ = 5.92 ± 0.08). The antagonism for etodolac was not measurable in the test conditions, while MK-0524 confirmed its potency with a pA_2_ of 7.75 ± 0.12.

### Physico-chemical Properties

The dissociation constant (p*K*_a_), solubility at physiological pH, lipophilic-hydrophilic balance (*c* log *P* and log *D* ^7.4^) and plasma protein binding for selected compounds **21**, **25**, **32**, **48**, **50**, **51** and etodolac (**1**), were determined.^52^ The carboxylic acid group in etodolac was reported to possess a p*K*_a_ value of 4.65,^63^ in agreement with this result, we measured a p*K*_a_ of 4.63 ± 0.01 for etodolac. As expected, all the compounds showed similar p*K*_a_ values lying in the 4.59–4.68 range, consequently they are largely deprotonated at physiological pH. As can be seen in Table 4, the logarithm of distribution coefficient of the compounds measured at pH 7.4 (log *D* ^7.4^) is about two units lower than the log *P* value calculated for the neutral form (*c* log *P*), in agreement with the ionization profile of the compounds. The differences observed in terms of hydrophilic–lipophilic balance between the compounds are therefore dependent on their structural features more than on their ionization profile. As reported in Table 4, all the selected compounds, with the exception of compound **48**, showed an increased lipophilicity with respect to the reference **1**. The solubility of compounds in pH 7.4 buffered solution was about 10-fold lower than that of **1** and was roughly inversely related to their lipophilicity. Conversely, plasma protein binding measured in fresh human serum was unaffected with respect to that showed by the reference compound **1**.

**Table 4:** Lipophilicity (*C* log *P*, log *D* ^7.4^), Solubility and Plasma Protein Binding for the Synthesized Compounds **21**, **25**, **32**, **48**, **50**, **51** and Etodolac (**1**)

compound	*C* log *P* ^a^	Log *D* ^7.4^ ^b^	solubility^c^ (mg/mL) ± SD	plasma protein binding^d^ (% bound)
**1**	3.43	0.67 ± 0.07	5.1 ± 0.02	99.5
**21**	4.60	2.24 ± 0.08	0.30 ± 0.03	99.5
**25**	5.32	2.52 ± 0.06	0.16 ± 0.01	99.6
**32**	3.56	1.48 ± 0.03	0.65 ± 0.06	99.5
**48**	2.01	0.62 ± 0.03	0.89 ± 0.08	99.4
**50**	3.35	1.54 ± 0.05	0.35 ± 0.04	99.4
**51**	4.78	2.09 ± 0.08	0.53 ± 0.07	99.6

a *C* log *P* were calculated with Bio-Loom for Windows, Vers. 1.5 (BioByte).

b The partition coefficients between *n*-octanol and phosphate buffer 50 mM, pH 7.4 (ionic strength adjusted to 0.15 M with KCl) was obtained by shake-flask technique at room temperature: compound was solubilized in the buffered aqueous phase and appropriate amounts of *n*-octanol was added; the two phases were shaken for about 20 min and then centrifuged (10,000 rpm, 10 min). The concentration of the solute was evaluated in the aqueous phase by UV spectrophotometer (UV-2501PC, Shimadzu); each log *D* value is an average of at least six measurements.

c Each solid compound (2 mg) was added to 1 mL buffered solution (50 mM of phosphate buffered saline at pH 7.4) and the resulting suspension was shaken at 25 °C for 24 h. The suspension was filtered through a PTFE 0.45 μm filter (VWR) and analyzed by HPLC (HP 1100 chromatograph system Agilent Technologies, Palo Alto, CA, USA) for compound quantification; experiments were done in triplicate.

d Plasma protein binding determination was achieved by ultrafiltration using commercially available membrane systems (Centrifree ultrafiltration devices with ultracel YM-T membrane, Merck): 1 mL of the solution of compound in human serum was inserted in the sample reservoir of the ultrafiltration device and gently shaken at 37 °C for 1 h; tube was then centrifuged (1000*g* for 25 min) and the concentration of the compound in the ultrafiltrate and filtrate was determined by RP-HPLC; experiments were done in triplicate (SEM < 0.1).

### COX Inhibition in Human Whole Blood and TP Antagonism in Platelet-Rich Plasma

The synthesized compounds **50** and **51**, selected for their biological activity and physicochemical properties, were evaluated for their ability to act as dual COX-2 inhibitors and TP antagonists in blood cells in the presence of plasma proteins. These assays have the advantage of (i) using whole blood cells and (ii) providing a physiological plasma protein level; therefore, the results help to establish the activity level and the balance between the different desired pharmacological activities of a multitarget drug in a more reliable way compared to in vitro testing.

We first evaluated the inhibitory effects of **50** and **51** on COX-1 and COX-2 activities in human whole blood assays, which are considered the gold standard methods for assessing biochemical COX-2 selectivity. These assays are based on the measurement of PGE_2_ production in response to a 24 h incubation of LPS with heparinized blood samples, which reflects the time-dependent induction of COX-2 in circulating monocytes.^64^ A parallel measurement of TXB_2_ production during whole blood clotting is used as an index of platelet COX-1 activity.^63^ For the COX-2 assay, different concentrations (0.01–40 μM) of **50** and **51** or DMSO vehicle were incubated with heparinized whole blood samples in the presence of LPS at 37 °C for 24 h. Plasma was separated by centrifugation and subsequently assayed for PGE_2_, as previously described.^65^

For the COX-1 assay, different concentrations (0.01–40 μM) of **50** and **51** or DMSO vehicle were incubated with aliquots of peripheral blood and allowed to clot at 37 °C for 60 min. The serum was separated by centrifugation and then assayed for TXB_2_, as previously described.^63^ Prostanoids were measured in serum or plasma using previously described and validated immunoassays.^66^

As shown in Figure 7A, **50**, at 40 μM, caused a maximal inhibition of 71 ± 17% (mean ± SEM, *n* = 4) of LPS-induced PGE_2_ biosynthesis in whole blood without significantly affecting thrombin-stimulated TXB_2_ production. Compound **51** caused a concentration-dependent inhibition of LPS-induced PGE_2_ production with an IC_50_ value of 4.11 μM. The compound was less potent in affecting thrombin-stimulated TXB_2_ production. At 40 μM, whole blood COX-1 activity was reduced by 44 ± 16% (mean ± SEM, *n* = 6). Thus, **51** was characterized by a COX-1/COX-2 IC_50_ ratio >97.32 (Figure 7B).

**Figure 7:**
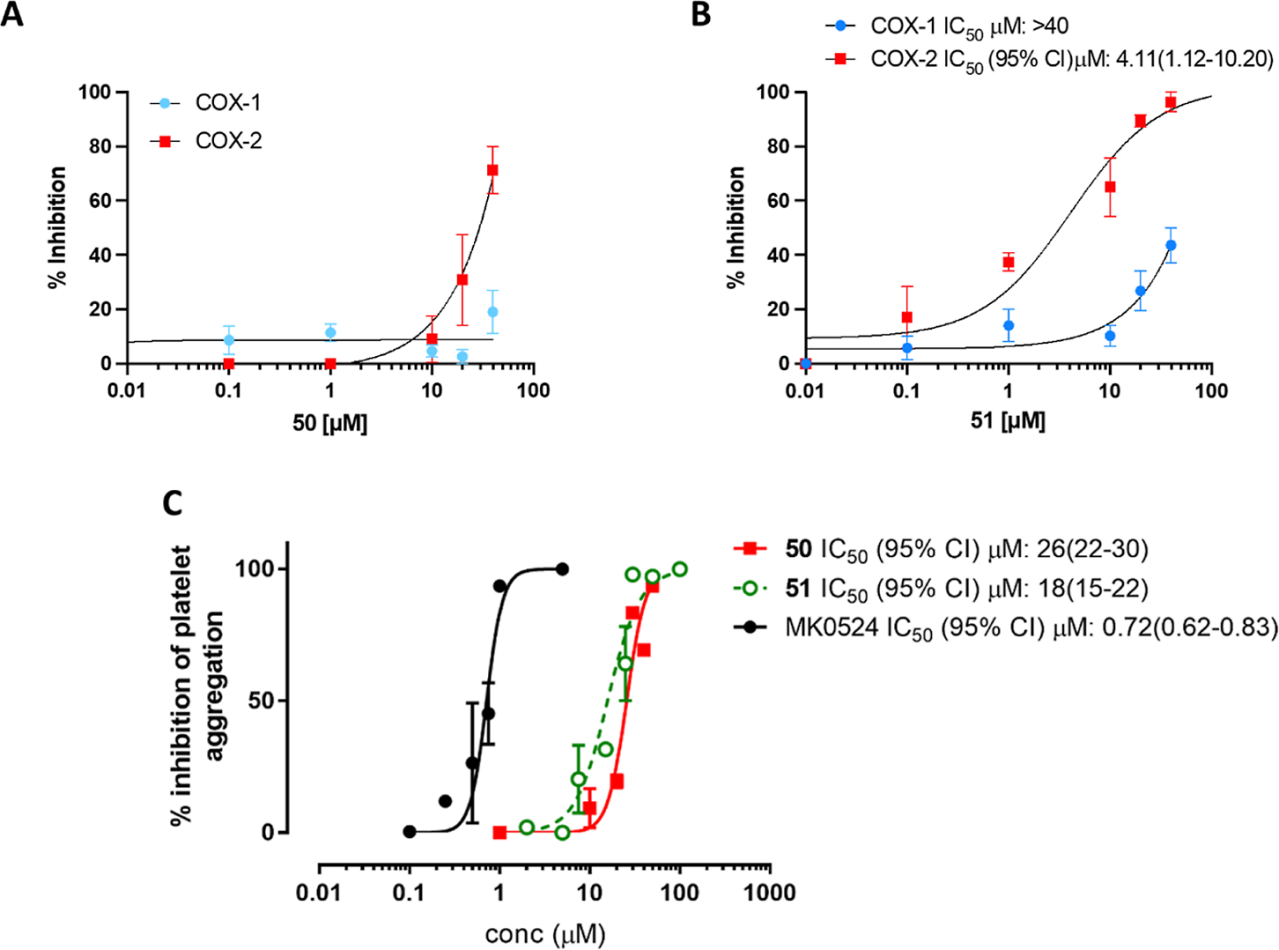
(A,B) Concentration–response curves for COX-1 and COX-2 inhibition by compounds **50** and **51** in human whole blood. For the COX-2 assay, PGE_2_ production in response to a 24 h incubation of LPS with heparinized blood samples was measured. For the COX-1 assay, TXB_2_ production during whole blood clotting was measured. Different concentrations of compounds or DMSO vehicle were incubated, and the percentage (%) of inhibition of prostanoid generation compared to the vehicle was assessed. (C) Antiaggregatory activity of compounds MK-0524, **50**, and **51** on human PRP. Platelet aggregation was induced by U-46619 in PRP samples preincubated with tested compounds or vehicle alone (control samples). The antiaggregatory activity of the compounds was assessed as the percentage inhibition of maximal platelet aggregation induced by U-46619 in the presence of the vehicle.

The ability of **50** and **51** to inhibit platelet aggregation in human platelet-rich plasma (PRP) was then assessed. Human blood treated with citrate solution and with acetylsalicylic acid (10 μg/mL) was centrifuged to obtain PRP samples. Compounds under study or vehicle (DMSO 0.5%) were incubated in aliquots of PRP at different concentrations (1–100 μM) for 10 min before the addition of the aggregating stimulus at 37 °C. The aggregation was triggered with U-46619, and PRP was kept under continuous stirring at 37 °C and monitored for 10 min using Light Transmission Aggregometry. The effect of the tested compounds on the platelet aggregation response induced by U-46619 was assessed as the percentage inhibition of the maximal platelet aggregation response. The IC_50_ values were determined using nonlinear sigmoidal regression analysis conducted with GraphPad Prism software. Under these experimental conditions, **50** and **51** caused a concentration-dependent inhibition of platelet aggregation with IC_50_ values of 26 μM [IC_50_ 95% CI: 22 to 30 μM] and 18 μM [IC_50_ 95% CI: 15 to 22 μM], respectively (Figure 7C).

### Ex Vivo Efficacy of **50** and **51** as TP Antagonists after Oral Dosing in Mice

We then tested the ability of compounds **50** and **51** to act as TP antagonists after in vivo administration in mice. To perform this test, based on preliminary PK and protein binding data, blood was drawn after one h, and PRP was prepared.^66^ PRP was challenged with increasing concentrations of U-46619 to trigger platelet aggregation. Both compounds were active after oral administration, causing a rightward shift in the concentration–response curve for U-46619 (Figure 8A–C) compared to the aggregation elicited by U-46619 in PRP obtained from untreated animals. Computer-assisted analysis calculated EC_50_ values for U-46619 to be 3.2 μM ± 2.3% CV, a value that shifted to 15 μM ± 5.3% CV and 4.7μM ± 4.3% CV upon treatment with compound **50** (200 mg/kg and 100 mg/kg, respectively; Figure 8 A,B), while compound **51** (50 mg/kg, Figure 8C) shifted the EC_50_ to 12.2 μM ± 5.4% CV. Based on these results, compound **51** proved capable of effectively suppressing TXA_2_-dependent platelet aggregation and appeared to be about fourfold more potent than **50** as a TP antagonist upon oral administration.

**Figure 8:**
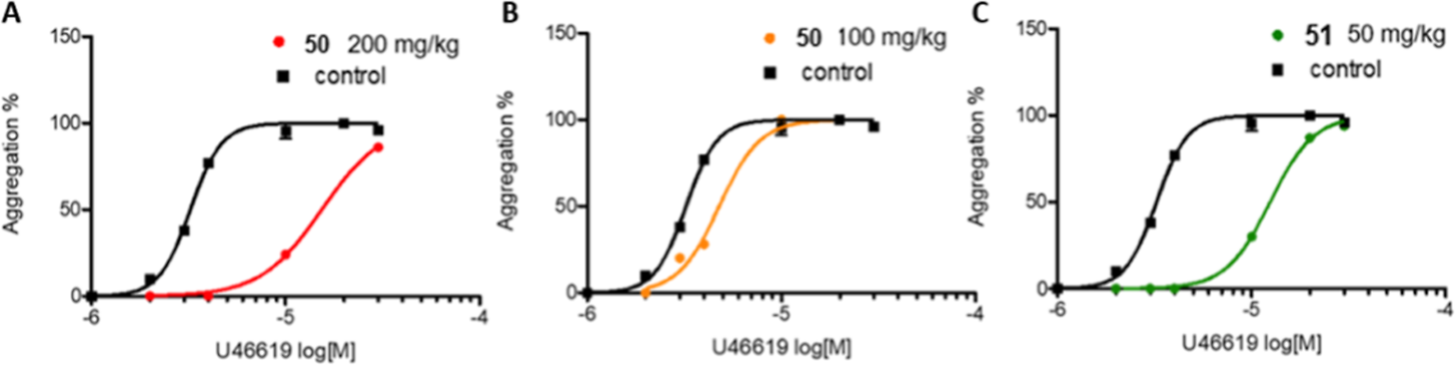
Ex vivo concentration–response curve for U-46619-triggered platelet aggregation after oral administration of compounds **50** and **51** in mice. One μM to 30 μM U-46619 was administered in the presence of either solvent (control, ■) or reported concentration of compound **50** (A and B, ●) or **51** (C, ●). A pool of animals was used, therefore each data point represents the mean of six different animals. Data were analyzed using the four parameters logistic model. All curves are computer-generated.

Overall, the analysis of the obtained results indicates that compound **51** shows the best profile as it can block COX-isozyme activity and acts as TP antagonist in a similar concentration range in vitro. In the whole blood assays in vitro, the ratio of COX-1/COX-2 IC_50_ was greater than 96. In the absence of plasma proteins, the COX-2 and TP antagonistic activities lie in the nanomolar range (IC_50_ values of 0.013 and 0.096 μM for COX-2 and TP, respectively). In human whole blood, compound **51** inhibits COX-2 with a potency similar to that of etodolac (3.4 μM, CI 95% = 1.4–9.8).^54^ As most of the acetic acid–based NSAIDs, **51** has a high degree of plasma protein binding (99.6% vs 99.5% for **1**), therefore its activities are reduced by more than 100-fold when measured in the presence of plasma proteins (IC_50_ values of 15 μM and 18 μM for COX-2 and TP, respectively). Both activities show a narrow concentration range, indicating balanced effect as a COX-2 inhibitor and TP receptor antagonist.

### Pharmacokinetics of 51 in Mice

We then decided to evaluate the preliminary PK properties of **51** in comparison with etodolac (**1**) after oral administration in mice at the cassette dose of 10 mg/kg each. Etodolac and **51** were administered to male CD1 mice suspended in saline and 20% cyclodextrin as the adjuvant by oral gavage. The PK parameters, *C*_max_, *t*_1/2_, *C*_24h_, AUC_0–24_, AUC_0–∞_, Cl/f, Vz/f were determined using noncompartmental pharmacokinetic analysis and are reported in Table 5. The oral exposure of compound **51** was significantly lower compared to **1** (etodolac), which is reflected in lower *C*_max_, *t*_1/2_, *C*_24h_, and AUC_0–24_, while the volume of distribution was higher.

**Table 5:** Determination of PK Parameters for **51** and Etodolac (**1**)­^a^

compound	MW (Da)	*C*_max_ ± CV % (ng/mL)	*C*_24h_ ± CV % (ng/mL)	*t*_1/2_ (h)	AUC_0–24_ (ng·h/mL)	AUC_0–∞_ (ng·h/mL)	*V*_z/f_ (mL/kg)	CL/f (mL/(h·kg)
**1**	287.15	10,089 ± 42.7%	4636 ± 12.4%	20.6	171,108	308,745	962	32.4
**51**	367.16	3170 ± 58.6%	819 ± 76.7%	11.6	45,638	59,347	2821	169

a Compounds were administered to male CD1 mice (*n* = 3) via oral gavage using saline and 20% cyclodextrin (200 μL) as the vehicle. Blood was retrieved from retrobulbar venous plexus at 1, 4 and 24 h. Plasma samples were analyzed by HPLC-MS with lower limit of detection of 2.4 ng/mL. All mice showed a normal behavior and there were no clinical signs observed after dosing. Data were analyzed with the software kinetica 5.0.

### Compound **51** Reduces Inflammatory Pain in Mice

To investigate the antinociceptive effects of **51** in vivo, we used a murine model of inflammatory pain induced by intraplantar injection of Complete Freund’s Adjuvant (CFA) into a hindpaw, which induces a paw edema and hypersensitivity to mechanical stimuli.^67,68^ Compound **51** (50 mg/kg), diclofenac, a well-known anti-inflammatory agent used for its analgesic properties (50 mg/kg) or vehicle were administered by oral gavage 24 h after the CFA injection and the mice were evaluated for mechanical hypersensitivity by testing the latency to paw withdrawal using a dynamic plantar aesthesiometer. As shown in Figure 9, the CFA injection evoked mechanical hypersensitivity, as indicated by a drop of paw withdrawal latencies 24 h post-CFA. Of note, after administration of **51**, paw withdrawal latencies were significantly increased compared to 24 h post-CFA over a period of 2 h, indicating an antinociceptive effect. Paw withdrawal latencies were also significantly increased 1 h after administration of diclofenac, which was used as a positive control. These data suggest that persisting inflammatory pain can be ameliorated by **51** in vivo.

**Figure 9:**
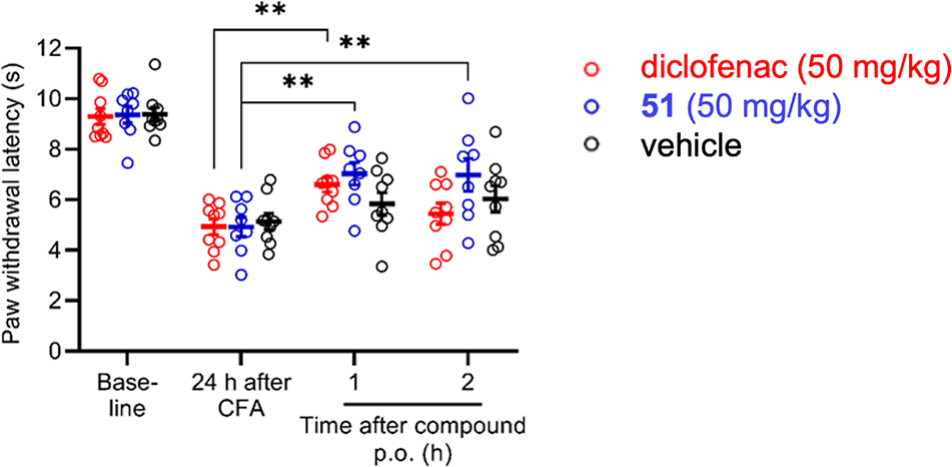
Antinociceptive effects of **51** in the CFA-induced inflammatory pain model in mice. Mechanical sensitivity of a hindpaw was determined using a dynamic plantar aesthesiometer. Twenty four h after CFA injection into a hindpaw mice were orally treated with **51** (50 mg/kg), diclofenac (50 mg/kg) or vehicle (3% Methocel in PBS). Note that paw withdrawal latencies were significantly increased 1 and 2 h after administration of **51** and 1 h after administration of diclofenac (*n* = 8–9 mice per group). Results are presented as the mean ± SEM. Data were analyzed by two-way repeated measures ANOVA with Dunnett’s post hoc test; **p < 0.005 compared to 24 h post-CFA.

## Conclusions

The development of new, safer anti-inflammatory drugs is a task long sought after by both industry and academia. Current use of either tNSAIDs or coxibs is associated with a variety of side effects, such as gastrointestinal, renal and cardiovascular effects. Recent evidence indicates that the activation of the TP receptor, exerted not only by TXA_2_ but also by other enzymatically and nonenzymatically formed eicosanoids, contributes to cardiovascular side effects of NSAIDs when prostacyclin is inhibited.^69^ The design of a new class of multitarget drugs able to act as dual COXi/TP antagonists, namely COXTRANs (COX inhibitors ThRomboxane ANtagonists), might represent a tool to solve the above-described problem. This approach offers several benefits over single-target inhibitors, as evidenced by other types of drugs,^70^ including the potential to attain higher efficacy and reduce adverse effects through lower drug dosages during treatment. Further research is necessary to substantiate this potential with our compound. In this work, we identified the 2-(1,3,4,9-tetrahydropyrano­[3,4-*b*]­indol-1-yl)­acetic acid scaffold, proper of etodolac, as the starting point for the design of dual COX inhibitors/TP antagonists. A series of 50 compounds was synthesized allowing the establishment of SAR for dual inhibition. Through a series of chemical modulations, we selected compound **51** as a potent and well-balanced COX-2 inhibitor/TP antagonist in vitro with a suitable PK for in vivo studies. Finally, we demonstrated that both the activities are maintained in vivo, with compound **51** being able to suppress TXA2-dependent platelet aggregation and inflammatory pain in mice after oral administration at 50 mg/kg. The possible safer cardiovascular profile of COXTRANs versus coxibs and the effects exerted by chronic administration of compound **51** should be verified in animal models of vascular injury.^71,72^ Undoubtedly, other applications of these drugs can be envisaged, as COX-2 and platelets-derived TXA_2_ can be implicated in tissue fibrosis, inflammation, cancer development, and metastasis.^73,74^

Moreover, the development of new COXTRANs is of great interest in many different therapeutic fields. COXTRANs with different selectivity on COX isoforms could be explored as antimetastatic agents. Indeed, the antimetastatic effect of agents capable of inhibiting the COX-1/TXA_2_ axis and platelet activation has been demonstrated, shedding light on a relevant implication of TXA_2_ in epithelial to mesenchymal transition, formation of the metastatic niche and on the generation of a pro-metastatic microenvironment.^75,76^ Importantly, recent evidence has shown that platelet-derived TXA_2_ through activation of TP receptor suppresses immunity by activating a T-cell intrinsic immunosuppressive pathway mediated by the guanine exchange factor ARHGEF1, thereby facilitating cancer metastasis.^77^ In addition, the role of COX-2 in the premetastatic niche formation has also been unveiled in several types of tumors, such as breast cancer, hepatocellular carcinoma and colorectal cancer.^73,78,79^ Further investigations on this class of compounds are therefore warranted.

## Experimental Section

### General Methods

All starting materials, applicable intermediates, reagents, and solvents were purchased from commercial suppliers and used as received. All the reactions were monitored by TLC on Merck 60 F_254_ (0.25 mm) plates, which were visualized by UV inspection and/or by spraying KMnO_4_ (0.5 g in 100 mL 0.1 N NaOH) or 2,4-dinitrophenylhydrazine (6 g in 60 mL of H_2_SO_4_, 40 mL of H_2_O and 200 mL of EtOH). Na_2_SO_4_ was used as drying agent for the organic phases. Flash chromatography purifications were performed using silica gel Fluka with 60 mesh particles. Dichloromethane was dried by refluxing over P_2_O_5_ under N_2_, distilled and stored over molecular sieves (4 Å). Dry tetrahydrofuran (THF) and dry diethyl ether were obtained by refluxing over Na (indicator: benzophenone) under N_2_. ^1^H and ^13^C NMR spectra were registered on Bruker AVANCE 300 spectrometer, at 300 and 75 MHz respectively, or on Jeol ECZ 600 M30 at 600 or 150 MHz, respectively. Chemical shifts (δ) are given in ppm, calibrated to tetramethylsilane (TMS) or to solvent signal as internal standard. The following abbreviations are used: s, singlet; d, doublet; t, triplet; q, quartet; dd, double doublet; m, multiplet. The following abbreviation is used to identify exact proton: ArH = Aromatic proton. ESI-mass spectra were recorded on a Waters Micromass Quattro Micro equipped with an ESI source. Purity of compounds was checked by RP-HPLC (HP1100, Agilent Technologies) equipped with UV–vis diode array detector using a LiChrosper 100 C18-e (250 × 4.6 mm, 5 μm, Merck) as analytical column and CH_3_CN/H_2_O + 0.1% TFA as solvent system with a flow rate of 1.0 mL/min. Detection was performed at λ = 226, 254, and 280 nm referenced against 800 nm. The analytical data confirmed that the purity of the products was ≥95%. Semipreparative HPLC purifications were carried out on a Varian Pro-Star 210 chromatograph equipped with a variable wavelength detector (Prostar 325). The chromatography was performed using a 5 μM particle size Hibar LiChrosper C18 end-capped prepacked column (250 × 25 mm), with a flow rate of 20 mL/min; UV detection was performed at 226 and 240 nm. The HRMS spectra were determined using a ZenoToF 7600 from Sciex (Framingham, MA) high-resolution mass spectrometer. Samples were infused at the concentration of 1 mg/L in methanol in the mass spectrometer operating in negative ESI mode with capillary voltage set to −4500 V and temperature set to 350 °C. Declustering potential was −80 ± 30 V and collision energy was 10 V. A full scan was performed from 100 to 1500 Da monitoring [M – H]^−^ ions. The following abbreviations were used: dichloromethane (DCM); petroleum ether (PE); ethyl acetate (EtOAc); methanol (MeOH); tetrahydrofuran (THF); dimethyl sulfoxide (DMSO); acetonitrile (CH_3_CN); dimethylacetamide (DMA); dimethylurea (DMU); dimethoxyethane (DME); *N*-methylpyrrolidone (NMP); trifluoroacetic acid (TFA), thin layer chromatography (TLC); nuclear magnetic resonance (NMR); mass spectrometry (MS); high performance liquid chromatography (HPLC), room temperature (RT), high resolution mass spectrometry (HRMS).

#### General Procedure for the Preparation of Compounds **58**–**61**

The appropriate aniline **54–57** (1 equiv) was suspended in HCl 37% (10 mL), and the mixture was cooled to 0 °C and stirred for 30 min. A solution of NaNO_2_ (1–2 equiv) in 3 mL of H_2_O was added dropwise and the obtained suspension was stirred at 0 °C for other 30 min. The mixture was then added to a suspension of SnCl_2_·2H_2_O (3 equiv) in HCl 37% (5 mL) at 0 °C. The mixture was vigorously stirred for 2 h, then it was filtered under vacuum. The collected solid was dried in a desiccator over P_2_O_5_ overnight and used directly in the following step without further purification.

##### (4-(Methylthio)­phenyl)­hydrazine Hydrochloride (**58**)

The reaction was run with **54** (1.5 g, 10 mmol), HCl 37% (24 + 6 mL), NaNO_2_ (0.78 g, 11 mmol), SnCl_2_·2H_2_O (6.77 g, 30 mmol). Product **58** was obtained as a gray solid.

##### (4-Fluoro-2-(methylthio) phenyl)­hydrazine Hydrochloride (**59**)

The reaction was run with **55** (2.01 g, 12.8 mmol), HCl 37% (11 + 3 mL), NaNO_2_ (1.06 g, 15.3 mmol), SnCl_2_·2H_2_O (8.66 g, 38.4 mmol). Product **59** was obtained as a gray solid.

##### (2-(Methylthio) phenyl)­hydrazine Hydrochloride (**60**)

The reaction was run with **56** (1 g, 7 mmol), HCl 37% (6 + 3 mL), NaNO_2_ (0.89 g, 12.9 mmol), SnCl_2_·2H_2_O (4.73 g, 21 mmol). Product **60** was obtained as a gray solid.

##### (4-Fluoro-2-iodophenyl)­hydrazine Hydrochloride (**61**)

The reaction was run with **57** (3g, 12.6 mmol), HCl 37% (10 + 3 mL), NaNO_2_ (1.74 g, 25.2 mmol), SnCl_2_·2H_2_O (8.52 g, 37.8 mmol). Product **61** was obtained as a gray solid.

##### 2-(5-(Methylthio)-1*H*-indol-3-yl)­ethan-1-ol (**64**)

To a stirred suspension of **58** (0.6 g, 3.15 mmol) in 4% H_2_SO_4_/DMA (14 mL, 1:1), 2,3-dihydrofuran (0.27 g, 0.29 mL, 3.85 mmol) was added dropwise. The mixture was heated at 55 °C for 6 h, then cooled to RT and NaOH 10% was added until pH 10. The mixture was stirred for 5 min and then extracted with EtOAc (3 × 20 mL). The combined organic phases were washed with brine (20 mL), dried over Na_2_SO_4_ and concentrated under reduced pressure. The crude product was purified by silica gel chromatography (DCM/MeOH 99:1) to give **64** (61 mg, 9.3% yield) as a blue-green oil. ^1^H NMR (600 MHz, CDCl_3_): δ 8.14 (s, 1H, NH), 7.60 (d, *J* = 1.5 Hz, 1H, ArH_7_), 7.29–7.24 (m, 1H, ArH_4_), 7.22 (dd, *J* = 8.5 Hz, 1.7 Hz, 1H, ArH_6_), 7.03 (d, *J* = 5.0 Hz, 1H, ArH_2_), 3.88 (t, *J* = 6.4 Hz, 2H, CH_2_OH), 2.99 (t, *J* = 6.4 Hz, 2H, CH_2_), 2.51 (s, 3H, SCH_3_), 1.99 (s, 1H, OH). ^13^C NMR (151 MHz, CDCl_3_): δ 171.44, 135.26, 128.27, 127.63, 124.28, 123.41, 119.79, 111.79, 62.63, 28.64, 19.00. MS (ESI^+^) *m*/*z*: 208 [M + H]^+^.

#### General Procedure for the Preparation of Compounds **65**–**66**–**68**–**69**

In a two-necked-round-bottom flask a mixture of tartaric acid/dimethylurea 40:60 was heated at 70 °C until it melted. The hydrazine **59**, **60**, **62**, **63** (1 equiv) and 2,3-dihydrofuran (2 equiv) were added to the mixture and stirred overnight under a nitrogen atmosphere. The reaction was then quenched by addition of NaOH 10% (20 mL) to the hot mixture. The mixture was extracted when still hot (40 °C) with DCM (3 × 20 mL), the extracts were washed with brine, dried over Na_2_SO_4_ and evaporated. The crude product was purified by flash column chromatography to afford the corresponding indole derivative.

##### 2-(5-Fluoro-7-(methylthio)-1*H*-indol-3-yl)­ethan-1-ol (**65**)

The reaction was run with **59** (2.67 g, 12.8 mmol), 2,3-dihydrofuran (1.94 mL, 25.6 mmol), Tartaric acid/DMU 40:60 (24 g). After purification by flash column chromatography (silica gel, DCM/MeOH 98:2) the product **65** was obtained as a red solid (1.40 g, 49% overall yield). ^1^H NMR (600 MHz, CDCl_3_): δ 8.31 (s, 1H, NH), 7.16–7.10 (m, 2H, ArH_6–2_), 6.97 (dd, *J* = 9.5, 2.2 Hz, 1H, ArH_4_), 3.87 (t, *J* = 6.4 Hz, 2H, CH_2_OH), 2.96 (t, *J* = 6.3 Hz, 2H, CH_2_), 2.51 (s, 3H, SCH_3_), 1.96 (s, 1H, OH). ^13^C NMR (151 MHz, CDCl_3_): δ 157.95 (d, *J*_C–F_ = 236.8 Hz), 132.66, 127.27 (d, *J*_C–F_ = 10.0 Hz), 124.25, 120.73 (d, *J*_C–F_ = 9.9 Hz), 113.44 (d, *J*_C–F_ = 5.0 Hz), 111.11 (d, *J*_C–F_ = 27.8 Hz), 102.57 (d, *J*_C–F_ = 23.5 Hz), 62.61, 28.80, 17.30. MS (ESI^–^) *m*/*z*: 224 [M – H]^−^.

##### 2-(7-(Methylthio)-1*H*-indol-3-yl)­ethan-1-ol (**66**)

The reaction was run with **60** (0.4 g, 2.1 mmol), 2,3-dihydrofuran (0.32 mL, 4.2 mmol), tartaric acid/DMU 40:60 (4 g). After purification by flash column chromatography (silica gel, DCM/MeOH 98:2) the product **66** was obtained as a red solid (87 mg, 20% overall yield). ^1^H NMR (600 MHz, CDCl_3_): δ 8.61 (s, 1H, NH), 7.55 (d, *J* = 7.9 Hz, 1H, ArH_4_), 7.29 (d, *J* = 7.3 Hz, 1H, ArH_6_), 7.14 (t, *J* = 7.6 Hz, 1H, ArH_5_), 7.03 (d, *J* = 1.7 Hz, 1H, ArH_2_), 3.91 (t, *J* = 6.5 Hz, 2H, CH_2_OH), 3.03 (t, *J* = 6.5 Hz, 2H, CH_2_), 2.55–2.47 (m, 3H, SCH_3_), 2.22 (s, 1H, OH). ^13^C NMR (151 MHz, CDCl_3_): δ 136.60, 127.48, 124.06, 122.92, 120.23, 119.24, 118.05, 113.16, 62.75, 28.93, 17.91. MS (ESI^–^) *m*/*z*: 206 [M – H]^−^.

##### 2-(7-Chloro-5-fluoro-1*H*-indol-3-yl)­ethan-1-ol (**68**)

The reaction was run with (2-chloro-4-fluorophenyl)­hydrazine hydrochloride (**62**, 1 g, 5.1 mmol), 2,3-dihydrofuran (0.77 mL, 10.2 mmol), tartaric acid/DMU 40:60 (9.7 g). After purification by flash column chromatography (silica gel, PE/EtOAc/MeOH 8:1.5:0.5) the product **68** was obtained as an orange solid (232 mg, 21% yield). ^1^H NMR (300 MHz, CDCl_3_): δ 8.50 (s, 1H, NH), 7.08 (m, 1H, ArH_4_), 7.04 (s, 1H, ArH_2_), 6.91 (m, 1H, ArH_6_), 3.78 (t, *J* = 6.4 Hz, 2H, CH_2_OH), 2.86 (t, *J* = 6.4 Hz, 2H, CH_2_), 2.31 (s, 1H, OH). ^13^C NMR (75 MHz, CDCl_3_): δ 157.46 (d, *J*_C–F_ = 235 Hz), 130.83, 128.78 (d, *J*_C–F_ = 10.5 Hz), 125.29, 116.95 (d, *J*_C–F_ = 12.8 Hz), 114.14 (d, *J*_C–F_ = 2 Hz), 110.79 (d, *J*_C–F_ = 30 Hz), 103.14 (d, *J*_C–F_ = 2.3 Hz), 62.85, 28.96. MS (ESI^+^) *m*/*z*: 236/238 [M + Na]^+^.

##### 2-(7-Chloro-5-methyl-1*H*-indol-3-yl)­ethan-1-ol (**69**)

The reaction was run with (2-chloro-4-methylphenyl)­hydrazine hydrochloride (**63**, 1.5 g, 7.8 mmol), 2,3-dihydrofuran (1.18 mL, 15.6 mmol), tartaric acid/DMU 40:60 (14.8 g). After purification by flash column chromatography (silica gel, DCM/MeOH 98:2) the product **69** was obtained as a yellow solid (380 mg, 23% yield). ^1^H NMR (300 MHz, CDCl_3_): δ 8.55 (s, 1H, NH), 7.08 (d, *J* = 1.4 Hz, 1H, ArH_4_), 7.04 (s, 1H, ArH_2_), 6.91 (d, *J* = 1.4 Hz, 1H, ArH_6_), 3.78 (t, *J* = 6.4 Hz, 2H, CH_2_OH), 2.86 (t, *J* = 6.4 Hz, 2H, CH_2_), 2,49 (s, 3H, CH_3_), 2.31 (s, 1H, OH). ^13^C NMR (75 MHz, CDCl_3_): δ 157.46 (d, *J*_C–F_ = 235 Hz), 130.83, 128.78 (d, *J*_C–F_ = 10.5 Hz), 125.29, 116.95 (d, *J*_C–F_ = 12.8 Hz), 114.14 (d, *J*_C–F_ = 2 Hz), 110.79 (d, *J*_C–F_ = 30 Hz), 103.14 (d, *J*_C–F_ = 2.3 Hz), 62.85, 28.96, 21.12. MS (ESI^–^) *m*/*z*: 208/210 [M – H]^−^.

##### 2-(5-Fluoro-7-iodo-1*H*-indol-3-yl)­ethan-1-ol (**67**)

To a solution of (4-fluoro-2-iodophenyl)­hydrazine hydrochloride (**61**, 3.63 g, 12.6 mmol, 1 equiv) in 1,4-dioxane/H_2_O (20 mL/1.20 mL), 2,3-dihydrofuran (1.42 mL, 18.8 mmol, 1.5 equiv) was added and the mixture was stirred at 95 °C under N_2_ atmosphere for 4 h. The reaction was cooled to RT and concentrated in vacuo. The obtained solid residue was resuspended in DCM (15 mL) and H_2_O (15 mL), and the suspension was filtered under vacuum. The filtrate was extracted with DCM (3 × 20 mL), the combined organic layers were washed with brine (30 mL), dried over Na_2_SO_4_ and evaporated to dryness. The crude product was purified by flash column chromatography (silica gel, PE/EtOAc/MeOH 8:1.5:0.5) to afford **67** as a yellow solid (1.35 g, 35% yield). ^1^H NMR (600 MHz, CDCl_3_): δ 8.12 (s, 1H, NH), 7.35 (dd, *J* = 8.48, 2.28 Hz, 1H, ArH_6_), 7.31–7.22 (m, 1H, ArH_4_), 7.20 (d, *J* = 2.39 Hz, 1H, ArH_2_), 3.89 (m, 2H, CH_2_OH), 2.95 (td, *J* = 6.34, 0.69 Hz, 2H, CH_2_), 1.44 (t, *J* = 5.80 Hz, 1H, OH). ^13^C NMR (151 MHz, CDCl_3_): δ 157.24 (d, *J*_C–F_ = 239.4 Hz), 127.22 (d, *J*_C–F_ = 9.9 Hz), 119.17, 114.25 (d, *J*_C–F_ = 4.9 Hz), 113.45 (d, *J*_C–F_ = 29.1 Hz), 104.32 (d, *J*_C–F_ = 12.3 Hz), 103.53 (d, *J*_C–F_ = 23.2 Hz), 75.49, 62.55, 28.95. MS (ESI^–^) *m*/*z*: 304 [M – H]^−^.

#### General Procedure for the Preparation of Compounds **76**–**91**

In a flame-dried two-necked-round-bottom flask, kept under N_2_ atmosphere, a solution of the appropriate 2-indolethanol **64**–**72** (1 equiv) in anhydrous DCM was added via syringe. The appropriate β-ketoester **73**–**75** (1.2 equiv) was added via a syringe and the mixture was cooled to 0 °C under stirring. BF_3_OEt_2_ (0.8 equiv) was added dropwise using a syringe, then the reaction was stirred at RT until completion (5h). The reaction was quenched with saturated aqueous NaHCO_3_ solution and extracted with DCM (3 × 30 mL). The combined organic layers were washed with brine, dried over Na_2_SO_4_ and concentrated in vacuo. The crude product was purified by flash column chromatography to afford the corresponding product **76–91**.

##### Methyl 2-(1-Methyl-6-(methylthio)-1,3,4,9-tetrahydropyrano­[3,4-*b*]­indol-1-yl)­acetate (**76**)

The reaction was run with **64** (0.2 g, 0.96 mmol), methyl acetoacetate (**73**, 0.12 mL, 1.15 mmol), BF_3_OEt_2_ (95 μL, 0.77 mmol), DCM (5 mL). After purification by flash column chromatography (silica gel, PE/EtOAc 9:1) the product **76** was obtained as a yellow solid (211 mg, 72% yield). ^1^H NMR (600 MHz, CDCl_3_): δ 9.13 (s, 1H, NH), 7.52 (d, *J* = 1.7 Hz, 1H, ArH_8_), 7.29 (d, *J* = 8.3 Hz, 1H, ArH_5_), 7.21 (dd, *J* = 8.4, 1.9 Hz, 1H, ArH_7_), 4.01 (td, *J* = 5.4, 2.6 Hz, 2H, OCH_2_−), 3.74 (s, 3H, COOCH_3_), 3.01 and 2.89 (two signals, d, *J* = 16.5 Hz, 2H, CH_2_COO−), 2.77 (td, *J* = 5.2, 2.0 Hz, 2H, CH_2_), 2.49 (s, 3H, SCH_3_), 1.66 (s, 3H, CH_3_). MS (ESI^+^) *m*/*z*: 306 [M + H]^+^.

##### Methyl 2-(1-Ethyl-6-fluoro-8-(methylthio)-1,3,4,9-tetrahydropyrano­[3,4-*b*]­indol-1-yl)­acetate (**77**)

The reaction was run with **65** (0.5 g, 2.22 mmol), methyl propionylacetate (**74**, 0.33 mL, 2.66 mmol), BF_3_OEt_2_ (0.21 mL, 1.77 mmol), DCM (20 mL). After purification by flash column chromatography (silica gel, PE/EtOAc 9:1) the product **77** was obtained as a gray solid (674 mg, 90% yield). ^1^H NMR (600 MHz, CDCl_3_): δ 9.24 (s, 1H, NH), 7.01 (dd, *J* = 9.2, 2.2 Hz, 1H, ArH_7_), 6.92 (dd, *J* = 9.6, 2.3 Hz, 1H, ArH_5_), 4.07–3.88 (m, 2H, OCH_2_−), 3.73 (s, 3H, COOCH_3_), 3.01 and 2.95 (two signals, d, *J* = 16.4 Hz, 2H, CH_2_COO−), 2.80–2.63 (m, 2H, CH_2_), 2.57–2.50 (m, 3H, SCH_3_), 2.06 (m, 2H, C*H*_2_CH_3_), 0.82 (t, *J* = 7.4 Hz, 3H, CH_3_). ^13^C NMR (151 MHz, CDCl_3_): δ 172.98, 157.91 (d, *J*_C–F_ = 235.9 Hz), 138.33, 131.80, 126.17 (d, *J*_C–F_ = 10.1 Hz), 120.54, 110.24 (d, *J*_C–F_ = 27.5 Hz), 109.00 (d, *J*_C–F_ = 4.7 Hz), 101.91 (d, *J*_C–F_ = 23.3 Hz), 74.68, 60.55, 52.22, 42.73, 30.63, 22.38, 17.06, 7.63. MS (ESI^–^) *m*/*z*: 336 [M – H]^−^.

##### Methyl 2-(1-Methyl-8-(methylthio)-1,3,4,9-tetrahydropyrano­[3,4-*b*]­indol-1-yl)­acetate (**78**)

The reaction was run with **66** (0.2 g, 0.96 mmol), methyl acetoacetate (**73**, 0.12 mL, 1.15 mmol), BF_3_OEt_2_ (95 μL, 0.77 mmol), DCM (20 mL). After purification by flash column chromatography (silica gel, PE/EtOAc 9:1) the product **78** was obtained as a yellow solid (208 mg, 71% yield). ^1^H NMR (600 MHz, CDCl_3_): δ 9.28 (s, 1H, NH), 7.40 (d, *J* = 7.8 Hz, 1H, ArH_5_), 7.25–7.21 (m, 1H, ArH_7_), 7.09–7.06 (m, 1H, ArH_6_), 4.08–3.97 (m, 2H, OCH_2_−), 3.74 (s, 3H, COOCH_3_), 3.02 and 2.91 (two signals, d, *J* = 16.2 Hz, 2H, CH_2_COO−), 2.84–2.77 (m, 2H, CH_2_), 2.52 (s, 3H, SCH_3_), 1.70 (s, 3H, CH_3_). ^13^C NMR (151 MHz, CDCl_3_): δ 172.79, 137.32, 135.82, 126.37, 123.84, 120.14, 119.08, 117.43, 107.80, 72.38, 60.63, 52.17, 45.54, 25.51, 22.48, 17.78. MS (ESI^–^) *m*/*z*: 304 [M – H]^−^.

##### Methyl 2-(1-Ethyl-8-(methylthio)-1,3,4,9-tetrahydropyrano­[3,4-*b*]­indol-1-yl)­acetate (**79**)

The reaction was run with **66** (0.463 g, 2.23 mmol), methyl propionylacetate (**74**, 0.34 mL, 2.68 mmol), BF_3_OEt_2_ (0.22 mL, 1.78 mmol), DCM (20 mL). After purification by flash column chromatography (silica gel, PE/EtOAc 9:1) the product **79** was obtained as a gray solid (641 mg, 90% yield). MS (ESI^–^) *m*/*z*: 318 [M – H]^−^.

##### Methyl 2-(1-Ethyl-6-fluoro-8-iodo-1,3,4,9-tetrahydropyrano­[3,4-*b*]­indol-1-yl)­acetate (**80**)

The reaction was run with **67** (1 g, 3.27 mmol), methyl propionylacetate (**74**, 0.49 mL, 3.93 mmol), BF_3_OEt_2_ (0.4 mL, 2.62 mmol), DCM (20 mL). After purification by flash column chromatography (silica gel, PE/EtOAc 9:1) the product **80** was obtained as an off-white solid (1.02 g, 75% yield). ^1^H NMR (600 MHz, CDCl_3_): δ 9.22 (s, 1H, NH), 7.29 (dd, *J* = 8.6, 2.2 Hz, 1H, ArH_7_), 7.13 (dd, *J* = 9.1, 2.2 Hz, 1H, ArH_5_), 4.03–3.92 (m, 2H, OCH_2_−), 3.76 (s, 3H, COOCH_3_), 3.01 and 2.93 (two signals, d, *J* = 16.6 Hz, 2H, CH_2_COO−), 2.76–2.69 (m, *J* = 15.2, 4.7 Hz, 2H, CH_2_), 2.15–2.01 (m, *J* = 14.7, 7.3 Hz, 2H, C*H*_2_CH_3_), 0.84 (t, *J* = 7.4 Hz, 3H, CH_3_). ^13^C NMR (151 MHz, CDCl_3_): δ 173.05, 157.20 (d, *J*_C–F_ = 238.7 Hz), 139.06, 134.84, 126.02 (d, *J*_C–F_ = 10.0 Hz), 118.43 (d, *J*_C–F_ = 23.3 Hz), 109.65 (d, *J*_C–F_ = 4.9 Hz), 103.67 (d, *J*_C–F_ = 26.2 Hz), 75.37 (d, *J*_C–F_ = 8.7 Hz), 74.63, 60.43, 52.31, 42.76, 30.64, 22.47, 7.64. MS (ESI^–^) *m*/*z*: 416 [M – H]^−^.

##### Methyl 2-(8-Chloro-1-ethyl-6-fluoro-1,3,4,9-tetrahydropyrano­[3,4-*b*]­indol-1-yl)­acetate (**81**)

The reaction was run with **68** (0.2 g, 0.94 mmol), methyl propionylacetate (**74**, 0.14 mL, 1.13 mmol), BF_3_OEt_2_ (0.09 mL, 0.75 mmol), DCM (10 mL). After purification by flash column chromatography (silica gel, PE/EtOAc 9:1) the product **81** was obtained as a white solid (303 mg, 99% yield). ^1^H NMR (300 MHz, CDCl_3_): δ 9.32 (s, 1H, NH), 7.07 (dd, *J* = 9.0, 2.2 Hz, 1H, ArH_7_), 6.97 (dd, *J* = 9.1, 2.2 Hz, 1H, ArH_5_), 4.09–3.86 (m, 2H, OCH_2_−), 3.75 (s, 3H, COOCH_3_), 3.02 and 2.92 (two signals, d, *J* = 16.6 Hz, 2H, CH_2_COO−), 2.83–2.63 (m, 2H, CH_2_), 2.08 (qt, *J* = 22.0, 7.3 Hz, 2H, C*H*_2_CH_3_), 0.83 (t, *J* = 7.4 Hz, 3H, CH_3_). ^13^C NMR (75 MHz, CDCl_3_): δ 173.37, 157.50 (d, *J*_C–F_ = 235.5 Hz), 139.38, 130.15, 127.72 (d, *J*_C–F_ = 9.75 Hz), 116.88 (d, *J*_C–F_ = 12.8 Hz), 110. Twenty-eight (d, *J*_C–F_ = 29.25 Hz), 109.77 (d, *J*_C–F_ = 4.5 Hz), 102.61 (d, *J*_C–F_ = 23.25 Hz), 74.95, 60.79, 52.58, 42.93, 30.84, 22.69, 7.90. MS (ESI^+^) *m*/*z*: 328/326 [M + H]^+^.

##### Methyl 2-(8-Chloro-6-fluoro-1-methyl-1,3,4,9-tetrahydropyrano­[3,4-*b*]­indol-1-yl)­acetate (**82**)

The reaction was run with **68** (0.5 g, 2.3 mmol), methyl acetoacetate (**73**, 0.3 mL, 2.8 mmol), BF_3_OEt_2_ (0.23 mL, 1.84 mmol), DCM (15 mL). After purification by flash column chromatography (silica gel, PE/EtOAc 9:1) the product **82** was obtained as a white solid (645 mg, 90% yield). ^1^H NMR (300 MHz, CDCl_3_): δ 9.36 (s, 1H, NH), 7.06 (dd, *J* = 8.9, 2.0 Hz, 1H, ArH_7_), 6.98 (dd, *J* = 9.1, 2.3 Hz, 1H, ArH_5_), 4.02 (t, *J* = 5.5 Hz, 2H, OCH_2_−), 3.76 (s, 3H, COOCH_3_), 3.02 and 2.90 (two signals, d, *J* = 16.5 Hz, 2H, CH_2_COO−), 2.77–2.71 (m, 2H, CH_2_), 1.70 (s, 3H, CH_3_). ^13^C NMR (75 MHz, CDCl_3_): δ 173.30, 157.52 (d, *J*_C–F_ = 235.5 Hz), 140.00, 130.10, 127.75 (d, *J*_C–F_ = 10.5 Hz), 116.92 (d, *J*_C–F_ = 13.3 Hz), 110.37 (d, *J*_C–F_ = 29.2 Hz), 108.72 (d, *J*_C–F_ = 5.2 Hz), 102.68 (d, *J*_C–F_ = 23.25 Hz), 72.59, 60.78, 52.60, 45.71, 25.64, 22.68. MS (ESI^+^) *m*/*z*: 334/336 [M + Na]^+^.

##### Methyl 2-(1-Benzyl-8-chloro-6-fluoro-1,3,4,9-tetrahydropyrano­[3,4-*b*]­indol-1-yl)­acetate (**83**)

The reaction was run with **68** (0.234 g, 1.09 mmol), methyl 3-oxo-4-phenylbutyrate (**75**, 0.23 mL, 1.31 mmol), BF_3_OEt_2_ (0.11 mL, 0.87 mmol), DCM (10 mL). After purification by flash column chromatography (silica gel, PE/EtOAc 95:5) the product **83** was obtained as a white solid (271 mg, 64% yield). ^1^H NMR (600 MHz, CDCl_3_): δ 9.47 (s, 1H, NH), 7.30–7.22 (m, 3H, ArH_2′‑4′‑6′_), 7.21–7.16 (m, 2H, ArH_3′–5′_), 7.06 (dd, *J* = 9.0, 2.2 Hz, 1H, ArH_7_), 6.99 (dd, *J* = 9.0, 2.2 Hz, 1H, ArH_5_), 4.14–3.99 (m, 2H, OCH_2_−), 3.72 (s, 3H, COOCH_3_), 3.43 and 3.37 (two signals, d, *J* = 14.1 Hz, 2H, CH_2_COO−), 2.87–2.73 (m, 2H, CH_2_), 2.72–2.62 (m, 2H, CH_2_Ph). ^13^C NMR (151 MHz, CDCl_3_): δ 173.29, 157.21 (d, *J*_C–F_ = 237.3 Hz), 139.01, 136.17, 130.56, 129.77, 128.19, 127.34 (d, *J*_C–F_ = 10.2 Hz), 126.92, 116.64 (d, *J*_C–F_ = 12.9 Hz), 110.16 (d, *J*_C–F_ = 29.6 Hz), 109.48 (d, *J*_C–F_ = 4.5 Hz), 102.38 (d, *J*_C–F_ = 23.3 Hz), 74.72, 60.28, 52.23, 42.52, 42.39, 22.33. MS (ESI^+^) *m*/*z*: 388/390 [M + H]^+^.

##### Methyl 2-(8-Chloro-1-ethyl-6-methyl-1,3,4,9-tetrahydropyrano­[3,4-*b*]­indol-1-yl)­acetate (**84**)

The reaction was run with **69** (0.38 g, 1.81 mmol), methyl propionylacetate (**74**, 0.27 mL, 2.17 mmol), BF_3_OEt_2_ (0.18 mL, 1.45 mmol), DCM (10 mL). After purification by flash column chromatography (silica gel, PE/EtOAc 9:1) the product **84** was obtained as a yellow solid (408 mg, 70% yield). ^1^H NMR (600 MHz, CDCl_3_): δ 9.10 (s, 1H, NH), 7.18 (d, *J* = 0.5 Hz, 1H, ArH_7_), 7.01 (d, *J* = 0.7 Hz, 1H, ArH_5_), 4.08–3.88 (m, 2H, OCH_2_−), 3.73 (s, 3H, COOCH_3_), 3.02 and 2.93 (two signals, d, *J* = 16.5 Hz, 2H, CH_2_COO−), 2.83–2.70 (m, 2H, CH_2_), 2.43 (s, 3H, ArCH_3_), 2.19–2.00 (m, 2H, C*H*_2_CH_3_), 0.84 (t, *J* = 7.4 Hz, 3H, CH_3_). ^13^C NMR (151 MHz, CDCl_3_): δ 172.91, 137.26, 131.37, 129.88, 128.17, 122.61, 116.71, 116.28, 108.79, 74.76, 60.59, 52.17, 42.81, 30.70, 22.48, 21.35, 7.64. MS (ESI^+^) *m*/*z*: 322/324 [M + H]^+^.

##### Methyl 2-(8-Ethyl-1-methyl-1,3,4,9-tetrahydropyrano­[3,4-*b*]­indol-1-yl)­acetate (**85**)

The reaction was run with **70** (0.5 g, 2.6 mmol), methyl acetoacetate (**73**, 0.34 mL, 3.2 mmol), BF_3_OEt_2_ (0.26 mL, 2.08 mmol), DCM (15 mL). After purification by flash column chromatography (silica gel, PE/EtOAc 9:1) the product **85** was obtained as a white solid (687 mg, 92% yield). ^1^H NMR (300 MHz, CDCl_3_): δ 9.08 (s, 1H, NH), 7.31 (d, *J* = 7.6 Hz, 1H, ArH_6_), 7.04–6.93 (m, 2H, ArH_5–7_), 4.01–3.92 (m, 2H, OCH_2_−), 3.68 (s, 3H, COOCH_3_), 3.03–2.78 (m, 4H, CH_2_COO- and ArC*H*_2_CH_3_), 2.75 (t, *J* = 5.5 Hz, 2H, CH_2_), 1.65 (s, 3H, CH_3_), 1.32 (t, *J* = 7.6 Hz, 3H, ArCH_2_C*H*_3_). ^13^C NMR (75 MHz, CDCl_3_): δ 173.67, 137.06, 134.78, 127.06, 126.56, 120.92, 120.10, 116.44, 107.72, 72.65, 61.07, 52.47, 45.95, 25.86, 24.62, 22.83, 14.20. MS (ESI^+^) *m*/*z*: 288 [M + H]^+^.

##### Methyl 2-(1-Benzyl-8-ethyl-1,3,4,9-tetrahydropyrano­[3,4-*b*]­indol-1-yl)­acetate (**86**)

The reaction was run with **70** (0.5 g, 2.6 mmol), methyl 3-oxo-4-phenylbutyrate (**75**, 0.55 mL, 3.12 mmol), BF_3_OEt_2_ (0.26 mL, 2.08 mmol), DCM (15 mL). After purification by flash column chromatography (silica gel, PE/EtOAc 9:1) the product **86** was obtained as a white solid (756 mg, 80% yield). ^1^H NMR (300 MHz, CDCl_3_): δ 9.19 (s, 1H, NH), 7.41–6.99 (m, 8H, ArH_5,6,7_ and ArH_2′,3′,4′,5′‑6′_), 4.18–4.03 (m, 2H, OCH_2_−), 3.72 (s, 3H, COOCH_3_), 3.50–3.42 (m, 2H, CH_2_COO−), 2.91 (q, *J* = 7.3 Hz, 2H, ArC*H*_2_CH_3_), 2.86–2.64 (m, 4H, CH_2_Ph and CH_2_), 1.41 (t, *J* = 7.6 Hz, 3H, CH_2_C*H*_3_). ^13^C NMR (75 MHz, CDCl_3_): δ 173.4, 136.5, 135.9, 134.3, 130.5, 129.5, 128.9, 128.0, 126.6, 120.4, 119.6, 116.0, 108.3, 74.6, 60.4, 51.9, 42.6, 42.4, 24.2, 22.3, 13.8. MS (ESI^+^) *m*/*z*: 364 [M + H]^+^.

##### Methyl 2-(8-Chloro-1-methyl-1,3,4,9-tetrahydropyrano­[3,4-*b*]­indol-1-yl)­acetate (**87**)

The reaction was run with **71** (0.5 g, 2.6 mmol), methyl acetoacetate (**73**, 0.33 mL, 3.07 mmol), BF_3_OEt_2_ (0.26 mL, 2.08 mmol), DCM (15 mL). After purification by flash column chromatography (silica gel, PE/EtOAc 9:1) the product **87** was obtained as a waxy solid (489 mg, 64% yield). ^1^H NMR (300 MHz, CDCl_3_): δ 9.38 (s, 1H, NH), 7.41 (d, *J* = 7.7 Hz, 1H, ArH_7_), 7.18 (d, *J* = 7.1 Hz, 1H, ArH_5_) 7.04 (t, *J* = 7.7 Hz, 1H, ArH_6_), 4.04 (t, *J* = 5.5 Hz, 2H, OCH_2_−), 3.78 (s, 3H, COOCH_3_), 3.02 and 2.91 (two signals, d, *J* = 16.5 Hz, 2H, CH_2_COO−), 2.82 (t, *J* = 5.5 Hz, 2H, CH_2_), 1.74 (s, 3H, CH_3_). ^13^C NMR (75 MHz, CDCl_3_): δ 173.22, 138.22, 133.29, 128.38, 121.63, 120.53, 117.30, 117.13, 108.46, 72.65, 60.87, 52.56, 45.77, 25.73, 22.79. MS (ESI^+^) *m*/*z*: 294/296 [M + H]^+^.

##### Methyl 2-(8-Chloro-1-ethyl-1,3,4,9-tetrahydropyrano­[3,4-*b*]­indol-1-yl)­acetate (**88**)

The reaction was run with **71** (0.113 g, 0.57 mmol), methyl propionylacetate (**74**, 87 μL, 0.69 mmol), BF_3_OEt_2_ (57 μL, 0.46 mmol), DCM (10 mL). After purification by flash column chromatography (silica gel, PE/EtOAc 9:1) the product **88** was obtained as a white solid (118 mg, 67% yield). ^1^H NMR (600 MHz, CDCl_3_): δ 9.26 (s, 1H, NH), 7.39 (d, *J* = 7.9 Hz, 1H, ArH_7_), 7.16 (dd, *J* = 7.6, 0.6 Hz, 1H, ArH_5_), 7.02 (t, *J* = 7.7 Hz, 1H, ArH_6_), 4.07–3.93 (m, 2H, OCH_2_−), 3.74 (s, 3H, COOCH_3_), 3.02 and 2.94 (two signals, d, *J* = 16.5 Hz, 2H, CH_2_COO−), 2.86–2.73 (m, 2H, CH_2_), 2.21–1.97 (m, 2H, C*H*_2_CH_3_), 0.83 (t, *J* = 7.4 Hz, 3H, CH_3_). ^13^C NMR (151 MHz, CDCl_3_): δ 172.98, 137.19, 133.04, 128.04, 121.24, 120.14, 116.91, 116.80, 109.26, 74.70, 60.56, 52.21, 42.76, 30.64, 22.47, 7.64. MS (ESI^+^) *m*/*z*: 308/310 [M + H]^+^.

##### Methyl 2-(1-Benzyl-8-chloro-1,3,4,9-tetrahydropyrano­[3,4-*b*]­indol-1-yl)­acetate (**89**)

The reaction was run with **71** (0.28 g, 1.43 mmol), methyl 3-oxo-4-phenylbutyrate (**75**, 0.30 mL, 1.72 mmol), BF_3_OEt_2_ (0.14 mL, 1.14 mmol), DCM (10 mL). After purification by flash column chromatography (silica gel, PE/EtOAc 95:5) the product **89** was obtained as a white solid (349 mg, 66% yield). ^1^H NMR (600 MHz, CDCl_3_): δ 9.43 (s, 1H, NH), 7.40 (d, *J* = 7.8 Hz, 1H, ArH_7_), 7.30–7.17 (m, 6H, ArH_5–2′‑3′‑4′‑5′‑6′_), 7.04 (t, *J* = 7.7 Hz, 1H, ArH_6_), 4.17–4.02 (m, 2H, OCH_2_−), 3.72 (s, 3H, COOCH_3_), 3.40 (q, *J* = 14.4 Hz, 2H, CH_2_COO−), 2.88–2.67 (m, 4H, CH_2_Ph and CH_2_). ^13^C NMR (151 MHz, CDCl_3_): δ 173.22, 137.23, 136.34, 132.96, 130.60, 128.18, 127.98, 126.86, 121.39, 120.20, 117.01, 116.86, 109.22, 74.76, 60.35, 52.18, 42.59, 42.44, 22.43. MS (ESI^+^) *m*/*z*: 370/372 [M + H]^+^.

##### Methyl 2-(6-Fluoro-1-methyl-1,3,4,9-tetrahydropyrano­[3,4-*b*]­indol-1-yl)­acetate (**90**)

The reaction was run with **72** (1.4 g, 7.8 mmol), methyl acetoacetate (**73**, 1 mL, 9.4 mmol), BF_3_OEt_2_ (0.77 mL, 6.24 mmol), DCM (30 mL). After purification by flash column chromatography (silica gel, PE/EtOAc 9:1) the product **90** was obtained as a white solid (1.95 g, 90% yield). ^1^H NMR (300 MHz, CDCl_3_): δ 9.17 (s, 1H, NH), 7.27 (dd, *J* = 8.8, 4.4 Hz, 1H, ArH_7_), 7.15 (dd, *J* = 9.5, 2.4 Hz, 1H, ArH_8_), 6.92 (td, *J* = 9.1, 2.5 Hz, 1H, ArH_5_), 4.03 (t, *J* = 5.5 Hz, 2H, OCH_2_−), 3.75 (s, 3H, COOCH_3_), 3.04 and 2.92 (two signals, d, *J* = 16.5 Hz, 2H, CH_2_COO−), 2.78 (t, *J* = 5.5 Hz, 2H, CH_2_), 1.70 (s, 3H, CH_3_). ^13^C NMR (75 MHz, CDCl_3_): δ 173.29, 158.16 (d, *J*_C–F_ = 232.5 Hz), 139.36, 132.52, 127.23 (d, *J*_C–F_ = 9.7 Hz), 112.17 (d, *J*_C–F_ = 9 Hz), 110.51 (d, *J*_C–F_ = 25.5 Hz), 107.52 (d, *J*_C–F_ = 4.5 Hz), 103.67 (d, *J*_C–F_ = 23.25 Hz), 72.67, 60.88, 52.42, 45.63, 25.74, 22.61. MS (ESI^+^) *m*/*z*: 300 [M + Na]^+^.

##### Methyl 2-(1-Ethyl-6-fluoro-1,3,4,9-tetrahydropyrano­[3,4-*b*]­indol-1-yl)­acetate (**91**)

The reaction was run with **72** (0.200 g, 1.12 mmol), methyl propionylacetate (**74**, 169 μL, 1.34 mmol), BF_3_OEt_2_ (110 μL, 0.89 mmol), DCM (10 mL). After purification by flash column chromatography (silica gel, PE/EtOAc 9:1) the product **91** was obtained as a white solid (218 mg, 67% yield). ^1^H NMR (600 MHz, CDCl_3_): δ 9.07 (s, 1H, NH), 7.26 (dd, *J* = 8.8, 4.4 Hz, 1H, ArH_7_), 7.13 (dd, *J* = 9.5, 2.5 Hz, 1H, ArH_8_), 6.90 (ddd, *J* = 9.3, 8.7, 2.5 Hz, 1H, ArH_5_), 4.07–3.91 (m, 2H, OCH_2_−), 3.73 (s, 3H, COOCH_3_), 3.01 and 2.91 (two signals, d, *J* = 16.8 Hz, 2H, CH_2_COO−), 2.82–2.67 (m, 2H, CH_2_), 2.17–1.95 (m, 2H, C*H*_2_CH_3_), 0.82 (t, *J* = 7.4 Hz, 3H, CH_3_). ^13^C NMR (151 MHz, CDCl_3_): δ 173.25, 157.85 (d, *J*_C–F_ = 234.1 Hz), 138.32, 132.20, 126.91 (d, *J*_C–F_ = 9.4 Hz), 111.79 (d, *J*_C–F_ = 9.4 Hz), 109.96 (d, *J*_C–F_ = 26.7 Hz), 108.33 (d, *J*_C–F_ = 4.3 Hz), 103.29 (d, *J*_C–F_ = 23.1 Hz), 74.61, 60.57, 52.09, 42.66, 30.63, 22.31, 7.59. MS (ESI^+^) *m*/*z*: 292 [M + H]^+^.

#### General Procedure for the Preparation of Compounds **92**–**95**

The appropriate ester derivative **76**–**79** (1 equiv) was dissolved in DCM. The solution was stirred, cooled to 0 °C and *meta*-chloroperoxybenzoic acid (*m*CPBA, 3 equiv) was added. The mixture was stirred at RT overnight. The solution was then washed with NaOH 10% (3 × 15 mL). The combined organic phases were washed with brine (20 mL), dried over Na_2_SO_4,_ and evaporated to dryness. The crude product was purified by flash column chromatography to afford the corresponding product **92–95**. The products were characterized by MS (ESI) and used directly in the next step.

##### Methyl 2-(1-Methyl-8-(methylsulfonyl)-1,3,4,9-tetrahydropyrano­[3,4-*b*]­indol-1-yl)­acetate (**92**)

The reaction was run with **78** (0.19 g, 0.62 mmol), mCPBA (0.32 g, 1.87 mmol), DCM (9 mL). After purification by flash column chromatography (silica gel, DCM/MeOH 98:2) the product **92** was obtained as a white solid (46 mg, 22% yield). MS (ESI^+^) *m*/*z*: 338 [M + H]^+^.

##### Methyl 2-(1-Ethyl-8-(methylsulfonyl)-1,3,4,9-tetrahydropyrano­[3,4-*b*]­indol-1-yl)­acetate (**93**)

The reaction was run with **79** (0.64 g, 2 mmol), *m*CPBA (1.04 g, 6 mmol), DCM (27 mL). After purification by flash column chromatography (silica gel, DCM/MeOH 98:2) the product **93** was obtained as a white solid (232 mg, 33% yield). MS (ESI^+^) *m*/*z*: 352 [M + H]^+^.

##### Methyl 2-(1-Methyl-6-(methylsulfonyl)-1,3,4,9-tetrahydropyrano­[3,4-*b*]­indol-1-yl)­acetate (**94**)

The reaction was run with **76** (0.10 g, 0.33 mmol), mCPBA (0.17 g, 0.99 mmol), DCM (5 mL). After purification by flash column chromatography (silica gel, DCM/MeOH 97:3) the product **94** was obtained as a white solid (58 mg, 52% yield). MS (ESI^+^) *m*/*z*: 338 [M + H]^+^.

##### Methyl 2-(1-Ethyl-6-fluoro-8-(methylsulfonyl)-1,3,4,9-tetrahydropyrano­[3,4-*b*]­indol-1-yl)­acetate (**95**)

The reaction was run with **77** (0.215 g, 0.64 mmol), *m*CPBA (0.33 g, 1.9 mmol), DCM (9 mL). After purification by flash column chromatography (silica gel, DCM/MeOH 98:2) the product **95** was obtained as a white solid (73 mg, 31% yield). MS (ESI^+^) *m*/*z*: 370 [M + H]^+^.

#### General Procedure for the Preparation of Compounds **4**–**20**

To a solution of the appropriate ester derivative **77**, **80–95** (1 equiv) in 1,4-dioxane, an aqueous solution of LiOH 3 M (5 equiv) was added and the mixture was stirred at RT for 16 h. The reaction was quenched by adding a solution of HCl 2 M and the mixture was extracted with EtOAc (3 × 20 mL). The extracts were washed with brine (30 mL), dried over Na_2_SO_4_ and evaporated to dryness. The crude product was purified by flash column chromatography to afford the corresponding product **4–20**.

##### 2-(8-Ethyl-1-methyl-1,3,4,9-tetrahydropyrano­[3,4-*b*]­indol-1-yl)­acetic Acid (**4**)

The reaction was run with **85** (0.32 g, 1.11 mmol), LiOH 3 M (1.85 mL, 5.55 mmol), 1,4-dioxane (15 mL). After purification by flash column chromatography (silica gel, DCM/MeOH 9:1) the product **4** was obtained as a pale-yellow solid (291 mg, 96% yield). ^1^H NMR (300 MHz, CDCl_3_): δ 8.59 (s, 1H, NH), 7.28 (d, *J* = 7.7 Hz, 1H, ArH_5_), 7.04–6.90 (m, 2H, ArH_6–7_), 4.17–4.09 (m, 2H, OCH_2_−), 3.09 and 3.03 (two signals, d, *J* = 16.5 Hz, 2H, *CH*_2_COOH), 2.89–2.63 (m, 4H, CH_2_ and ArC*H*_2_CH_3_), 1.66 (s, 3H, CH_3_), 1.24 (t, *J* = 7.5 Hz, 3H, ArCH_2_C*H*_3_). ^13^C NMR (151 MHz, CD_3_OD): δ 174.8, 138.3, 136.1, 127.9, 127.8, 121.2, 120.2, 116.6, 107.5, 74.4, 61.7, 45.8, 25.9, 25.1, 23.3, 14.8. MS (ESI^–^) *m*/*z*: 272 [M – H]^−^.

##### 2-(1-Benzyl-8-ethyl-1,3,4,9-tetrahydropyrano­[3,4-*b*]­indol-1-yl)­acetic Acid (**5**)

The reaction was run with **86** (0.38 g, 1.05 mmol), LiOH 3 M (1.75 mL, 5.25 mmol), 1,4-dioxane (20 mL). After purification by flash column chromatography (silica gel, DCM/MeOH 98:2) the product **5** was obtained as a yellow solid (283 mg, 77% yield). ^1^H NMR (300 MHz, CDCl_3_): δ 8.70 (s, 1H, NH), 7.44–7.00 (m, 8H, ArH_5,6,7–2′,3′,4′,5′,6′_), 4.18 (d, *J* = 4.7 Hz, 2H, OCH_2_−), 3.48 and 3.35 (two signals, d, *J* = 14.3 Hz, 2H, *CH*_2_COOH), 3.49–3.29 (m, 2H, CH_2_Ph), 3.02–2.86 (m, 2H, C*H*_2_CH_3_), 2.86–2.61 (m, 2H, CH_2_), 1.32 (t, *J* = 7.6 Hz, 3H, CH_3_). ^13^C NMR (151 MHz, CDCl_3_): δ 174.2, 136.2, 135.0, 134.5, 130.7, 128.3, 126.9, 126.8, 126.2, 120.8, 119.8, 116.2, 108.5, 75.2, 60.8, 43.1, 42.7, 24.2, 22.4, 13.9. MS (ESI^–^) *m*/*z*: 348 [M – H]^−^.

##### 2-(8-Chloro-1-methyl-1,3,4,9-tetrahydropyrano­[3,4-*b*]­indol-1-yl)­acetic Acid (**6**)

The reaction was run with **87** (0.22 g, 0.75 mmol), LiOH 3 M (1.25 mL, 3.75 mmol), 1,4-dioxane (10 mL). After purification by flash column chromatography (silica gel, DCM/MeOH 9:1) the product **6** was obtained as a pale-yellow solid (172 mg, 82% yield). ^1^H NMR (300 MHz, CDCl_3_): δ 9.00 (s, 1H, NH), 7.41 (d, *J* = 7.7 Hz, 1H, ArH_7_), 7.18 (d, *J* = 7.7, 1H, ArH_5_), 7.05 (t, *J* = 7.7 Hz, 1H, ArH_6_), 4.11 (m, 2H, OCH_2_−), 3.08 (s, 2H, *CH*_2_COOH), 2.84 (m, 2H, CH_2_), 1.74 (s, 3H, CH_3_). ^13^C NMR (75 MHz, CDCl_3_): δ 175.47, 137.05, 133.48, 128.35, 121.97, 120.80, 117.43, 117.11, 108.77, 73.10, 61.17, 45.58, 25.89, 22.65. MS (ESI^–^) *m*/*z*: 278/280 [M – H]^−^.

##### 2-(8-Chloro-1-ethyl-1,3,4,9-tetrahydropyrano­[3,4-*b*]­indol-1-yl)­acetic Acid (**7**)

The reaction was run with **88** (0.08 g, 0.26 mmol), LiOH 3 M (0.43 mL, 1.3 mmol), 1,4-dioxane (10 mL). After purification by flash column chromatography (silica gel, DCM/MeOH 95:5) the product **7** was obtained as a white solid (60 mg, 78% yield). ^1^H NMR (600 MHz, CD_3_OD): δ 7.37–7.34 (m, 1H, ArH_7_), 7.09–7.07 (m, 1H, ArH_5_), 6.98–6.94 (m, 1H, ArH_6_), 4.05–3.98 (m, 2H, OCH_2_−), 3.03 and 2.86 (two signals, d, *J* = 14.5 Hz, 2H, *CH*_2_COOH), 2.80–2.67 (m, 2H, CH_2_), 2.13–2.09 (m, 2H, C*H*_2_CH_3_), 0.75 (t, *J* = 7.2 Hz, 3H, CH_3_). ^13^C NMR (151 MHz, CD_3_OD): δ 173.19, 137.09, 133.27, 128.37, 120.57, 119.41, 116.35, 116.16, 108.78, 75.61, 60.41, 42.43, 30.77, 21.85, 6.76. MS (ESI^–^) *m*/*z*: 292/294 [M – H]^−^.

##### 2-(1-Benzyl-8-chloro-1,3,4,9-tetrahydropyrano­[3,4-*b*]­indol-1-yl)­acetic Acid (**8**)

The reaction was run with **89** (0.26 g, 0.70 mmol), LiOH 3 M (1.17 mL, 3.5 mmol), 1,4-dioxane (10 mL). After purification by flash column chromatography (silica gel, DCM/MeOH 98:2) the product **8** was obtained as a white solid (244 mg, 98% yield). ^1^H NMR (600 MHz, CD_3_OD): δ 7.25 (dd, *J* = 7.8, 0.8 Hz, 1H, ArH_7_), 7.15–7.03 (m, 6H, ArH_2′‑3′‑4′‑5′‑6′‑5_), 6.90 (t, *J* = 7.7 Hz, 1H, ArH_6_), 3.97–3.89 (m, 2H, OCH_2_−), 3.32 and 3.25 (two signals, d, *J* = 14.1 Hz, 2H, CH_2_Ph), 2.91 and 2.70 (two signals, d, *J* = 14.8 Hz, 2H, *CH*_2_COOH), 2.64–2.40 (m, 2H, CH_2_). ^13^C NMR (151 MHz, CD_3_OD): δ 173.31, 136.70, 136.61, 133.15, 130.31, 128.28, 127.35, 126.08, 120.58, 119.38, 116.41, 116.17, 109.19, 75.56, 60.54, 43.39, 42.94, 21.68. MS (ESI^–^) *m*/*z*: 354/356 [M – H]^−^.

##### 2-(1-Methyl-8-(methylsulfonyl)-1,3,4,9-tetrahydropyrano­[3,4-*b*]­indol-1-yl)­acetic Acid (**9**)

The reaction was run with **92** (0.046 g, 0.14 mmol), LiOH 3 M (0.23 mL, 0.7 mmol), 1,4-dioxane (5 mL). After purification by flash column chromatography (silica gel, DCM/MeOH 95:5) the product **9** was obtained as a white solid (41 mg, 90% yield). ^1^H NMR (600 MHz, CD_3_OD): δ 7.77 (dd, *J* = 7.9, 1.0 Hz, 1H, ArH_7_), 7.60 (dd, *J* = 7.6, 1.0 Hz, 1H, ArH_5_), 7.20 (t, *J* = 7.7 Hz, 1H, ArH_6_), 4.07–4.02 (m, 2H, OCH_2_−), 3.14 (s, 3H, SO_2_C*H*_3_), 2.99 and 2.93 (two signals, d, *J* = 15.8 Hz, 2H, *CH*_2_COOH), 2.83–2.75 (m, 2H, CH_2_), 1.69 (s, 3H, CH_3_). ^13^C NMR (151 MHz, CD_3_OD): δ 173.52, 139.71, 131.49, 129.11, 124.15, 122.05, 121.53, 118.58, 107.50, 72.60, 59.96, 44.60, 43.11, 24.33, 21.64. MS (ESI^–^) *m*/*z*: 322 [M – H]^−^.

##### 2-(1-Ethyl-8-(methylsulfonyl)-1,3,4,9-tetrahydropyrano­[3,4-*b*]­indol-1-yl)­acetic Acid (**10**)

The reaction was run with **93** (0.232 g, 0.66 mmol), LiOH 3 M (1.1 mL, 3.3 mmol), 1,4-dioxane (20 mL). After purification by flash column chromatography (silica gel, DCM/MeOH 95:5) the product **10** was obtained as a yellow solid (194 mg, 87% yield). ^1^H NMR (600 MHz, CDCl_3_): δ 9.97 (s, 1H, NH), 7.75 (d, *J* = 7.7 Hz, 1H, ArH_7_), 7.63 (d, *J* = 7.6 Hz, 1H, ArH_5_), 7.22 (t, *J* = 7.7 Hz, 1H, ArH_6_), 4.12–3.99 (m, 2H, OCH_2_−), 3.08 (s, 3H, SO_2_C*H*_3_), 3.06 and 2.96 (two signals, d, *J* = 16.5 Hz, 2H, *CH*_2_COOH), 2.89–2.79 (m, 2H, CH_2_), 2.19–2.04 (m, 2H, C*H*_2_CH_3_), 0.86 (t, *J* = 7.4 Hz, 3H, CH_3_). ^13^C NMR (151 MHz, CDCl_3_): δ 174.74, 138.08, 132.02, 128.90, 124.62, 122.15, 121.97, 119.37, 109.08, 74.97, 60.53, 44.85, 42.39, 30.76, 22.08, 7.69. MS (ESI^–^) *m*/*z*: 336 [M – H]^−^.

##### 2-(1-Methyl-6-(methylsulfonyl)-1,3,4,9-tetrahydropyrano­[3,4-*b*]­indol-1-yl)­acetic Acid (**11**)

The reaction was run with **94** (0.04 g, 0.12 mmol), LiOH 3 M (0.2 mL, 0.6 mmol), 1,4-dioxane (4 mL). After purification by flash column chromatography (silica gel, DCM/MeOH 98:2) the product **11** was obtained as a white solid (17 mg, 43% yield). ^1^H NMR (300 MHz, CDCl_3_): δ 9.38 (s, 1H, NH), 8.06 (s, 1H, ArH_5_), 7.60 (d, *J* = 8.5 Hz, 1H, ArH_7_), 7.33 (d, *J* = 8.5 Hz, 1H, ArH_8_), 4.10–4.00 (m, 2H, OCH_2_−), 3.01 (s, 3H, SO_2_C*H*_3_), 2.95 (d, *J* = 6.6 Hz, 2H, *CH*_2_COOH), 2.80–2.71 (m, 2H, CH_2_), 1.63 (s, 3H, CH_3_). ^13^C NMR (75 MHz, CDCl_3_): δ 174.44, 139.41, 138.56, 131.56, 126.64, 120.84, 119.69, 112.38, 109.17, 72.84, 60.97, 45.68, 45.31, 25.71, 22.34. MS (ESI^–^) *m*/*z*: 322 [M – H]^−^.

##### 2-(6-Fluoro-1-methyl-1,3,4,9-tetrahydropyrano­[3,4-*b*]­indol-1-yl)­acetic Acid (**12**)

The reaction was run with **90** (0.96 g, 3.46 mmol), LiOH 3 M (5.77 mL, 17.3 mmol), 1,4-dioxane (40 mL). After purification by flash column chromatography (silica gel, DCM/MeOH 9:1) the product **12** was obtained as a white-pink solid (683 mg, 75% yield). ^1^H NMR (300 MHz, CDCl_3_): δ 8.64 (s, 1H, NH), 7.15 (m, 1H, ArH_8_), 7.05 (dd, *J* = 9.4, 2.4 Hz, 1H, ArH_7_), 6.83 (td, *J* = 9.1, 2.5 Hz, 1H, ArH_5_), 4.09–3.95 (m, 2H, OCH_2_−), 3.15 and 3.07 (two signals, d, *J* = 16.0 Hz, 2H, *CH*_2_COOH), 2.82–2.64 (m, 2H, CH_2_), 1.62 (s, 3H, CH_3_). ^13^C NMR (75 MHz, CDCl_3_): δ 174.99, 158.28 (d, *J*_C–F_ = 233.25 Hz), 137.97, 132.64, 127.19 (d, *J*_C–F_ = 9.75 Hz), 112.22 (d, *J*_C–F_ = 9 Hz), 110.80 (d, *J*_C–F_ = 26.25 Hz), 107.82 (d, *J*_C–F_ = 4.50 Hz), 103.85 (d, *J*_C–F_ = 23.25 Hz), 73.08, 61.24, 45.56, 25.85, 22.47. MS (ESI^–^) *m*/*z*: 262 [M – H]^−^.

##### 2-(1-Ethyl-6-fluoro-1,3,4,9-tetrahydropyrano­[3,4-*b*]­indol-1-yl)­acetic Acid (**13**)

The reaction was run with **91** (0.20 g, 0.69 mmol), LiOH 3 M (1.14 mL, 3.43 mmol), 1,4-dioxane (20 mL). After purification by flash column chromatography (silica gel, PE/EtOAc/MeOH 7:2:1) the product **13** was obtained as a white solid (186 mg, 98% yield). ^1^H NMR (300 MHz, CDCl_3_): δ 8.60 (s, 1H, NH), 7.12 (m, 1H, ArH_8_), 7.01 (dd, *J* = 9.4, 2.4 Hz, 1H, ArH_7_), 6.80 (dd, *J* = 9.4, 2.5 Hz, 1H, ArH_5_), 3.98 (m, 2H, OCH_2_−), 2.90 (m, 2H, *CH*_2_COOH), 2.70 (m, 2H, CH_2_), 2.25–2.18 (m, 2H, C*H*_2_CH_3_), 1.58 (s, 3H, CH_3_). ^13^C NMR (75 MHz, CDCl_3_): δ 174.77, 155.89 (d, *J* = 234.25 Hz), 142.31, 129.99, 129.64, 127.50 (d, *J*_C–F_ = 7.5 Hz), 108.79 (d, *J* = 20.2 Hz), 106.41 (d, *J* = 5.9 Hz), 102.14 (d, *J* = 22.9 Hz), 86.41, 74.89, 59.35, 53.53, 22.13, 7.86. MS (ESI^–^) *m*/*z*: 276 [M – H]^−^.

##### 2-(8-Chloro-6-fluoro-1-methyl-1,3,4,9-tetrahydropyrano­[3,4-*b*]­indol-1-yl)­acetic Acid (**14**)

The reaction was run with **82** (0.30 g, 0.96 mmol), LiOH 3 M (1.6 mL, 4.81 mmol), 1,4-dioxane (30 mL). After purification by flash column chromatography (silica gel, DCM/MeOH 9:1) the product **14** was obtained as a white solid (234 mg, 82% yield). ^1^H NMR (300 MHz, CDCl_3_): δ 8.97 (s, 1H, NH), 7.07 (dd, *J* = 9.0, 2.1 Hz, 1H, ArH_7_), 6.97 (dd, *J* = 9.0, 2.2 Hz, 1H, ArH_5_), 4.10–3.94 (m, 2H, OCH_2_−), 3.07 (s, 2H, *CH*_2_COOH), 2.91–2.66 (m, 2H, CH_2_), 1.73 (s, 3H, CH_3_). ^13^C NMR (75 MHz, CDCl_3_): δ 175.37, 157.62 (d, *J*_C–F_ = 236.25 Hz), 138.81, 130.24, 127.75 (d, *J*_C–F_ = 10.5 Hz), 116.91 (d, *J*_C–F_ = 12.7 Hz), 110.73 (d, *J*_C–F_ = 29.25 Hz), 109.04 (d, *J*_C–F_ = 4.5 Hz), 102.86 (d, *J*_C–F_ = 23, 25 Hz), 72.98, 61.07, 45.47, 25.83, 22.55. MS (ESI^–^) *m*/*z*: 296/298 [M – H]^−^.

##### 2-(8-Chloro-1-ethyl-6-fluoro-1,3,4,9-tetrahydropyrano­[3,4-*b*]­indol-1-yl)­acetic Acid (**15**)

The reaction was run with **81** (0.20 g, 0.61 mmol), LiOH 3 M (1.02 mL, 3.07 mmol), 1,4-dioxane (20 mL). After purification by flash column chromatography (silica gel, DCM/MeOH 9:1) the product **15** was obtained as a white solid (190 mg, 100% yield). ^1^H NMR (300 MHz, CDCl_3_): δ 8.93 (s, 1H, NH), 7.08 (dd, *J* = 8.9, 1.6 Hz, 1H, ArH_7_), 6.99 (dd, *J* = 9.3, 2.2 Hz, 1H, ArH_5_), 4.10–3.93 (m, 2H, OCH_2_−), 3.09 and 3.03 (two signals, d, *J* = 16.5 Hz, 2H, *CH*_2_COOH), 2.87–2.67 (m, 2H, CH_2_), 2.23–2.00 (m, 2H, C*H*_2_CH_3_), 0.88 (t, *J* = 7.3 Hz, 3H, CH_3_). ^13^C NMR (75 MHz, CDCl_3_): δ 176.13, 157.5 (d, *J*_C–F_ = 236.25 Hz), 138.25, 130.25, 127.71 (d, *J*_C–F_ = 10.4 Hz), 116.78 (d, *J*_C–F_ = 2.25 Hz), 110.64 (d, *J*_C–F_ = 28.5 Hz), 110.00 (d, *J*_C–F_ = 5.25 Hz), 102.79 (d, *J*_C–F_ = 23.25 Hz), 75.32, 61.00, 42.78, 31.14, 22.53, 8.00. MS (ESI^–^) *m*/*z*: 310/312 [M – H]^−^.

##### 2-(1-Benzyl-8-chloro-6-fluoro-1,3,4,9-tetrahydropyrano­[3,4-*b*]­indol-1-yl)­acetic Acid (**16**)

The reaction was run with **83** (0.19 g, 0.49 mmol), LiOH 3 M (0.82 mL, 2.45 mmol), 1,4-dioxane (8 mL). After purification by flash column chromatography (silica gel, DCM/MeOH 98:2) the product **16** was obtained as a white solid (180 mg, 98% yield). ^1^H NMR (600 MHz, CD_3_OD): δ 10.68 (s, 1H, NH), 7.13–7.08 (m, 3H, ArH_2′‑4′‑6′_), 7.08–7.04 (m, 2H, ArH_3′–5′_), 6.98 (dd, *J* = 9.2, 2.3 Hz, 1H, ArH_7_), 6.90 (dd, *J* = 9.2, 2.3 Hz, 1H, ArH_5_), 3.95 (t, *J* = 5.4 Hz, 2H, OCH_2_−), 3.37 and 3.28 (two signals, d, *J* = 14.1 Hz, 2H, *CH*_2_COOH), 2.99–2.69 (m, 2H, CH_2_Ph), 2.62–2.36 (m, 2H, CH_2_). ^13^C NMR (151 MHz, CD_3_OD): δ 173.05, 156.90 (d, *J*_C–F_ = 235.5 Hz), 138.89 (d, *J*_C–F_ = 20.7 Hz), 136.51, 130.29, 130.02, 127.72, 127.35, 126.11, 116.00, 109.65, 108.91 (d, *J*_C–F_ = 29.5 Hz), 101.50 (d, *J*_C–F_ = 23.3 Hz), 75.58, 60.54, 43.49, 42.88, 21.56. MS (ESI^–^) *m*/*z*: 372/374 [M – H]^−^.

##### 2-(8-Chloro-1-ethyl-6-methyl-1,3,4,9-tetrahydropyrano­[3,4-*b*]­indol-1-yl)­acetic Acid (**17**)

The reaction was run with **84** (0.33 g, 1.02 mmol), LiOH 3 M (1.71 mL, 5.13 mmol), 1,4-dioxane (15 mL). After purification by flash column chromatography (silica gel, DCM/MeOH 95:5) the product **17** was obtained as a yellow solid (286 mg, 91% yield). ^1^H NMR (600 MHz, CD_3_OD): δ 10.24 (s, 1H, NH), 7.11 (s, 1H, ArH_7_), 6.90 (s, 1H, ArH_5_), 4.04–3.92 (m, 2H, OCH_2_−), 2.98 and 2.84 (two signals, d, *J* = 14.5 Hz, 2H, *CH*_2_COOH), 2.77–2.58 (m, 2H, CH_2_), 2.35 (s, 3H, ArCH_3_), 2.09 (q, *J* = 7.6 Hz, 2H, C*H*_2_CH_3_), 0.71 (t, *J* = 7.4 Hz, 3H, CH_3_). ^13^C NMR (151 MHz, CD_3_OD): δ 173.18, 137.17, 131.60, 129.10, 128.48, 121.84, 116.17, 115.66, 108.26, 75.63, 60.41, 42.41, 30.75, 21.86, 19.97, 6.76. MS (ESI^–^) *m*/*z*: 306/308 [M – H]^−^.

##### 2-(1-Ethyl-6-fluoro-8-(methylsulfonyl)-1,3,4,9-tetrahydropyrano­[3,4-*b*]­indol-1-yl)­acetic Acid (**18**)

The reaction was run with **95** (0.075 g, 0.20 mmol), LiOH 3 M (0.33 mL, 1 mmol), 1,4-dioxane (7 mL). After purification by flash column chromatography (silica gel, DCM/MeOH 95:5) the product **18** was obtained as a white solid (64 mg, 90% yield). ^1^H NMR (600 MHz, CD_3_OD): δ 7.51 (dd, *J* = 9.0, 2.4 Hz, 1H, ArH_7_), 7.38 (dd, *J* = 8.8, 2.4 Hz, 1H, ArH_5_), 4.06–3.92 (m, 2H, OCH_2_−), 3.17 (s, 3H, SO_2_CH_3_), 2.98 and 2.92 (two signals, d, *J* = 15.7 Hz, 2H, *CH*_2_COOH), 2.78–2.68 (m, 2H, CH_2_), 2.20–1.98 (m, 2H, C*H*_2_CH_3_), 0.79 (t, *J* = 7.4 Hz, 3H, CH_3_). ^13^C NMR (75 MHz, CD_3_OD): δ 173.01, 156.09 (d, *J*_C–F_ = 235.5 Hz), 142.22, 130.18 (d, *J*_C–F_ = 9.1 Hz), 128.53, 123.73 (d, *J*_C–F_ = 8.1 Hz), 110.39 (d, *J*_C–F_ = 23.1 Hz), 109.45 (d, *J*_C–F_ = 4.6 Hz), 109.37 (d, *J*_C–F_ = 28.5 Hz), 75.77, 60.30, 44.03, 42.84, 30.89, 22.44, 8.45. MS (ESI^–^) *m*/*z*: 354 [M – H]^−^.

##### 2-(1-Ethyl-6-fluoro-8-(methylthio)-1,3,4,9-tetrahydropyrano­[3,4-*b*]­indol-1-yl)­acetic Acid (**19**)

The reaction was run with **77** (0.154 g, 0.46 mmol), LiOH 3 M (0.76 mL, 2.28 mmol), 1,4-dioxane (14 mL). After purification by flash column chromatography (silica gel, DCM/MeOH 95:5) the product **19** was obtained as a colorless oil (114 mg, 77% yield). ^1^H NMR (600 MHz, CD_3_OD): δ 8.89 (s, 1H, NH), 7.01 (dd, *J* = 9.1, 2.2 Hz, 1H, ArH_7_), 6.91 (dd, *J* = 9.6, 2.4 Hz, 1H, ArH_5_), 4.10–4.00 (m, 2H, OCH_2_−), 3.07 and 3.02 (two signals, d, *J* = 16.5 Hz, 2H, *CH*_2_COOH), 2.81–2.71 (m, 2H, CH_2_), 2.47 (s, 3H, SCH_3_), 2.14 (dd, *J* = 14.6, 7.4 Hz, 2H, C*H*_2_CH_3_), 0.86 (t, *J* = 7.4 Hz, 3H, CH_3_). ^13^C NMR (151 MHz, CD_3_OD): δ 175.70, 158.00 (d, *J*_C–F_ = 236.5 Hz), 137.32, 131.95, 126.20 (d, *J*_C–F_ = 10.1 Hz), 120.60 (d, *J*_C–F_ = 9.8 Hz), 110.58 (d, *J*_C–F_ = 27.5 Hz), 109.22 (d, *J*_C–F_ = 5.0 Hz), 102.05 (d, *J*_C–F_ = 23.8 Hz), 75.09, 60.74, 42.62, 30.91, 22.23, 17.08, 7.72. MS (ESI^–^) *m*/*z*: 322 [M – H]^−^.

##### 2-(1-Ethyl-6-fluoro-8-iodo-1,3,4,9-tetrahydropyrano­[3,4-*b*]­indol-1-yl)­acetic Acid (**20**)

The reaction was run with **80** (0.35 g, 0.84 mmol), LiOH 3 M (1.40 mL, 4.19 mmol), 1,4-dioxane (10 mL). After purification by flash column chromatography (silica gel, DCM/MeOH 95:5) the product **20** was obtained as a white solid (325 mg, 96% yield). ^1^H NMR (600 MHz, CD_3_OD): δ 7.23 (dd, *J* = 8.7, 2.3 Hz, 1H, ArH_7_), 7.11 (dd, *J* = 9.2, 2.3 Hz, 1H, ArH_5_), 4.03–3.92 (m, 2H, OCH_2_−), 2.99 and 2.91 (two signals, d, *J* = 14.8 Hz, 2H, *CH*_2_COOH), 2.75–2.65 (m, 2H, CH_2_), 2.16–2.04 (m, 2H, C*H*_2_CH_3_), 0.74 (t, *J* = 7.4 Hz, 3H, CH_3_). ^13^C NMR (151 MHz, CD_3_OD): δ 175.07, 158.62 (d, *J*_C–F_ = 237.7 Hz), 140.91, 136.94, 128.04 (d, *J*_C–F_ = 9.9 Hz), 119.56 (d, *J*_C–F_ = 28.6 Hz), 111.14 (d, *J*_C–F_ = 4.3 Hz), 104.39 (d, *J*_C–F_ = 23.2 Hz), 77.03, 75.62 (d, *J*_C–F_ = 11.1 Hz), 61.84, 43.79, 32.13, 23.52, 8.32. MS (ESI^–^) *m*/*z*: 402 [M – H]^−^.

#### General Procedure for the Preparation of Compounds **97a**–**f**, **97h**–**n**

To a stirred solution of **80** (1 equiv) in a 1:1 mixture of DME/H_2_O, Pd­(dba)_2_ (0.1 equiv), the appropriate boronic acid **96a–f**, **96h–n** (1 equiv) and K_2_CO_3_ (2 equiv) were added, and the mixture was heated at 90 °C and stirred under a N_2_ atmosphere until complete consumption of the starting material, as indicated by TLC. The mixture was cooled to RT and filtered, the filtrate was extracted with EtOAc (3 × 20 mL) (or acidified by treatment with HCl 2 M before the extraction with EtOAc if specified), the organic extracts were washed with brine, dried over Na_2_SO_4,_ and evaporated to dryness. The crude product was purified by flash column chromatography to afford the corresponding product **97a**–**f** and **97h**–**n**. The products were characterized by MS (ESI) and used directly in the next step.

##### Methyl 2-(1-Ethyl-6-fluoro-8-phenyl-1,3,4,9-tetrahydropyrano­[3,4-*b*]­indol-1-yl)­acetate (**97a**)

The reaction was run with **80** (0.080 g, 0.19 mmol), phenylboronic acid (**96a**, 0.024 mg, 0.19 mmol), K_2_CO_3_ (0.053 g, 0.38 mmol), Pd­(dba)_2_ (0.011 g, 0.019 mmol), DME/H_2_O (4 mL). Reaction time: 2 h. After purification by flash column chromatography (silica gel, PE/DCM 6:4) the product **97a** was obtained as a white solid (66 mg, 95% yield). MS (ESI^–^) *m*/*z*: 366 [M – H]^−^.

##### Methyl 2-(8-(2-Chlorophenyl)-1-ethyl-6-fluoro-1,3,4,9-tetrahydropyrano­[3,4-*b*]­indol-1-yl)­acetate (**97b**)

The reaction was run with **80** (0.150 g, 0.36 mmol), (2-chlorophenyl)­boronic acid (**96b**, 0.056 g, 0.36 mmol), K_2_CO_3_ (0.100 g, 0.72 mmol), Pd­(dba)_2_ (0.021 g, 0.036 mmol), DME/H_2_O (7 mL). Reaction time: 30 min. After purification by flash column chromatography (silica gel, PE/EtOAc 9:1) the product **97b** was obtained as a white solid (59 mg, 41% yield). MS (ESI^–^) *m*/*z*: 400/402 [M – H]^−^.

##### Methyl 2-(8-(3-Chlorophenyl)-1-ethyl-6-fluoro-1,3,4,9-tetrahydropyrano­[3,4-*b*]­indol-1-yl)­acetate (**97c**)

The reaction was run with **80** (0.200 g, 0.48 mmol), (3-chlorophenyl)­boronic acid (**96c**, 0.075 mg, 0.48 mmol), K_2_CO_3_ (0.133 g, 0.96 mmol), Pd­(dba)_2_ (0.028 g, 0.048 mmol), DME/H_2_O (8 mL). Reaction time: 10 min. After purification by flash column chromatography (silica gel, PE/DCM 1:1) the product **97c** was obtained as a yellow oil (110 mg, 57% yield). MS (ESI^–^) *m*/*z*: 400/402 [M – H]^−^.

##### Methyl 2-(8-(4-Chlorophenyl)-1-ethyl-6-fluoro-1,3,4,9-tetrahydropyrano­[3,4-*b*]­indol-1-yl)­acetate (**97d**)

The reaction was run with **80** (0.150 g, 0.36 mmol), (4-chlorophenyl)­boronic acid (**96d**, 0.056 g, 0.36 mmol), K_2_CO_3_ (0.100 g, 0.72 mmol), Pd­(dba)_2_ (0.021 g, 0.036 mmol), DME/H_2_O (7 mL). Reaction time: 30 min. After purification by flash column chromatography (silica gel, pure DCM) the product **97d** was obtained as a white solid (117 mg, 81% yield). MS (ESI^–^) *m*/*z*: 400/402 [M – H]^−^.

##### Methyl 2-(1-Ethyl-6-fluoro-8-(2-hydroxyphenyl)-1,3,4,9-tetrahydropyrano­[3,4-*b*]­indol-1-yl)­acetate (**97e**)

The reaction was run with **80** (0.150 g, 0.36 mmol), (2-hydroxyphenyl)­boronic acid (**96e**, 0.050 g, 0.36 mmol), K_2_CO_3_ (0.100 g, 0.72 mmol), Pd­(dba)_2_ (0.021 g, 0.036 mmol), DME/H_2_O (7 mL). Reaction time: 20 min. The reaction mixture was treated with HCl 2 M after filtration and before the extraction with EtOAc. After purification by flash column chromatography (silica gel, PE/EtOAc/MeOH 7:2:1) the product **97e** was obtained as an off-white solid (130 mg, 94% yield). MS (ESI^–^) *m*/*z*: 382 [M – H]^−^.

##### Methyl 2-(1-Ethyl-6-fluoro-8-(3-hydroxyphenyl)-1,3,4,9-tetrahydropyrano­[3,4-*b*]­indol-1-yl)­acetate (**97f**)

The reaction was run with **80** (0.150 g, 0.36 mmol), (3-hydroxyphenyl)­boronic acid (**96f**, 0.050 g, 0.36 mmol), K_2_CO_3_ (0.100 g, 0.72 mmol), Pd­(dba)_2_ (0.021 g, 0.036 mmol), DME/H_2_O (7 mL). Reaction time: 20 min. The reaction mixture was treated with HCl 2 M after filtration and before the extraction with EtOAc. After purification by flash column chromatography (silica gel, PE/EtOAc/MeOH 7:2:1) the product **97f** was obtained as a yellow oil (137 mg, 99% yield). MS (ESI^–^) *m*/*z*: 382 [M – H]^−^.

##### Methyl 2-(1-Ethyl-6-fluoro-8-(2-(hydroxymethyl)­phenyl)-1,3,4,9-tetrahydropyrano­[3,4-*b*]­indol-1-yl)­acetate (**97h**)

The reaction was run with **80** (0.150 g, 0.36 mmol), (2-(hydroxymethyl)­phenyl)­boronic acid (**96h**, 0.055 g, 0.36 mmol), K_2_CO_3_ (0.100 g, 0.72 mmol), Pd­(dba)_2_ (0.021 g, 0.036 mmol), DME/H_2_O (7 mL). Reaction time: 20 min. After purification by flash column chromatography (silica gel, PE/EtOAc/MeOH 8:1.5:0.5) the product **97h** was obtained as a white solid (124 mg, 87% yield). MS (ESI^–^) *m*/*z*: 396 [M – H]^−^.

##### Methyl 2-(1-Ethyl-6-fluoro-8-(3-(hydroxymethyl)­phenyl)-1,3,4,9-tetrahydropyrano­[3,4-*b*]­indol-1-yl)­acetate (**97i**)

The reaction was run with **80** (0.200 g, 0.48 mmol), (3-(hydroxymethyl)­phenyl)­boronic acid (**96i**, 0.073 g, 0.48 mmol), K_2_CO_3_ (0.133 g, 0.96 mmol), Pd­(dba)_2_ (0.028 g, 0.048 mmol), DME/H_2_O (8 mL). Reaction time: 20 min. After purification by flash column chromatography (silica gel, PE/EtOAc/MeOH 8:1.5:0.5) the product **97i** was obtained as an off-white solid (189 mg, 99% yield). MS (ESI^–^) *m*/*z*: 396 [M – H]^−^.

##### Methyl 2-(1-Ethyl-6-fluoro-8-(4-(hydroxymethyl)­phenyl)-1,3,4,9-tetrahydropyrano­[3,4-*b*]­indol-1-yl)­acetate (**97j**)

The reaction was run with **80** (0.180 g, 0.43 mmol), (4-(hydroxymethyl)­phenyl)­boronic acid (**96j**, 0.066 g, 0.43 mmol), K_2_CO_3_ (0.119 g, 0.86 mmol), Pd­(dba)_2_ (0.025 g, 0.043 mmol), DME/H_2_O (7 mL). Reaction time: 15 min. After purification by flash column chromatography (silica gel, PE/EtOAc/MeOH 8:1.5:0.5) the product **97j** was obtained as a brown solid (123 mg, 72% yield). MS (ESI^–^) *m*/*z*: 396 [M – H]^−^.

##### Methyl 2-(8-(3-Acetamidophenyl)-1-ethyl-6-fluoro-1,3,4,9-tetrahydropyrano­[3,4-*b*]­indol-1-yl)­acetate (**97k**)

The reaction was run with **80** (0.200 g, 0.48 mmol), (3-acetamidophenyl)­boronic acid (**96k**, 0.086 mg, 0.48 mmol), K_2_CO_3_ (0.133 g, 0.96 mmol), Pd­(dba)_2_ (0.028 g, 0.048 mmol), DME/H_2_O (8 mL). Reaction time: 15 min. After purification by flash column chromatography (silica gel, PE/EtOAc/MeOH 7:2:1) the product **97k** was obtained as an orange solid (192 mg, 94% yield). MS (ESI^–^) *m*/*z*: 423 [M – H]^−^.

##### Methyl-2-[8-(3-carbamoylphenyl)-1-ethyl-6-fluoro-4,9-dihydro-3*H*-pyrano­[3,4-*b*]­indol-1-yl]­acetate (**97l**)

The reaction was run with **80** (0.150 g, 0.36 mmol), (3-carbamoylphenyl)­boronic acid (**96l**, 0.059 g, 0.36 mmol), K_2_CO_3_ (0.100 g, 0.72 mmol), Pd­(dba)_2_ (0.021 g, 0.036 mmol), DME/H_2_O (8 mL). Reaction time: 15 min. After purification by flash column chromatography (silica gel, DCM/MeOH 98:2) the product **97l** was obtained as a yellow solid (120 mg, 81% yield). MS (ESI^–^) *m*/*z*: 409 [M – H]^−^.

##### Methyl 2-(8-(3-(((*tert*-Butoxycarbonyl)­amino)­methyl)­phenyl)-1-ethyl-6-fluoro-1,3,4,9-tetrahydropyrano­[3,4-*b*]­indol-1-yl)­acetate (**97m**)

The reaction was run with **80** (0.200 g, 0.48 mmol), (3-{[(*tert*-butoxycarbonyl)­amino]­methyl}­phenyl)­boronic acid (**96m**, 0.121 g, 0.48 mmol), K_2_CO_3_ (0.133 g, 0.96 mmol), Pd­(dba)_2_ (0.028 g, 0.048 mmol), DME/H_2_O (9 mL). Reaction time: 35 min. After purification by flash column chromatography (silica gel, PE/EtOAc 8:2) the product **97m** was obtained as a yellow solid (207 mg, 87% yield). MS (ESI^–^) *m*/*z*: 495 [M – H]^−^.

##### Methyl 2-(8-(3-Acetylphenyl)-1-ethyl-6-fluoro-1,3,4,9-tetrahydropyrano­[3,4-*b*]­indol-1-yl)­acetate (**97n**)

The reaction was run with **80** (0.100 g, 0.24 mmol), (3-acetylphenyl)­boronic acid (**96n**, 0.039 g, 0.24 mmol), K_2_CO_3_ (0.066 g, 0.48 mmol), Pd­(dba)_2_ (0.014 g, 0.024 mmol), DME/H_2_O (4.7 mL). Reaction time: 30 min. After purification by flash column chromatography (silica gel, PE/EtOAc/MeOH 8.25:1.5:0.25) the product **97n** was obtained as a pale yellow solid (47 mg, 48% yield). MS (ESI^–^) *m*/*z*: 408 [M – H]^−^.

##### Methyl 2-(1-Ethyl-6-fluoro-8-(3-(methylsulfonyl)­phenyl)-1,3,4,9-tetrahydropyrano­[3,4-*b*]­indol-1-yl)­acetate (**97o**)

To a stirred solution of **80** (0.100 g, 0.24 mmol, 1 equiv) in 1,4-dioxane (5 mL), Pd­(PPh_3_)_4_ (0.028 g, 0.024 mmol, 0.1 equiv), an aqueous solution of K_2_CO_3_ (0.066 g, 0.48 mmol, 2 equiv, in 0.5 mL of H_2_O) and (3-(methylsulfonyl)­phenyl)­boronic acid (**96o**, 0.048 g, 0.24 mmol, 1 equiv) were added and the mixture was heated at 90 °C and stirred under a nitrogen atmosphere for 3 h. The mixture was then cooled to RT and filtered, the filtrate was extracted with DCM (3 × 20 mL), the organic extracts were washed with brine, dried over Na_2_SO_4_ and evaporated to dryness. The crude product was purified by flash column chromatography (silica gel, PE/EtOAc 6.5:3.5) to give **97o** as an off-white solid (102 mg, 95% yield). MS (ESI^–^) *m*/*z*: 444 [M – H]^−^.

#### General Procedure for the Preparation of Compounds **97g** and **97p**–**r**

To a solution of **80** (1 equiv) in 1,4-dioxane, Pd­(PPh_3_)_2_(OAc)_2_ (0.1 equiv) was added, and the mixture was vigorously stirred for 10 min under a nitrogen atmosphere. Then a solution of K_3_PO_4_ (2 equiv) in water (1.5 mL) was added and the reaction mixture was heated at 90 °C. The appropriate boronic acid **96g**, **96p**–**r** (1 equiv), dissolved in a mixture of 1,4-dioxane/H_2_O 9:1 (3.5 mL), was added dropwise and the reaction was stirred for 3–5 h at 90 °C. The reaction was monitored by TLC and 0.7 equiv of the appropriate boronic acid were added to the mixture. After 1 h, the reaction was cooled to RT and filtered. The filtrate was extracted with EtOAc (3 × 20 mL), the organic extracts were washed with brine (30 mL), dried over Na_2_SO_4_ and evaporated to dryness. The crude product was purified by flash column chromatography to afford the corresponding product **97g** and **97p**–**r**. The products were characterized by MS (ESI) and used directly in the next step.

##### Methyl 2-(1-Ethyl-6-fluoro-8-(4-hydroxyphenyl)-1,3,4,9-tetrahydropyrano­[3,4-*b*]­indol-1-yl)­acetate (**97g**)

The reaction was run with **80** (0.150 g, 0.36 mmol), (4-hydroxyphenyl)­boronic acid (**96g**, 0.050 g, 0.36 mmol), K_3_PO_4_ (0.153 g, 0.72 mmol), Pd­(PPh_3_)_2_(OAc)_2_ (0.027 g, 0.036 mmol), 1,4-dioxane/H_2_O (25 mL). After purification by flash column chromatography (silica gel, DCM/EtOAc 9.5:0.5) the product **97g** was obtained as a light-yellow oil (123 mg, 89% yield). MS (ESI^–^) *m*/*z*: 382 [M – H]^−^.

##### Methyl 2-(1-Ethyl-6-fluoro-8-(3-methoxyphenyl)-1,3,4,9-tetrahydropyrano­[3,4-*b*]­indol-1-yl)­acetate (**97p**)

The reaction was run with **80** (0.150 g, 0.36 mmol), (3-methoxyphenyl)­boronic acid (**96p**, 0.055 g, 0.36 mmol), K_3_PO_4_ (0.153 g, 0.72 mmol), Pd­(PPh_3_)_2_(OAc)_2_ (0.027 g, 0.036 mmol), 1,4-dioxane/H_2_O (22 mL). After purification by flash column chromatography (silica gel, PE/EtOAc 9:1) the product **97p** was obtained as a white solid (108 mg, 76% yield). MS (ESI^–^) *m*/*z*: 396 [M – H]^−^.

##### Methyl 2-(8-(2,5-Dichlorophenyl)-1-ethyl-6-fluoro-1,3,4,9-tetrahydropyrano­[3,4-*b*]­indol-1-yl)­acetate (**97q**)

The reaction was run with **80** (0.150 g, 0.36 mmol), (2,5-dichlorophenyl)­boronic acid (**96q**, 0.069 g, 0.36 mmol), K_3_PO_4_ (0.153 g, 0.72 mmol), Pd­(PPh_3_)_2_(OAc)_2_ (0.027 g, 0.036 mmol), 1,4-dioxane/H_2_O (25 mL). After purification by flash column chromatography (silica gel, PE/EtOAc 9.5:0.5) the product **97q** was obtained as a yellow oil (117 mg, 75% yield). MS (ESI^–^) *m*/*z*: 434/435/436 [M – H]^−^.

##### Methyl 2-(8-(3,5-Dichlorophenyl)-1-ethyl-6-fluoro-1,3,4,9-tetrahydropyrano­[3,4-*b*]­indol-1-yl)­acetate (**97r**)

The reaction was run with **80** (0.150 g, 0.36 mmol), (3,5-dichlorophenyl)­boronic acid (**96r**, 0.069 g, 0.36 mmol), K_3_PO_4_ (0.153 g, 0.72 mmol), Pd­(PPh_3_)_2_(OAc)_2_ (0.027 g, 0.036 mmol), 1,4-dioxane/H_2_O (25 mL). After purification by flash column chromatography (silica gel, PE/EtOAc 9.5:0.5) the product **97r** was obtained as a yellow oil (123 mg, 78% yield). MS (ESI^–^): 434/435/436 [M – H]^−^.

#### General Procedure for the Preparation of Compounds **21**, **23**, **25**, **27–35** and **37–41**

To a solution of the appropriate ester derivative **97a**–**l**, **97n**–**r** (1 equiv) in 1,4-dioxane, an aqueous solution of LiOH 3 M (5 equiv) was added and the mixture was stirred at RT for 16 h. The reaction was quenched by adding a solution of HCl 2 M (or phosphate buffer pH 7.4 where specified) and the mixture was extracted with EtOAc (3 × 20 mL). The extracts were washed with brine (30 mL), dried over Na_2_SO_4_ and evaporated to dryness. The crude product was purified by flash column chromatography to afford the corresponding product **21**, **23**, **25**, **27–35** and **37–41**.

##### 2-(1-Ethyl-6-fluoro-8-phenyl-1,3,4,9-tetrahydropyrano­[3,4-*b*]­indol-1-yl)­acetic Acid (**21**)

The reaction was run with **97a** (0.068 g, 0.19 mmol), LiOH 3 M (0.317 mL, 0.95 mmol), 1,4-dioxane (1.5 mL). After purification by flash column chromatography (silica gel, DCM/MeOH 95:5) the product **21** was obtained as a white solid (62 mg, 92% yield). ^1^H NMR (300 MHz, (CD_3_)_2_CO): δ 10.02 (s, 1H, NH), 7.62 (d, *J* = 7.0 Hz, 2H, ArH_2′‑6′_), 7.48 (t, *J* = 7.4 Hz, 2H, ArH_3′‑5′_), 7.38 (d, *J* = 6.6 Hz, 1H, ArH_4′_), 7.14 (d, *J* = 8.9 Hz, 1H, ArH_7_), 6.91 (d, *J* = 10.0 Hz, 1H, ArH_5_), 3.99 (d, *J* = 4.3 Hz, 2H, OCH_2_−), 2.95 and 2.91 (two signals, d, *J* = 15.6 Hz, 2H, *CH*_2_COOH), 2.77–2.66 (m, 2H, CH_2_), 1.16–1.10 (m, 2H, C*H*_2_CH_3_), 0.73 (t, *J* = 6.9 Hz, 3H, CH_3_). ^13^C NMR (75 MHz, (CD_3_)_2_CO): δ 209.83, 158.72 (d, *J*_C–F_ = 232.5 Hz), 139.92, 138.87, 130.56 (d, *J*_C–F_ = 21.7 Hz), 129.73, 128.81, 128.35, 126.90 (d, *J*_C–F_ = 8.7 Hz), 109.62 (d, *J*_C–F_ = 26.4 Hz), 109.02, 102.75 (d, *J*_C–F_ = 23.3 Hz), 75.50, 69.06, 60.71, 42.98, 29.62, 22.62, 7.79. MS (ESI^–^) *m*/*z*: 352 [M – H]^−^. HRMS (ESI-TOF) *m*/*z*: [M – H]^−^ calcd for C_21_H_19_FNO_3_^–^, 352.1354; found, 352.1357.

##### 2-(8-(2-Chlorophenyl)-1-ethyl-6-fluoro-1,3,4,9-tetrahydropyrano­[3,4-*b*]­indol-1-yl)­acetic Acid (**23**)

The reaction was run with **97b** (0.058 g, 0.14 mmol), LiOH 3 M (0.235 mL, 0.7 mmol), 1,4-dioxane (1 mL). After purification by flash column chromatography (silica gel, PE/EtOAc/MeOH 8:1.5:0.5) the product **23** was obtained as a white solid (54 mg, 100% yield). ^1^H NMR (600 MHz, DMSO-*d*_6_): δ 12.01 (s, 1H, COOH), 10.41 (s, 1H, NH), 7.62 (d, 7.2 Hz, 1H, ArH_6′_), 7.45–7.41 (m, 3H, ArH_3′‑4′‑5′_), 7.21 (d, *J* = 9.2 Hz, 1H, ArH_7_), 6.76 (d, *J* = 9.2 Hz, 1H, ArH_5_), 3.92–3.86 (m, 2H, OCH_2_−), 2.86 and 2.68 (two signals, d, *J* = 13.8 Hz, 2H, *CH*_2_COOH), 2.62–2.55 (m, 2H, CH_2_), 1.97–1.90 (m, 2H, C*H*_2_CH_3_), 0.61 (t, *J* = 6.8 Hz, 3H, CH_3_). ^13^C NMR (151 MHz, DMSO-*d*_6_): δ 171.53, 156.30 (d, *J*_C–F_ = 231.3 Hz), 139.61, 136.45, 132.75, 131.93, 130.30, 129.81, 129.66, 127.43, 126.80, 123.40, 109.76 (d, *J*_C–F_ = 29.7 Hz), 107.53, 102.56 (d, *J*_C–F_ = 22.5 Hz), 75.27, 59.77, 42.45, 30.38, 21.81, 7.77. MS (ESI^–^) *m*/*z*: 386/388 [M – H]^−^.

##### 2-(8-(3-Chlorophenyl)-1-ethyl-6-fluoro-1,3,4,9-tetrahydropyrano­[3,4-*b*]­indol-1-yl)­acetic Acid (**25**)

The reaction was run with **97c** (0.107 g, 0.27 mmol), LiOH 3 M (0.450 mL, 1.35 mmol), 1,4-dioxane (2 mL). After purification by flash column chromatography (silica gel, PE/EtOAc/MeOH 8:1.5:0.5) the product **25** was obtained as a yellow solid (104 mg, 99% yield). ^1^H NMR (300 MHz, DMSO-*d*_6_): δ 10.73 (s, 1H, NH), 7.57–7.28 (m, 4H, ArH_2′‑4′‑5′‑6′_), 7.08 (d, *J* = 8.0 Hz, 1H, ArH_7_), 6.81 (d, *J* = 9.2 Hz, 1H, ArH_5_), 3.85–3.76 (m, 2H, OCH_2_−), 2.73 and 2.61 (two signals, d, *J* = 13.4 Hz, 2H, *CH*_2_COOH), 2.51–2.50 (m, 2H, CH_2_), 2.02–1.77 (m, 2H, C*H*_2_CH_3_), 0.52 (t, 3H, CH_3_). ^13^C NMR (151 MHz, DMSO-*d*_6_): δ 172.73, 157.26 (d, *J*_C–F_ = 232.0 Hz), 140.85, 140.33, 134.16, 131.28, 130.04, 128.69, 128.12, 128.02, 127.60, 124.87 (d, *J*_C–F_ = 10.1 Hz), 109.43 (d, *J*_C–F_ = 26.8 Hz), 108.23, 103.10 (d, *J*_C–F_ = 22.8 Hz), 75.66, 60.17, 43.53, 30.80, 22.32, 8.28. MS (ESI^–^) *m*/*z*: 386/388 [M – H]^−^. HRMS (ESI-TOF) *m*/*z*: [M – H]^−^ calcd for C_21_H_18_ClFNO_3_^–^, 386.0965; found, 386.0969.

##### 2-(8-(4-Chlorophenyl)-1-ethyl-6-fluoro-1,3,4,9-tetrahydropyrano­[3,4-*b*]­indol-1-yl)­acetic Acid (**27**)

The reaction was run with **97d** (0.117 g, 0.29 mmol), LiOH 3 M (0.485 mL, 1.45 mmol), 1,4-dioxane (2 mL). After purification by flash column chromatography (silica gel, PE/EtOAc/MeOH 8:1.5:0.5) the product **27** was obtained as a white solid (102 mg, 91% yield). ^1^H NMR (600 MHz, DMSO-*d*_6_): δ 12.02 (s, 1H, COOH), 10.63 (s, 1H, NH), 7.62–7.60 (m, 2H, ArH_2′‑6′_), 7.58–7.56 (m, 2H, ArH_3′–5′_), 7.22 (d, *J* = 9.7 Hz, 1H, ArH_7_), 6.92 (d, *J* = 9.7 Hz, 1H, ArH_5_), 3.99–3.86 (m, 2H, OCH_2_−), 2.90 and 2.77 (two signals, d, *J* = 14.0 Hz, 2H, *CH*_2_COOH), 2.70–2.66 (m, 2H, CH_2_), 2.08–1.95 (m, 2H, C*H*_2_CH_3_), 0.65 (t, *J* = 7.3 Hz, 3H, CH_3_). ^13^C NMR (151 MHz, DMSO-*d*_6_): δ 171.80, 156.98 (d, *J*_C–F_ = 232.4 Hz), 139.92, 136.52, 132.46, 130.30, 129.67, 128.93, 127.51 (d, *J*_C–F_ = 10.1 Hz), 124.66 (d, *J*_C–F_ = 10.1 Hz), 108.80 (d, *J*_C–F_ = 26.2 Hz), 107.93 (d, *J*_C–F_ = 4.3 Hz), 102.33 (d, *J*_C–F_ = 22.8 Hz), 75.24, 59.73, 42.59, 30.37, 21.80, 7.79. MS (ESI^–^) *m*/*z*: 386/388 [M – H]^−^.

##### 2-(1-Ethyl-6-fluoro-8-(2-hydroxyphenyl)-1,3,4,9-tetrahydropyrano­[3,4-*b*]­indol-1-yl)­acetic Acid (**28**)

The reaction was run with **97e** (0.130 g, 0.34 mmol), LiOH 3 M (0.567 mL, 1.7 mmol), 1,4-dioxane (2 mL). After purification by flash column chromatography (silica gel, PE/EtOAc/MeOH 7:2:1) the product **28** was obtained as a white solid (94 mg, 75% yield). ^1^H NMR (600 MHz, DMSO-*d*_6_): δ 12.06 (s, 1H, COOH), 10.08 (s, 1H, NH), 9.61 (s, 1H, OH), 7.31–7.18 (m, 2H, ArH_2′‑3′_), 7.11 (d, *J* = 9.3, Hz, 1H, ArH_4′_), 6.98 (d, *J* = 8.1 Hz, 1H, ArH_5′_), 6.88 (t, *J* = 7.4 Hz, 1H, ArH_7_), 6.78 (d, *J* = 10.3 Hz, 1H, ArH_5_), 4.00–3.83 (m, 2H, OCH_2_−), 2.88 and 2.78 (two signals, d, *J* = 14.2 Hz, 2H, *CH*_2_COOH), 2.70–2.54 (m, 2H, CH_2_), 2.10–1.86 (m, 2H, C*H*_2_CH_3_), 0.60 (t, *J* = 7.2 Hz, 3H, CH_3_). ^13^C NMR (151 MHz, DMSO-*d*_6_): δ 172.38, 157.06 (d, *J*_C–F_ = 231.2 Hz), 155.39, 139.41, 131.50, 131.07, 129.40, 125.98 (d, *J*_C–F_ = 10.1 Hz), 124.29, 123.23 (d, *J*_C–F_ = 8.7 Hz), 119.70, 116.56, 110.40 (d, *J*_C–F_ = 25.7 Hz), 107.64 (d, *J*_C–F_ = 4.3 Hz), 101.85 (d, *J*_C–F_ = 22.4 Hz), 75.70, 60.27, 42.87, 30.80, 22.38, 8.31. MS (ESI^–^) *m*/*z*: 368 [M – H]^−^.

##### 2-(1-Ethyl-6-fluoro-8-(3-hydroxyphenyl)-1,3,4,9-tetrahydropyrano­[3,4-*b*]­indol-1-yl)­acetic Acid (**29**)

The reaction was run with **97f** (0.145 g, 0.38 mmol), LiOH 3 M (0.635 mL, 1.9 mmol), 1,4-dioxane (2.5 mL). After purification by flash column chromatography (silica gel, PE/EtOAc/MeOH 7:2:1) the product **29** was obtained as an off-white solid (128 mg, 91% yield). ^1^H NMR (600 MHz, DMSO-*d*_6_): δ 10.76 (s, 1H, NH), 7.27 (t, *J* = 7.8 Hz, 1H, ArH_2′_), 7.13 (dd, *J* = 9.3, 2.4 Hz, 1H, ArH_4′_), 7.04–6.93 (m, 2H, ArH_5′‑6′_), 6.85–6.81 (m, 2H, ArH_5–7_), 3.95–3.87 (m, 2H, OCH_2_−), 2.85 and 2.78 (two signals, d, *J* = 14.5 Hz, 2H, *CH*_2_COOH), 2.67–2.54 (m, 2H, CH_2_), 2.07–1.89 (m, 2H, C*H*_2_CH_3_), 0.63 (t, *J* = 7.3 Hz, 3H, CH_3_). ^13^C NMR (151 MHz, DMSO-*d*_6_): δ 172.00, 158.32, 157.45 (d, *J*_C–F_ = 232.1 Hz), 140.66, 139.54, 130.49, 130.04, 127.82 (d, *J*_C–F_ = 10.1 Hz), 126.70 (d, *J*_C–F_ = 10.1 Hz), 119.43, 115.68, 115.27, 108.91 (d, *J*_C–F_ = 26.2 Hz), 107.99, 102.27 (d, *J*_C–F_ = 23.1 Hz), 75.20, 60.13, 42.05, 30.69, 22.36, 8.30. MS (ESI^–^) *m*/*z*: 368 [M – H]^−^.

##### 2-(1-Ethyl-6-fluoro-8-(4-hydroxyphenyl)-1,3,4,9-tetrahydropyrano­[3,4-*b*]­indol-1-yl)­acetic Acid (**30**)

The reaction was run with **97g** (0.100 g, 0.26 mmol), LiOH 3 M (0.435 mL, 1.30 mmol), 1,4-dioxane (2 mL). After purification by flash column chromatography (silica gel, DCM/MeOH 95:5) the product **30** was obtained as a white solid (76 mg, 80% yield). ^1^H NMR (600 MHz, DMSO-*d*_6_): δ 12.06 (s, 1H, COOH), 10.39 (s, 1H, NH), 9.63 (s, 1H, OH), 7.40 (d, *J* = 8.3 Hz, 2H, H_2′‑6′_), 7.08 (dd, *J* = 9.2, 1.9 Hz, 1H, ArH_7_), 6.90 (d, *J* = 8.3 Hz, 2H, H_3′–5′_), 6.79 (d, *J* = 10.3 Hz, 1H, ArH_5_), 3.95–3.84 (m, 2H, OCH_2_−), 2.91 and 2.82 (two signals, d, *J* = 14.1 Hz, 2H, *CH*_2_COOH), 2.63–2.45 (m, 2H, CH_2_), 2.09–1.95 (m, 2H, C*H*_2_CH_3_), 0.62 (t, *J* = 7.1 Hz, 3H, CH_3_). ^13^C NMR (151 MHz, DMSO-*d*_6_): δ 172.02, 157.24, 156.35 (d, *J*_C–F_ = 232.1 Hz), 139.42, 129.71, 129.53, 128.38, 127.24 (d, *J*_C–F_ = 10.1 Hz), 126.37 (d, *J*_C–F_ = 8.7 Hz), 115.77, 108.35 (d, *J*_C–F_ = 25.4 Hz), 107.82 (d, *J*_C–F_ = 4.3 Hz), 101.04 (d, *J*_C–F_ = 23.0 Hz), 75.34, 59.76, 42.44, 30.36, 21.85, 7.91. MS (ESI^–^) *m*/*z*: 368 [M – H]^−^.

##### 2-(1-Ethyl-6-fluoro-8-(2-(hydroxymethyl)­phenyl)-1,3,4,9-tetrahydropyrano­[3,4-*b*]­indol-1-yl)­acetic Acid (**31**)

The reaction was run with **97h** (0.127 g, 0.32 mmol), LiOH 3 M (0.533 mL, 1.6 mmol), 1,4-dioxane (2 mL). After purification by flash column chromatography (silica gel, PE/EtOAc/MeOH 7:2:1) the product **31** was obtained as a yellow solid (91 mg, 74% yield). ^1^H NMR (600 MHz, DMSO-*d*_6_): δ 10.36 (s, 1H, NH), 7.67 (d, *J* = 7.6 Hz, 1H, ArH_3′_), 7.47 (t, *J* = 7.4 Hz, 1H, ArH_4′_), 7.37 (t, *J* = 7.4 Hz, 1H, ArH_5′_), 7.28 (d, *J* = 7.2 Hz, 1H, ArH_6′_), 7.20 (dd, *J* = 9.5, 2.3 Hz, 1H, ArH_7_), 6.75 (dd, *J* = 10.0, 2.2 Hz, 1H, ArH_5_), 5.06 (s, 1H, OH), 4.09–4.15 (m, 2H, *CH*_2_OH), 3.96–3.87 (m, 2H, OCH_2_−), 2.84 and 2.69 (two signals, d, *J* = 14.0 Hz, 2H, *CH*_2_COOH), 2.74–2.60 (m, 2H, CH_2_), 2.06–1.94 (m, 2H, C*H*_2_CH_3_), 0.56 (t, *J* = 7.4 Hz, 3H, CH_3_). ^13^C NMR (151 MHz, DMSO-*d*_6_): δ 172.23, 156.21 (d, *J*_C–F_ = 232.3 Hz), 141.10, 140.16, 135.99, 131.15, 130.09, 129.44, 129.22, 128.33, 127.98, 126.85, 110.26 (d, *J*_C–F_ = 26.0 Hz), 108.09 (d, *J*_C–F_ = 4.3 Hz), 102.28 (d, *J*_C–F_ = 23.1 Hz), 75.74, 60.88, 60.24, 43.40, 30.86, 22.35, 8.27. MS (ESI^–^) *m*/*z*: 382 [M – H]^−^.

##### 2-(1-Ethyl-6-fluoro-8-(3-(hydroxymethyl)­phenyl)-1,3,4,9-tetrahydropyrano­[3,4-*b*]­indol-1-yl)­acetic Acid (**32**)

The reaction was run with **97i** (0.200 g, 0.50 mmol), LiOH 3 M (0.835 mL, 2.50 mmol), 1,4-dioxane (3 mL). After purification by flash column chromatography (silica gel, PE/EtOAc/MeOH 7:2:1) the product **32** was obtained as a beige solid (180 mg, 94% yield). ^1^H NMR (600 MHz, DMSO-*d*_6_): δ 12.09 (s, 1H, COOH), 10.46 (s, 1H, NH), 7.52–7.48 (m, 3H, ArH_4′‑5′‑6′_), 7.41 (s, 1H, ArH_2′_), 7.18 (d, *J* = 9.3 Hz, 1H, ArH_7_), 6.88 (d, *J* = 10.3 Hz, 1H, ArH_5_), 5.24 (s, 1H, OH), 4.60 (s, 2H, *CH*_2_OH), 3.96–3.88 (m, 2H, OCH_2_−), 2.92 and 2.81 (two signals, d, *J* = 14.1 Hz, 2H, *CH*_2_COOH), 2.70–2.61 (m, 2H, CH_2_), 2.08–1.94 (m, 2H, C*H*_2_CH_3_), 0.64 (t, *J* = 7.3 Hz, 3H, CH_3_). ^13^C NMR (151 MHz, DMSO-*d*_6_): δ 171.91, 157.04 (d, *J*_C–F_ = 231.9 Hz), 143.32, 139.62, 137.46, 129.72, 128.74, 127.41 (d, *J*_C–F_ = 10.1 Hz), 126.64, 126.53, 126.23 (d, *J*_C–F_ = 10.1 Hz), 125.77, 108.75 (d, *J*_C–F_ = 26.1 Hz), 107.89 (d, *J*_C–F_ = 5.8 Hz), 101.88 (d, *J*_C–F_ = 22.4 Hz), 75.21, 62.90, 59.74, 42.40, 30.38, 21.82, 7.77. MS (ESI^–^) *m*/*z*: 382 [M – H]^−^. HRMS (ESI-TOF) *m*/*z*: [M – H]^−^ calcd for C_22_H_21_FNO_4_^–^, 382.1460; found, 382.1464.

##### 2-(1-Ethyl-6-fluoro-8-(4-(hydroxymethyl)­phenyl)-1,3,4,9-tetrahydropyrano­[3,4-*b*]­indol-1-yl)­acetic Acid (**33**)

The reaction was run with **97j** (0.125 g, 0.31 mmol), LiOH 3 M (0.517 mL, 1.55 mmol), 1,4-dioxane (2 mL). After purification by flash column chromatography (silica gel, PE/EtOAc/MeOH 7:2:1) the product **33** was obtained as a yellow solid (99 mg, 83% yield). ^1^H NMR (600 MHz, DMSO-*d*_6_): δ 10.95 (s, 1H, NH), 7.55 (d, *J* = 8.1 Hz, 2H, ArH_2′‑6′_), 7.43 (d, *J* = 8.1 Hz, 2H, ArH_3′–5′_), 7.14 (dd, *J* = 9.3, 2.4 Hz, 1H, ArH_7_), 6.87 (dd, *J* = 10.3, 2.4 Hz, 1H, ArH_5_), 5.32 (s, 1H, OH), 4.54 (s, 2H, *CH*_2_OH), 3.92–3.80 (m, 2H, OCH_2_−), 2.82 and 2.76 (two signals, d, *J* = 14.5 Hz, 2H, *CH*_2_COOH), 2.68–2.55 (m, 2H, CH_2_), 2.04–1.89 (m, 2H, C*H*_2_CH_3_), 0.64 (t, *J* = 7.3 Hz, 3H, CH_3_). ^13^C NMR (151 MHz, DMSO-*d*_6_): δ 172.61, 156.46 (d, *J*_C–F_ = 232.1 Hz), 142.54, 140.94, 136.61, 130.07, 128.51, 127.96 (d, *J*_C–F_ = 10.1 Hz), 127.58, 126.42 (d, *J*_C–F_ = 8.7 Hz), 108.94 (d, *J*_C–F_ = 27.5 Hz), 107.85 (d, *J*_C–F_ = 2.9 Hz), 102.32 (d, *J*_C–F_ = 23.1 Hz), 75.55, 63.18, 60.11, 43.88, 30.65, 22.39, 8.29. MS (ESI^–^) *m*/*z*: 382 [M – H]^−^.

##### 2-(8-(3-Acetamidophenyl)-1-ethyl-6-fluoro-1,3,4,9-tetrahydropyrano­[3,4-*b*]­indol-1-yl)­acetic Acid (**34**)

The reaction was run with **97k** (0.192 g, 0.45 mmol), LiOH 3 M (0.750 mL, 2.25 mmol), 1,4-dioxane (3 mL). After purification by flash column chromatography (silica gel, DCM/MeOH 95:5) the product **34** was obtained as a light-brown solid (161 mg, 87% yield). ^1^H NMR (600 MHz, DMSO-*d*_6_): δ 12.20 (s, 1H, COOH), 10.47 (s, 1H, NH), 10.04 (s, 1H, N*H*–CO), 7.76 (s, 1H, ArH_2′_), 7.71–7.66 (m, 1H, ArH_4′_), 7.41 (t, *J* = 7.9 Hz, 1H, ArH_5′_), 7.23 (d, *J* = 7.7 Hz, 1H, ArH_6′_), 7.16 (dd, *J* = 9.3, 2.4 Hz, 1H, ArH_7_), 6.81 (dd, *J* = 10.2, 2.4 Hz, 1H, ArH_5_), 3.95–3.82 (m, 2H, OCH_2_−), 2.91 and 2.80 (two signals, d, *J* = 14.1 Hz, 2H, *CH*_2_COOH), 2.71–2.53 (m, 2H, CH_2_), 2.07 (s, 3H, NHCOC*H*_3_), 2.06–1.95 (m, 2H, *CH*_2_CH_3_), 0.62 (t, *J* = 7.3 Hz, 3H, CH_3_). ^13^C NMR (151 MHz, DMSO-*d*_6_): δ 172.36, 168.98, 156.96 (d, *J*_C–F_ = 232.2 Hz), 140.41, 140.22, 138.64, 130.21, 129.77, 127.83 (d, *J*_C–F_ = 10.1 Hz), 126.54 (d, *J*_C–F_ = 8.7 Hz), 123.53, 119.46, 118.75, 108.62 (d, *J*_C–F_ = 25.7 Hz), 108.40 (d, *J*_C–F_ = 4.3 Hz), 101.97 (d, *J*_C–F_ = 22.8 Hz), 75.74, 60.23, 42.88, 30.83, 24.65, 22.33, 8.30. MS (ESI^–^) *m*/*z*: 409 [M – H]^−^.

##### 2-(8-(3-Carbamoylphenyl)-1-ethyl-6-fluoro-1,3,4,9-tetrahydropyrano­[3,4-*b*]­indol-1-yl)­acetic Acid (**35**)

The reaction was run with **97l** (0.060 g, 0.15 mmol), LiOH 3 M (0.250 mL, 0.75 mmol), 1,4-dioxane (2 mL). After purification by flash column chromatography (silica gel, DCM/MeOH 9:1) the product **35** was obtained as a white solid (58 mg, 98% yield). ^1^H NMR (600 MHz, DMSO-*d*_6_): δ 11.82 (s, 1H, COOH), 8.56 (s, 1H, NH), 8.06 (s, 1H, ArH_2′_), 7.85 (d, *J* = 7.8 Hz, 1H, ArH_4′_), 7.75 (d, *J* = 7.7 Hz, 1H, ArH_6′_), 7.58 (t, *J* = 7.7 Hz, 1H, ArH_5′_), 7.32 (s, 2H, CON*H*_2_), 7.18 (dd, *J* = 9.4, 2.4 Hz, 1H, ArH_7_), 7.00 (dd, *J* = 10.4, 2.4 Hz, 1H, ArH_5_), 3.94–3.82 (m, 2H, OCH_2_−), 2.78–2.58 (m, 4H, CH_2_ and *CH*_2_COOH), 2.05–1.84 (m, 2H, C*H*_2_CH_3_), 0.68 (t, *J* = 7.3 Hz, 3H, CH_3_). ^13^C NMR (151 MHz, DMSO-*d*_6_): δ 173.65, 168.80, 157.55 (d, *J*_C–F_ = 231.42 Hz), 142.08, 138.15, 136.00, 131.45, 129.94, 129.70, 128.14 (d, *J*_C–F_ = 10.1 Hz), 127.60, 127.27, 125.48 (d, *J*_C–F_ = 10.1 Hz), 108.82 (d, *J*_C–F_ = 27.1 Hz), 107.34 (d, *J*_C–F_ = 7.2 Hz), 102.84 (d, *J*_C–F_ = 22.5 Hz), 75.38, 60.09, 40.56, 30.54, 22.47, 8.33. MS (ESI^–^) *m*/*z*: 395 [M – H]^−^.

##### 2-(8-(3-Acetylphenyl)-1-ethyl-6-fluoro-1,3,4,9-tetrahydropyrano­[3,4-*b*]­indol-1-yl)­acetic Acid (**37**)

The reaction was run with **97n** (0.077 g, 0.19 mmol), LiOH 3 M (0.315 mL, 0.95 mmol), 1,4-dioxane (1 mL). After purification by flash column chromatography (silica gel, DCM/MeOH 98:2) the product **37** was obtained as a pale-yellow solid (46 mg, 61% yield). ^1^H NMR (600 MHz, CD_3_CN): δ 9.28 (s, 1H, NH), 8.10 (s, 1H, ArH_2′_), 7.92 (d, *J* = 7.9 Hz, 1H, ArH_4′_), 7.76 (d, *J* = 7.6 Hz, 1H, ArH_6′_), 7.55 (t, *J* = 7.7 Hz, 1H, ArH_5′_), 7.11 (dd, *J* = 9.8, 2.2 Hz, 1H, ArH_7_), 6.92 (dd, *J* = 10.0, 2.4 Hz, 1H, ArH_5_), 3.98–3.84 (m, 2H, OCH_2_−), 2.86–2.74 (m, 2H, *CH*_2_COOH), 2.65 (m, 2H, CH_2_), 2.54 (s, 3H, COC*H*_3_), 2.08–1.90 (m, 2H, C*H*_2_CH_3_), 0.65 (t, *J* = 7.3 Hz, 3H, CH_3_). ^13^C NMR (151 MHz, CD_3_CN): δ 199.09, 172.98, 158.72 (d, *J*_C–F_ = 232.6 Hz), 139.93, 139.24, 138.86, 133.83, 130.99, 130.50, 129.26, 128.53 (d, *J*_C–F_ = 10.1 Hz), 128.22, 126.37 (d, *J*_C–F_ = 10.1 Hz), 110.28 (d, *J*_C–F_ = 27.0 Hz), 109.62 (d, *J*_C–F_ = 4.3 Hz), 103.53 (d, *J*_C–F_ = 23.3 Hz), 75.94, 61.11, 42.98, 31.34, 27.18, 22.60, 7.84. MS (ESI^–^) *m*/*z*: 394 [M – H]^−^.

##### 2-(1-Ethyl-6-fluoro-8-(3-(methylsulfonyl)­phenyl)-1,3,4,9-tetrahydropyrano­[3,4-*b*]­indol-1-yl)­acetic Acid (**38**)

The reaction was run with **97o** (0.102 g, 0.23 mmol), LiOH 3 M (0.385 mL, 1.15 mmol), 1,4-dioxane (2 mL). After purification by flash column chromatography (silica gel, PE/EtOAc/MeOH 8:1.5:0.5) the product **38** was obtained as a white solid (71 mg, 72% yield). ^1^H NMR (600 MHz, DMSO-*d*_6_): δ 12.09 (s, 1H, COOH), 10.69 (s, 1H, NH), 8.09 (s, 1H, ArH_2′_), 7.99–7.96 (m, 2H, ArH_4′‑6′_), 7.83 (t, *J* = 7.7 Hz, 1H, ArH_5′_), 7.28 (dd, *J* = 9.7, 2.4 Hz, 1H, ArH_7_), 7.08 (dd, *J* = 10.3, 2.4 Hz, 1H, ArH_5_), 3.97–3.89 (m, 2H, OCH_2_−), 3.32 (s, 3H, SO_2_C*H*_3_), 2.90 and 2.75 (two signals, d, *J* = 14.0 Hz, 2H, *CH*_2_COOH), 2.71–2.62 (m, 2H, CH_2_), 2.06–1.94 (m, 2H, C*H*_2_CH_3_), 0.67 (t, *J* = 7.3 Hz, 3H, CH_3_). ^13^C NMR (151 MHz, DMSO-*d*_6_): δ 171.67, 157.03 (d, *J*_C–F_ = 232.3 Hz), 141.59, 140.04, 138.83, 133.45, 130.11, 129.73, 127.72 (d, *J*_C–F_ = 10.1 Hz), 126.98, 126.01, 124.21 (d, *J*_C–F_ = 10.1 Hz), 109.27 (d, *J*_C–F_ = 26.5 Hz), 108.09 (d, *J*_C–F_ = 4.3 Hz), 102.90 (d, *J*_C–F_ = 22.6 Hz), 75.24, 59.74, 43.53, 42.50, 30.48, 21.79, 7.81. MS (ESI^–^) *m*/*z*: 430 [M – H]^−^.

##### 2-(1-Ethyl-6-fluoro-8-(3-methoxyphenyl)-1,3,4,9-tetrahydropyrano­[3,4-*b*]­indol-1-yl)­acetic Acid (**39**)

The reaction was run with **97p** (0.108 g, 0.27 mmol), LiOH 3 M (0.450 mL, 1.35 mmol), 1,4-dioxane (2 mL). After purification by flash column chromatography (silica gel, PE/EtOAc/MeOH 8:1.5:0.5) the product **39** was obtained as a white solid (55 mg, 53% yield). ^1^H NMR (600 MHz, CD_3_CN): δ 9.27 (s, 1H, NH), 7.33 (t, *J* = 7.9 Hz, 1H, ArH_5′_), 7.19 (d, *J* = 7.6 Hz, 1H, ArH_6′_), 7.17 (dd, *J* = 10.0, 2.4 Hz, 2H, ArH_7–5_), 7.00–6.97 (m, 2H, ArH_2′‑4′_), 3.96–3.84 (m, 2H, OC*H*_2_–), 3.76 (s, 3H, OC*H*_3_), 2.86–2.76 (m, 2H, C*H*_2_COOH), 2.76–2.68 (m, 2H, CH_2_), 2.07–1.91 (m, 2H, C*H*_2_CH_3_), 0.65 (t, *J* = 7.4 Hz, 3H, CH_3_). ^13^C NMR (151 MHz, CD_3_CN): δ 173.04, 161.08, 158.68 (d, *J*_C–F_ = 232.2 Hz), 140.23, 139.71, 131.21, 130.90, 128.42 (d, *J*_C–F_ = 10.1 Hz), 127.17 (d, *J*_C–F_ = 8.7 Hz), 121.46, 114.73, 114.22, 110.08 (d, *J*_C–F_ = 27.0 Hz), 109.46 (d, *J*_C–F_ = 4.3 Hz), 103.11 (d, *J*_C–F_ = 23.5 Hz), 75.89, 61.09, 55.88, 42.88, 31.27, 22.60, 7.84. MS (ESI^–^) *m*/*z*: 382 [M – H]^−^.

##### 2-(8-(2,5-Dichlorophenyl)-1-ethyl-6-fluoro-1,3,4,9-tetrahydropyrano­[3,4-*b*]­indol-1-yl)­acetic Acid (**40**)

The reaction was run with **97q** (0.117 g, 0.27 mmol), LiOH 3 M (0.450 mL, 1.35 mmol), 1,4-dioxane (2 mL). After purification by flash column chromatography (silica gel, DCM/MeOH 98:2) the product **40** was obtained as a white solid (106 mg, 93% yield). ^1^H NMR (600 MHz, DMSO-*d*_6_): δ 12.09 (s, 1H, COOH), 10.50 (s, 1H, NH), 7.61 (d, *J* = 8.6 Hz, 1H, ArH_3′_), 7.52 (dd, *J* = 8.6, 2.0 Hz, 1H, ArH_4′_), 7.49 (d, *J* = 1.9 Hz, 1H, ArH_6′_), 7.26 (d, *J* = 9.3 Hz, 1H, ArH_7_), 6.83 (d, *J* = 9.6 Hz, 1H, ArH_5_), 3.97–3.83 (m, 2H, OCH_2_−), 3.28–2.79 (m, 2H, *CH*_2_COOH), 2.70–2.43 (m, 2H, CH_2_), 1.93 (d, *J* = 7.2 Hz, 2H, C*H*_2_CH_3_), 0.57 (t, *J* = 7.1 Hz, 3H, CH_3_). ^13^C NMR (151 MHz, DMSO-*d*_6_): δ 171.59, 156.26 (d, *J*_C–F_ = 232.3 Hz), 139.85, 138.27, 131.77, 131.42, 131.37, 130.12, 129.50, 126.84, 122.07 (d, *J*_C–F_ = 11.6 Hz), 117.26, 109.67 (d, *J*_C–F_ = 27.7 Hz), 107.58 (d, *J*_C–F_ = 4.3 Hz), 103.09 (d, *J*_C–F_ = 22.8 Hz), 75.27, 59.77, 42.66, 30.49, 21.81, 7.78. MS (ESI^–^) *m*/*z*: 420/421/422 [M – H]^−^.

##### 2-(8-(3,5-Dichlorophenyl)-1-ethyl-6-fluoro-1,3,4,9-tetrahydropyrano­[3,4-*b*]­indol-1-yl)­acetic Acid (**41**)

The reaction was run with **97r** (0.123 g, 0.28 mmol), LiOH 3 M (0.470 mL, 1.41 mmol), 1,4-dioxane (2 mL). After purification by flash column chromatography (silica gel, PE/EtOAc/MeOH 8:1.5:0.5) the product **41** was obtained as a white solid (49 mg, 41% yield). ^1^H NMR (600 MHz, DMSO-*d*_6_): δ 11.98 (s, 1H, COOH), 10.63 (s, 1H, NH), 7.67 (s, 1H, ArH_4′_), 7.57 (d, *J* = 1.5 Hz, 2H, ArH_2′‑6′_), 7.24 (dd, *J* = 9.2, 2.0 Hz, 1H, ArH_7_), 6.96 (dd, *J* = 10.0, 2.1 Hz, 1H, ArH_5_), 3.95–3.85 (m, 2H, OCH_2_−), 2.92 and 2.75 (two signals, d, *J* = 13.8 Hz, 2H, *CH*_2_COOH), 2.69–2.58 (m, 2H, CH_2_), 2.05–1.92 (m, 2H, C*H*_2_CH_3_), 0.63 (t, *J* = 7.3 Hz, 3H, CH_3_). ^13^C NMR (151 MHz, DMSO-*d*_6_): δ 171.26, 156.57 (d, *J*_C–F_ = 232.7 Hz), 140.86, 139.71, 134.23, 129.31, 127.35 (d, *J*_C–F_ = 10.1 Hz), 127.11, 127.01, 122.74 (d, *J*_C–F_ = 10.1 Hz), 109.05 (d, *J*_C–F_ = 26.4 Hz), 107.88 (d, *J*_C–F_ = 4.3 Hz), 102.91 (d, *J*_C–F_ = 22.8 Hz), 75.02, 59.47, 42.16, 30.22, 21.48, 7.49. MS (ESI^–^) *m*/*z*: 420/421/422 [M – H]^−^.

##### 2-(8-(3-(Aminomethyl)­phenyl)-1-ethyl-6-fluoro-1,3,4,9-tetrahydropyrano­[3,4-*b*]­indol-1-yl)­acetic Acid (**36**)

To a solution of compound **97m** (0.200 g, 0.41 mmol) in DCM (3 mL), TFA (0.89 mL, 12 mmol) was added, and the solution was stirred at RT for 45 min. The solvent was evaporated and an aqueous solution of K_2_CO_3_ was added. The mixture was extracted with EtOAc (3 × 20 mL), the organic extracts were washed with brine, dried over Na_2_SO_4_ and evaporated to dryness. The crude product was purified by flash column chromatography (silica gel, DCM/MeOH 95:5) and the ester derivative was obtained as a yellow solid (109 mg, 68% yield). MS (ESI^+^) *m*/*z*: 397 [M + H]^+^.

To a solution of the ester derivative (0.109 g, 0.27 mmol, 1 equiv) in 1,4-dioxane (2 mL), an aqueous solution of LiOH 3 M (0.450 mL, 1.35 mmol, 5 equiv) was added and the mixture was stirred at RT for 16 h. The reaction was quenched by adding a solution of phosphate buffer (pH 7.4) and the mixture was extracted with EtOAc (3 × 20 mL). The extracts were washed with brine (30 mL), dried over Na_2_SO_4_ and evaporated to dryness. The crude product was purified by flash column chromatography (silica gel, DCM/MeOH 9:1) to afford **36** as a white solid (98 mg, 95% yield). ^1^H NMR (600 MHz, DMSO-*d*_6_ + HCl): δ 10.61 (s, 1H, NH), 8.70 (s, 3H, NH_3_), 7.76 (s, 1H, ArH_2′_), 7.57 (dd, *J* = 3.8, 2.3 Hz, 1H, ArH_4′_),7.53–7.47 (m, 2H, ArH_5′‑6′_), 7.17 (dd, *J* = 9.2, 2.4 Hz, 1H, ArH_7_), 6.92 (dd, *J* = 10.3, 2.4 Hz, 1H, ArH_5_), 4.04 (q, *J* = 5.6 Hz, 2H, C*H*_2_NH_2_), 3.93–3.83 (m, 2H, OCH_2_−), 3.02 and 2.93 (two signals, d, *J* = 14.1 Hz, 2H, *CH*_2_COOH), 2.66–2.53 (m, 2H, CH_2_), 2.18–1.96 (m, 2H, C*H*_2_CH_3_), 0.58 (t, *J* = 7.3 Hz, 3H, CH_3_). ^13^C NMR (151 MHz, DMSO-*d*_6_ + HCl): δ 171.81, 157.07 (d, *J*_C–F_ = 232.2 Hz), 139.98, 137.83, 134.49, 129.70, 129.61, 129.27, 128.67, 128.46, 127.65 (d, *J*_C–F_ = 10.1 Hz), 125.50 (d, *J*_C–F_ = 10.1 Hz), 109.01 (d, *J*_C–F_ = 26.1 Hz), 108.00 (d, *J*_C–F_ = 5.8 Hz), 102.20 (d, *J*_C–F_ = 23.1 Hz), 75.45, 59.81, 42.55, 42.36, 30.39, 21.89, 7.89. MS (ESI^–^) *m*/*z*: 381 [M – H]^−^.

#### General Procedure for the Preparation of Compounds **99a**,**b** and **99d**–**f**

To a solution of **80** (1 equiv) in 1,4-dioxane, Pd­(PPh_3_)_2_(OAc)_2_ (0.1 equiv) was added, and the mixture was vigorously stirred for 10 min under a nitrogen atmosphere. Then a solution of K_3_PO_4_ (2 equiv) in water (1.5 mL) was added and the reaction mixture was heated at 90 °C. The appropriate boronic acid **98a,b**, **98d–f** (1 equiv), dissolved in a mixture of 1,4-dioxane/H_2_O 9:1 (3.5 mL), was added dropwise and the reaction was stirred for 3–5 h at 90 °C. The reaction was monitored by TLC and 0.7 eq of the appropriate boronic acid were added to the mixture. After 2 h, the reaction was cooled to RT and filtered. The filtrate was extracted with EtOAc (3 × 20 mL), the organic extracts were washed with brine, dried over Na_2_SO_4_ and evaporated to dryness. The crude product was purified by flash column chromatography to afford the corresponding product **99a**–**b** and **99d**–**f**. The products were characterized by MS (ESI) and used directly in the next step.

##### Methyl 2-(8-(Cyclohex-1-en-1-yl)-1-ethyl-6-fluoro-1,3,4,9-tetrahydropyrano­[3,4-*b*]­indol-1-yl)­acetate (**99a**)

The reaction was run with **80** (0.150 g, 0.36 mmol), cyclohex-1-en-1-ylboronic acid (**98a**, 0.045 g, 0.36 mmol), K_3_PO_4_ (0.153 g, 0.72 mmol), Pd­(PPh_3_)_2_(OAc)_2_ (0.027 g, 0.036 mmol), 1,4-dioxane/H_2_O 9:1 (22 mL). After purification by flash column chromatography (silica gel, DCM/EtOAc 9:1) the product **99a** was obtained as a light-yellow solid (103 mg, 77% yield). MS (ESI^–^) *m*/*z*: 370 [M – H]^−^.

##### Methyl 2-(1-Ethyl-6-fluoro-8-(pyridin-3-yl)-1,3,4,9-tetrahydropyrano­[3,4-*b*]­indol-1-yl)­acetate (**99b**)

The reaction was run with **80** (0.100 g, 0.24 mmol), pyridin-3-ylboronic acid (**98b**, 0.030 g, 0.24 mmol), K_3_PO_4_ (0.102 g, 0.48 mmol), Pd­(PPh_3_)_2_(OAc)_2_ (0.018 g, 0.024 mmol), 1,4-dioxane/H_2_O 9:1 (17 mL). After purification by flash column chromatography (silica gel, DCM/EtOAc 9:1) the product **99b** was obtained as a pale-yellow solid (81 mg, 92% yield). MS (ESI^–^) *m*/*z*: 367 [M – H]^−^.

##### Methyl 2-(1-Ethyl-6-fluoro-8-(1*H*-pyrazol-3-yl)-1,3,4,9-tetrahydropyrano­[3,4-*b*]­indol-1-yl)­acetate (**99d**)

The reaction was run with **80** (0.150 g, 0.36 mmol), (1H-pyrazol-3-yl)­boronic acid (**98d**, 0.040 g, 0.36 mmol), K_3_PO_4_ (0.153 g, 0.72 mmol), Pd­(PPh_3_)_2_(OAc)_2_ (0.027 g, 0.036 mmol), 1,4-dioxane/H_2_O 9:1 (22 mL). After purification by flash column chromatography (silica gel, DCM/EtOAc 9:1) the product **99d** was obtained as a white solid (112 mg, 87% yield). MS (ESI^–^) *m*/*z*: 356 [M – H]^−^.

##### Methyl 2-(1-Ethyl-6-fluoro-8-(1-methyl-1*H*-pyrazol-5-yl)-1,3,4,9-tetrahydropyrano­[3,4-*b*]­indol-1-yl)­acetate (**99e**)

The reaction was run with **80** (0.150 g, 0.36 mmol), (1-methyl-1*H*-pyrazol-5-yl)­boronic acid (**98e**, 0.077 g, 0.61 mmol), K_3_PO_4_ (0.153 g, 0.72 mmol), Pd­(PPh_3_)_2_(OAc)_2_ (0.027 g, 0.036 mmol), 1,4-dioxane/H_2_O 9:1 (22 mL). After purification by flash column chromatography (silica gel, DCM/EtOAc 9:1) the product **99e** was obtained as a white solid (49 mg, 37% yield). MS (ESI^–^) *m*/*z*: 370 [M – H]^−^.

##### Methyl 2-(1-Ethyl-6-fluoro-8-(isoxazol-4-yl)-1,3,4,9-tetrahydropyrano­[3,4-*b*]­indol-1-yl)­acetate (**99f**)

The reaction was run with **80** (0.100 g, 0.24 mmol), 4-(4,4,5,5-tetramethyl-1,3,2-dioxaborolan-2-yl)­isoxazole (**98f**, 0.047 g, 0.24 mmol), K_3_PO_4_ (0.102 g, 0.48 mmol), Pd­(PPh_3_)_2_(OAc)_2_ (0.018 g, 0.024 mmol), 1,4-dioxane/H_2_O 9:1 (17 mL). After purification by flash column chromatography (silica gel, PE/EtOAc 9:1) the product **99f** was obtained as a light-yellow oil (71 mg, 83% yield). MS (ESI^–^) *m*/*z*: 357 [M – H]^−^.

##### Methyl 2-(1-Ethyl-6-fluoro-8-(pyrimidin-5-yl)-1,3,4,9-tetrahydropyrano­[3,4-*b*]­indol-1-yl)­acetate (**99c**)

To a stirred solution of **80** (0.150 g, 0.36 mmol, 1 equiv) in 1,4-dioxane (8.1 mL), Pd­(dba)_2_ (0.021 g, 0.036 mmol, 0.1 equiv), an aqueous solution of K_2_CO_3_ (0.88 M, 0.90 mL, 2 equiv) and pyrimidin-5-ylboronic acid (**98c**, 0.045 g, 0.36 mmol, 1 equiv) were added, and the mixture was heated at 90 °C and stirred under a N_2_ atmosphere for 2 h. The mixture was cooled to RT and filtered, and the filtrate was extracted with EtOAc (3 × 20 mL). The organic extracts were washed with brine, dried over Na_2_SO_4_ and evaporated to dryness. The crude product was purified by flash column chromatography (silica gel, DCM/EtOAc 7.5:2.5) to afford **99c** as a yellow solid (41 mg, 31% yield). MS (ESI^–^) *m*/*z*: 368 [M – H]^−^.

#### General Procedure for the Preparation of Compounds **42**–**47**

To a solution of the appropriate ester derivative **99a**–**f** (1 equiv) in 1,4-dioxane, an aqueous solution of LiOH 3 M (5 equiv) was added and the mixture was stirred at RT for 16 h. The reaction was quenched by adding a solution of HCl 2 M and the mixture was extracted with EtOAc (3 × 20 mL). The extracts were washed with brine (30 mL), dried over Na_2_SO_4_ and evaporated to dryness. The crude product was purified by flash column chromatography to afford the corresponding product **42–47**.

##### 2-(8-(Cyclohex-1-en-1-yl)-1-ethyl-6-fluoro-1,3,4,9-tetrahydropyrano­[3,4-*b*]­indol-1-yl)­acetic Acid (**42**)

The reaction was run with **99a** (0.101 g, 0.27 mmol), LiOH 3 M (0.453 mL, 1.36 mmol), 1,4-dioxane (1.6 mL). After purification by flash column chromatography (silica gel, DCM/MeOH 95:5) the product **42** was obtained as a light-yellow solid (95 mg, 98% yield). ^1^H NMR (600 MHz, DMSO-*d*_6_): δ 12.20 (s, 1H, COOH), 10.15 (s, 1H, NH), 7.03 (dd, *J* = 9.1, 2.2 Hz, 1H, ArH_7_), 6.74 (dd, *J* = 10.7, 2.4 Hz, 1H, ArH_5_), 6.04 (s, 1H, CyH_2′_), 3.97–3.85 (m, 2H, OCH_2_−), 2.92 and 2.83 (two signals, d, *J* = 14.1 Hz, 2H, *CH*_2_COOH), 2.61 (m, 2H, CH_2_), 2.47–2.31 (m, 2H, CyH_3′‑6′_), 2.25–2.22 (m, 2H, CyH_6′‑3′_), 2.08–1.95 (m, 2H, C*H*_2_CH_3_), 1.84–1.74 (m, 2H, CyH_4′‑5′_), 1.72–1.64 (m, 2H, CyH_5′‑4′_), 0.64 (t, *J* = 7.3 Hz, 3H, CH_3_). ^13^C NMR (151 MHz, DMSO-*d*_6_): δ 172.05, 156.86 (d, *J*_C–F_ = 231.4 Hz), 138.85, 134.14, 129.23, 128.56 (d, *J*_C–F_ = 8.7 Hz), 127.19, 126.91 (d, *J*_C–F_ = 11.6 Hz), 107.61 (d, *J*_C–F_ = 5.1 Hz), 106.68 (d, *J*_C–F_ = 25.8 Hz), 100.95 (d, *J*_C–F_ = 22.8 Hz), 75.12, 59.72, 42.44, 30.32, 27.95, 25.20, 22.62, 21.79, 21.69, 7.80. MS (ESI^–^) *m*/*z*: 356 [M – H]^−^.

##### 2-(1-Ethyl-6-fluoro-8-(pyridin-3-yl)-1,3,4,9-tetrahydropyrano­[3,4-*b*]­indol-1-yl)­acetic Acid (**43**)

The reaction was run with **99b** (0.081 g, 0.22 mmol), LiOH 3 M (0.366 mL, 1.10 mmol), 1,4-dioxane (1.3 mL). After purification by flash column chromatography (silica gel, DCM/MeOH 9:1) the product **43** was obtained as a light-yellow solid (62 mg, 80% yield). ^1^H NMR (600 MHz, DMSO-*d*_6_): δ 11.09 (s, 1H, NH), 8.84 (s, 1H, Het-H_2_), 8.63 (d, *J* = 4.5 Hz, 1H, Het-H_6_), 8.02 (dt, *J* = 7.8, 1.8 Hz, 1H, Het-H_4_), 7.55 (dd, *J* = 7.7, 5.0 Hz, 1H, Het-H_5_), 7.26 (dd, *J* = 9.3, 2.4 Hz, 1H, ArH_7_), 7.00 (dd, *J* = 9.7, 2.4 Hz, 1H, ArH_5_), 3.95–3.81 (m, 2H, OCH_2_−), 2.85 and 2.74 (two signals, d, *J* = 14.1 Hz, 2H, *CH*_2_COOH), 2.70–2.61 (m, 2H, CH_2_), 2.07–1.95 (m, 2H, C*H*_2_CH_3_), 0.66 (t, *J* = 7.3 Hz, 3H, CH_3_). ^13^C NMR (151 MHz, DMSO-*d*_6_): δ 171.40, 157.03 (d, *J*_C–F_ = 232.4 Hz), 148.98, 148.69, 140.65, 135.94, 133.52, 129.76, 127.62 (d, *J*_C–F_ = 10.1 Hz), 124.07, 122.44 (d, *J*_C–F_ = 10.1 Hz), 109.04 (d, *J*_C–F_ = 26.1 Hz), 107.70, 102.77 (d, *J*_C–F_ = 22.9 Hz), 75.19, 59.67, 43.30, 30.26, 21.87, 7.84. MS (ESI^–^) *m*/*z*: 353 [M – H]^−^.

##### 2-(1-Ethyl-6-fluoro-8-(pyrimidin-5-yl)-1,3,4,9-tetrahydropyrano­[3,4-*b*]­indol-1-yl)­acetic Acid (**44**)

The reaction was run with **99c** (0.086 g, 0.23 mmol), LiOH 3 M (0.388 mL, 1.16 mmol), 1,4-dioxane (1.4 mL). After purification by flash column chromatography (silica gel, DCM/MeOH 95:5) the product **44** was obtained as a light-yellow solid (39 mg, 48% yield). ^1^H NMR (600 MHz, DMSO-*d*_6_): δ 12.10 (s, 1H, COOH), 10.98 (s, 1H, NH), 9.22 (s, 1H, Het-H_2_), 8.99 (s, 2H, Het-H_4–6_), 7.26 (d, *J* = 9.0 Hz, 1H, ArH_7_), 7.05 (d, *J* = 9.0 Hz, 1H, ArH_5_), 4.01–3.76 (m, 2H, OCH_2_−), 2.88 and 2.71 (two signals, d, *J* = 13.8 Hz, 2H, *CH*_2_COOH), 2.68–2.62 (m, 2H, CH_2_), 2.07–1.96 (m, 2H, C*H*_2_CH_3_), 0.59 (t, *J* = 6.4 Hz, 3H, CH_3_). ^13^C NMR (151 MHz, DMSO-*d*_6_): δ 171.64, 157.46, 156.98 (d, *J*_C–F_ = 232.2 Hz), 156.30, 140.41, 131.51, 129.96, 127.76 (d, *J*_C–F_ = 10.1 Hz), 118.91 (d, *J*_C–F_ = 10.1 Hz), 109.40 (d, *J*_C–F_ = 26.7 Hz), 108.06 (d, *J*_C–F_ = 4.3 Hz), 103.53 (d, *J*_C–F_ = 23.1 Hz), 75.29, 59.73, 42.82, 30.43, 21.78, 7.82. MS (ESI^–^) *m*/*z*: 354 [M – H]^−^.

##### 2-(1-Ethyl-6-fluoro-8-(1H-pyrazol-3-yl)-1,3,4,9-tetrahydropyrano­[3,4-*b*]­indol-1-yl)­acetic Acid (**45**)

The reaction was run with **99d** (0.112 g, 0.31 mmol), LiOH 3 M (0.522 mL, 1.57 mmol), 1,4-dioxane (1.8 mL). After purification by flash column chromatography (silica gel, PE/EtOAc/MeOH 7:2:1) the product **45** was obtained as a white solid (93 mg, 87% yield). ^1^H NMR (600 MHz, DMSO-*d*_6_): δ 13.12 (s, 1H, COOH), 10.70 (s, 1H, NH), 7.87 (d, *J* = 1.9 Hz, 1H, Het-H_5_), 7.39 (dd, *J* = 10.7, 2.4 Hz, 1H, ArH_7_), 7.17 (dd, *J* = 9.5, 2.2 Hz, 1H, ArH_5_), 6.94 (d, *J* = 2.1 Hz, 1H, Het-H_4_), 4.00–3.87 (m, 2H, OCH_2_−), 2.98 and 2.81 (two signals, d, *J* = 15.8 Hz, 2H, *CH*_2_COOH), 2.68 (m, 2H, CH_2_), 2.16–1.98 (m, 2H, C*H*_2_CH_3_), 0.72 (t, *J* = 7.3 Hz, 3H, CH_3_). ^13^C NMR (151 MHz, DMSO-*d*_6_): δ 173.15, 157.45 (d, *J*_C–F_ = 231.0 Hz), 139.39, 130.22, 128.98, 127.61 (d, *J*_C–F_ = 10.1 Hz), 123.92, 111.32, 108.00, 106.84 (d, *J*_C–F_ = 27.3 Hz), 103.11 (d, *J*_C–F_ = 7.2 Hz), 102.62 (d, *J*_C–F_ = 23.3 Hz), 75.01, 60.21, 43.07, 30.86, 22.37, 8.32. MS (ESI^–^) *m*/*z*: 342 [M – H]^−^.

##### 2-(1-Ethyl-6-fluoro-8-(1-methyl-1*H*-pyrazol-5-yl)-1,3,4,9-tetrahydropyrano­[3,4-*b*]­indol-1-yl)­acetic Acid (**46**)

The reaction was run with **99e** (0.049 g, 0.13 mmol), LiOH 3 M (0.220 mL, 0.66 mmol), 1,4-dioxane (0.8 mL). After purification by flash column chromatography (silica gel, DCM/MeOH 95:5) the product **46** was obtained as a white solid (45 mg, 96% yield). ^1^H NMR (600 MHz, DMSO-*d*_6_): δ 12.04 (s, 1H, COOH), 10.62 (s, 1H, NH), 7.59 (d, *J* = 1.8 Hz, 1H, Het-H_3_), 7.31 (dd, *J* = 9.3, 2.4 Hz, 1H, ArH_7_), 6.93 (dd, *J* = 10.0, 2.4 Hz, 1H, ArH_5_), 6.44 (d, *J* = 1.9 Hz, 1H, Het-H_4_), 4.00–3.88 (m, 2H, OCH_2_−), 3.70 (s, 3H, NCH_3_), 2.95 and 2.72 (two signals, d, *J* = 14.0 Hz, 2H, *CH*_2_COOH), 2.70–2.61 (m, 2H, CH_2_), 2.05–1.90 (m, 2H, C*H*_2_CH_3_), 0.63 (t, *J* = 7.3 Hz, 3H, CH_3_). ^13^C NMR (151 MHz, DMSO-*d*_6_): δ 171.60, 156.30 (d, *J*_C–F_ = 232.5 Hz), 139.98, 138.95, 138.20, 130.43, 127.24 (d, *J*_C–F_ = 11.6 Hz), 114.51 (d, *J*_C–F_ = 10.1 Hz), 109.97 (d, *J*_C–F_ = 26.5 Hz), 107.99 (d, *J*_C–F_ = 4.3 Hz), 106.86, 103.61 (d, *J*_C–F_ = 21.7 Hz), 75.26, 59.77, 42.46, 37.09, 30.51, 21.76, 7.72. MS (ESI^–^) *m*/*z*: 356 [M – H]^−^.

##### 2-(1-Ethyl-6-fluoro-8-(isoxazol-4-yl)-1,3,4,9-tetrahydropyrano­[3,4-*b*]­indol-1-yl)­acetic Acid (**47**)

The reaction was run with **99f** (0.071 g, 0.20 mmol), LiOH 3 M (0.330 mL, 0.99 mmol), 1,4-dioxane (1.1 mL). After purification by flash column chromatography (silica gel, DCM/MeOH 9:1) the product **47** was obtained as a white solid (62 mg, 90% yield). ^1^H NMR (600 MHz, DMSO-*d*_6_): δ 11.25 (s, 1H, COOH), 10.58 (s, 1H, NH),7.80 (s, 1H, Het-H_5_), 7.60 (s, 1H, Het-H_3_), 6.93 (d, 8.5 Hz, 1H, ArH_7_), 6.62 (d, 8.5 Hz, 1H, ArH_5_), 3.83–3.74 (m, 2H, OCH_2_−), 2.92 and 2.84 (two signals, d, *J* = 13.8 Hz, 2H, *CH*_2_COOH), 2.48 (dd, *J* = 16.0, 11.9 Hz, 2H, CH_2_), 1.93–1.82 (m, 2H, C*H*_2_CH_3_), 0.54–0.51 (m, 3H, CH_3_). ^13^C NMR (151 MHz, DMSO-*d*_6_): δ 171.55, 161.79, 156.69 (d, *J*_C–F_ = 231.8 Hz), 139.59, 138.02, 129.88, 127.46 (d, *J*_C–F_ = 10.1 Hz), 126.67 (d, *J*_C–F_ = 10.1 Hz), 117.08, 108.09 (d, *J*_C–F_ = 23.5 Hz), 106.96 (d, *J*_C–F_ = 4.3 Hz), 102.04 (d, *J*_C–F_ = 24.1 Hz), 75.22, 59.80, 42.54, 30.70, 21.76, 7.83. MS (ESI^–^) *m*/*z*: 343 [M – H]^−^.

##### 2-(8-(Cyclopropylsulfonyl)-1-ethyl-6-fluoro-1,3,4,9-tetrahydropyrano­[3,4-*b*]­indol-1-yl)­acetic Acid (**48**)

To a solution of compound **20** (0.100 g, 0.25 mmol, 1 equiv) in DMSO (5 mL), Cu­(OAc)_2_ (0.198 g, 0.99 mmol, 4 equiv), cyclopropanesulfinic acid (**100**, 0.127 g, 0.99 mmol, 4 equiv) and an excess of NaOH 5 M (57 μL) were added, and the mixture was heated at 120 °C and stirred under a nitrogen atmosphere for 2 h. The mixture was then cooled to RT, diluted with HCl 2 M and filtered over a pad of Celite. The filtrate was then extracted with EtOAc (3 × 20 mL), the extracts were washed with brine, dried over Na_2_SO_4_ and evaporated to dryness. The crude product was purified by semipreparative HPLC (LiChrosper C18e (250 × 25 mm, 5 μM), CH_3_CN/H_2_O 60:40 + TFA 0.1%, *R*_*t*_: 11 min), to afford **48** as a white solid (74 mg, 78% yield). ^1^H NMR (600 MHz, DMSO-*d*_6_): δ 11.01 (s, 1H, NH), 7.63 (d, *J* = 9.0 Hz, 1H, ArH_7_), 7.29 (d, *J* = 9.0 Hz, 1H, ArH_5_), 3.92–3.84 (m, 2H, OCH_2_−), 2.95–2.88 (m, 2H, *CH*_2_COOH), 2.66–2.61 (m, 1H, SO_2_C*H*-), 2.12–1.94 (m, 2H, CH_2_), 1.19–1.15 (m, 2H, C*H*_2_CH_3_), 0.99–0.94 (m, 4H, -C*H*_2_C*H*_2_-), 0.64 (t, *J* = 7.2 Hz, 3H, CH_3_). ^13^C NMR (151 MHz, DMSO-*d*_6_): δ 173.11, 155.46 (d, *J*_C–F_ = 235.8 Hz), 142.19, 129.83 (d, *J*_C–F_ = 8.7 Hz), 128.47, 123.12 (d, *J*_C–F_ = 8.7 Hz), 109.97 (d, *J*_C–F_ = 23.2 Hz), 109.15 (d, *J*_C–F_ = 28.6 Hz), 108.86 (d, *J*_C–F_ = 4.3 Hz), 75.33, 59.90, 42.88, 32.75, 30.44, 22.15, 8.10, 5.54, 4.45. MS (ESI^–^) *m*/*z*: 380 [M – H]^−^. HRMS (ESI-TOF) *m*/*z*: [M – H]^−^ calcd for C_18_H_19_FNO_5_S^–^, 380.0973; found, 380.0979.

##### 2-(8-(Cyclohexylsulfonyl)-1-ethyl-6-fluoro-1,3,4,9-tetrahydropyrano­[3,4-*b*]­indol-1-yl)­acetic Acid (**49**)

To a solution of compound **20** (0.100 g, 0.25 mmol, 1 equiv) in DMSO (5 mL), Cu­(OAc)_2_ (0.198 g, 0.99 mmol, 4 equiv), cyclohexanesulfinic acid (**101**, 0.127 g, 0.99 mmol, 4 equiv) and an excess of NaOH 5 M (57 μL) were added and the mixture was heated at 120 °C and stirred under a nitrogen atmosphere for 3 h. The mixture was then cooled to RT, diluted with HCl 2 M and filtered over a pad of Celite. The filtrate was then extracted with EtOAc (3 × 20 mL), the extracts were washed with brine, dried over Na_2_SO_4_ and evaporated to dryness. The crude product was purified by flash column chromatography (silica gel, first column: PE/EtOAc 8:2; second column: PE/EtOAc/MeOH 8:1.5:0.5) to afford **49** as a light-yellow solid (16 mg, 15% yield). ^1^H NMR (600 MHz, CD_3_OD): δ 10.67 (s, 1H, COOH), 8.09 (s, 1H, NH), 7.54 (d, *J* = 8.8 Hz, 1H, ArH_7_), 7.29 (d, *J* = 8.8 Hz, 1H, ArH_5_), 4.07–3.95 (m, 2H, OCH_2_−), 3.12–3.09 (m, 1H, SO_2_C*H*-), 2.98 and 2.89 (two signals, d, *J* = 15.8 Hz, 2H, *CH*_2_COOH), 2.80–2.71 (m, 2H, CH_2_), 2.22–2.04 (m, 2H, C*H*_2_CH_3_), 2.05–1.97 (m, 2H, CyH_2a_ – CyH_6a_), 1.81 (br, 2H, CyH_2b_ – CyH_6b_), 1.64 (br, 1H, CyH_4a_), 1.48–1.33 (m, 2H, CyH_3a_ – CyH_5a_), 1.32–1.20 (m, 2H, CyH_3b_ – CyH_5b_), 1.19–1.07 (m, 1H, CyH_4b_), 0.80 (t, *J* = 7.4 Hz, 3H, CH_3_). MS (ESI^–^) *m*/*z*: 422 [M – H]^−^.

##### 2-(5-Fluoro-7-(phenylsulfonyl)-1*H*-indol-3-yl)­ethan-1-ol (**103**)

To a stirred solution of **67** (0.500 g, 1.64 mmol, 1 equiv) in DMSO (10 mL), Cu­(OAc)_2_·H_2_O (1.30 g, 6.56 mmol, 4 equiv), sodium benzenesulfinate (**102**, 1.07 g, 6.56 mmol, 4 equiv) and an aqueous solution of NaOH 5 M (0.38 mL) were added, and the mixture was heated at 120 °C and stirred under a nitrogen atmosphere for 4 h. The mixture was then cooled to RT and filtered over a pad of Celite. The suspension was diluted with H_2_O and extracted with diethyl ether (3 × 10 mL). The organic extracts were washed with a solution of EDTA 0.25 M (10 mL), brine (10 mL) and then dried over Na_2_SO_4_ and evaporated to dryness. The crude product was purified by flash column chromatography (silica gel, PE/EtOAc/MeOH 8:1.5:0.5) to afford **103** as a yellow solid (293 mg, 56% yield). ^1^H NMR (600 MHz, CDCl_3_): δ 9.63 (s, 1H, NH), 7.98 (dd, *J* = 8.4, 1.1 Hz, 2H, ArH_2′‑6′_), 7.58–7.54 (m, 1H, ArH_4_), 7.50–7.46 (m, 3H, ArH_3′‑4′‑5′_), 7.43 (dd, *J* = 8.4, 2.3 Hz, 1H, ArH_6_), 7.27 (d, *J* = 2.0 Hz, 1H, ArH_2_), 3.87 (t, *J* = 6.3 Hz, 2H, *CH*_2_OH), 2.95 (t, *J* = 6.3 Hz, 2H, C*H*_2_CH_2_OH). ^13^C NMR (151 MHz, CDCl_3_): δ 156.52 (d, *J*_C–F_ = 239.6 Hz), 141.61, 133.75, 130.76 (d, *J*_C–F_ = 8.7 Hz), 129.52, 129.37, 127.08, 126.44, 123.22 (d, *J*_C–F_ = 8.7 Hz), 113.52 (d, *J*_C–F_ = 4.3 Hz), 110.88 (d, *J*_C–F_ = 7.2 Hz), 110.68, 62.60, 28.38. MS (ESI^+^) *m*/*z*: 320 [M + H]^+^.

##### Methyl 2-(1-Ethyl-6-fluoro-8-(phenylsulfonyl)-1,3,4,9-tetrahydropyrano­[3,4-*b*]­indol-1-yl)­acetate (**104**)

In a flame-dried two-necked-round-bottom flask, kept under N_2_ atmosphere, a solution of **103** (0.290 g, 0.91 mmol, 1 equiv) in anhydrous DCM (14 mL) was added via syringe. Methyl propionyl acetate (**74**, 0.137 mL, 1.09 mmol, 1.2 equiv) was added via a syringe and the mixture was cooled to 0 °C under stirring. BF_3_OEt_2_ (0.090 mL, 0.73 mmol, 0.8 equiv) was added dropwise using a syringe, then the reaction was stirred at RT for 5 h. The reaction was quenched with saturated aqueous NaHCO_3_ solution and extracted with DCM (3 × 30 mL). The combined organic layers were washed with brine, dried over Na_2_SO_4_ and evaporated to dryness. The crude product was purified by flash column chromatography (silica gel, PE/EtOAc 9:1) to afford **104** as a light-yellow solid (220 mg, 56% yield). ^1^H NMR (600 MHz, CDCl_3_): δ 10.35 (s, 1H, NH), 8.04–8.03 (m, 2H, ArH_2′‑6′_), 7.56–7.54 (m, 1H, ArH_4′_), 7.49–7.46 (m, 2H, ArH_3′–5′_), 7.44 (dd, *J* = 8.6, 2.4 Hz, 1H, ArH_7_), 7.34 (dd, *J* = 8.6, 2.4 Hz, 1H, ArH_5_), 4.05–3.91 (m, 2H, OCH_2_−), 3.80 (s, 3H, COO*CH*_3_), 3.02 and 2.94 (two signals, d, *J* = 16.5 Hz, 2H, *CH*_2_COO−), 2.78–2.67 (m, 2H, CH_2_), 2.19–2.01 (m, 2H, *CH*_2_CH_3_), 0.82 (t, *J* = 7.4 Hz, 3H, CH_2_ *CH*_3_). ^13^C NMR (151 MHz, CDCl_3_): δ 172.39, 156.40 (d, *J*_C–F_ = 238.4 Hz), 141.61, 140.47, 133.39, 129.30 (d, *J*_C–F_ = 8.7 Hz), 129.19, 128.57, 127.16, 123.10 (d, *J*_C–F_ = 8.7 Hz), 109.90 (d, *J*_C–F_ = 8.7 Hz), 109.70 (d, *J*_C–F_ = 4.3 Hz), 108.70 (d, *J*_C–F_ = 4.3 Hz), 74.55, 60.18, 52.25, 42.43, 30.46, 21.97, 7.43. MS (ESI^–^) *m*/*z*: 430 [M – H]^−^.

##### 2-(1-Ethyl-6-fluoro-8-(phenylsulfonyl)-1,3,4,9-tetrahydropyrano­[3,4-*b*]­indol-1-yl)­acetic Acid (**50**)

To a stirred solution of **104** (0.220 g, 0.51 mmol, 1 equiv) in 1,4-dioxane (5 mL), an aqueous solution of LiOH 3 M (0.85 mL, 5 equiv) was added and the mixture was stirred at RT for 16 h. The reaction was quenched by adding a solution of HCl 2 M and the mixture was extracted with EtOAc (3 × 20 mL). The extracts were washed with brine (30 mL), dried over Na_2_SO_4_ and evaporated to dryness. The crude product was purified by flash column chromatography (silica gel, DCM/MeOH 95:5) to afford **50** as a white solid (179 mg, 84% yield). ^1^H NMR (600 MHz, DMSO-*d*_6_): δ 12.52 (s, 1H, COOH), 10.87 (s, 1H, NH), 8.14–8.12 (m, 2H, ArH_2′‑6′_), 7.67–7.63 (m, 2H, ArH_7–4′_), 7.59–7.57 (m, 2H, ArH_3′–5′_), 7.53 (dd, *J* = 9.0, 2.4 Hz, 1H, ArH_5_), 3.92–3.85 (m, 2H, CH_2_O−), 3.01 and 2.97 (two signals, d, *J* = 15.2 Hz, 2H, *CH*_2_COOH), 2.66–2.59 (m, 2H, CH_2_), 2.19–1.97 (m, 2H, C*H*_2_CH_3_), 0.63 (t, *J* = 7.4 Hz, 3H, CH_3_). ^13^C NMR (151 MHz, DMSO-*d*_6_): δ 172.89, 155.51 (d, *J*_C–F_ = 235.5 Hz), 141.75, 140.93, 134.02, 129.67, 129.38 (d, *J*_C–F_ = 8.7 Hz), 127.52, 127.11, 122.95 (d, *J*_C–F_ = 8.7 Hz), 110.06 (d, *J*_C–F_ = 23.1 Hz), 108.74 (d, *J*_C–F_ = 28.9 Hz), 108.62 (d, *J*_C–F_ = 4.3 Hz), 74.70, 59.31, 42.09, 29.86, 21.58, 7.48. MS (ESI^–^) *m*/*z*: 416 [M – H]^−^. HRMS (ESI-TOF) *m*/*z*: [M – H]^−^ calcd for C_21_H_19_FNO_5_S^–^, 416.0973; found, 416.0976.

##### Methyl 2-(8-Benzyl-1-ethyl-6-fluoro-1,3,4,9-tetrahydropyrano­[3,4-*b*]­indol-1-yl)­acetate (**106**)

To a stirred solution of **80** (0.767 g, 1.84 mmol, 1 equiv) in 1,4-dioxane/H_2_O (20 mL/5 mL), a solution of benzylboronic acid (**105**, 0.500 g, 3.68 mmol, 2 equiv) in 1,4-dioxane/H_2_O was added. An aqueous solution of K_2_CO_3_ (0.763 g, 5.52 mmol, 3 eq, in 1 mL of H_2_O) and Pd­(dppf)­Cl_2_ (0.202 g, 0.28 mmol, 0.15 equiv) were added and the mixture was heated at 90 °C and stirred overnight under a nitrogen atmosphere. The mixture was then cooled to RT and filtered, the filtrate was extracted with EtOAc (3 × 20 mL), the organic extracts were washed with brine, dried over Na_2_SO_4_ and evaporated to dryness. The crude product was purified by flash column chromatography (silica gel, PE/EtOAc 95:5) to afford **106** as an off-white oil (308 mg, 44% yield). ^1^H NMR (600 MHz, CDCl_3_): δ 8.93 (s, 1H, NH), 7.30–7.28 (m, 4H, ArH_2′,3′,5′,6′_), 7.23–7.20 (m, 1H, ArH_4′_), 7.02 (dd, *J* = 9.5, 2.4 Hz, 1H, ArH_7_), 6.75 (dd, *J* = 10.0, 2.4 Hz, 1H, ArH_5_), 4.16 (s, 2H, C*H*_2_Ph), 4.04–3.87 (m, 2H, CH_2_O−), 3.65 (s, 3H, COOC*H*_3_), 2.95 and 2.83 (two signals, d, *J* = 16.5 Hz, 2H, *CH*_2_COO−), 2.79–2.65 (m, 2H, CH_2_), 2.10–1.88 (m, 2H, *CH*_2_CH_3_), 0.76 (t, *J* = 7.4 Hz, 3H, CH_2_ *CH*_3_). ^13^C NMR (151 MHz, CDCl_3_): δ 172.97, 157.90 (d, *J*_C–F_ = 234.6 Hz), 139.09, 137.96, 131.16, 128.96, 128.72, 126.67 (d, *J*_C–F_ = 10.1 Hz), 126.51, 124.94 (d, *J*_C–F_ = 8.7 Hz), 110.60 (d, *J*_C–F_ = 26.2 Hz), 108.54 (d, *J*_C–F_ = 5.8 Hz), 101.51 (d, *J*_C–F_ = 23.3 Hz), 74.57, 60.51, 52.04, 42.47, 37.95, 30.56, 22.35, 7.57. MS (ESI^–^) *m*/*z*: 380 [M – H]^−^.

##### 2-(8-Benzyl-1-ethyl-6-fluoro-1,3,4,9-tetrahydropyrano­[3,4-*b*]­indol-1-yl)­acetic Acid (**51**)

To a stirred solution of **106** (0.653 g, 1.71 mmol, 1 equiv) in 1,4-dioxane (11 mL), an aqueous solution of LiOH 3 M (2.9 mL, 5 equiv) was added and the mixture was stirred at RT for 16 h. The reaction was quenched by adding a solution of HCl 2 M and the mixture was extracted with EtOAc (3 × 20 mL). The extracts were washed with brine (30 mL), dried over Na_2_SO_4_ and evaporated to dryness. The crude product was purified by flash column chromatography (silica gel, DCM/MeOH 9:1) to afford **51** as a yellow oil (471 mg, 75% yield). ^1^H NMR (600 MHz, DMSO-*d*_6_): δ 12.05 (s, 1H, COOH), 10.75 (s, 1H, NH), 7.30–7.25 (m, 4H, ArH_2′‑3′‑5′‑6′_), 7.19–7.16 (m, 1H, ArH_4′_), 7.00 (dd, *J* = 9.6, 2.4 Hz, 1H, ArH_7_), 6.58 (dd, *J* = 10.3, 2.4 Hz, 1H, ArH_5_), 4.17 (s, 2H, *CH*_2_Ph), 3.96–3.87 (m, 2H, CH_2_O−), 2.92 and 2.72 (two signals, d, *J* = 13.5 Hz, 2H, *CH*_2_COOH), 2.66–2.55 (m, 2H, CH_2_), 2.05–1.99 (m, 2H, C*H*_2_CH_3_), 0.62 (t, *J* = 7.4 Hz, 3H, CH_3_). ^13^C NMR (151 MHz, CDCl_3_): δ 175.40, 158.07 (d, *J*_C–F_ = 235.1 Hz), 139.09, 136.72, 131.42, 128.92, 128.87, 126.79 (d, *J*_C–F_ = 10.1 Hz), 126.72, 124.93 (d, *J*_C–F_ = 8.8 Hz), 111.15 (d, *J*_C–F_ = 26.2 Hz), 108.76 (d, *J*_C–F_ = 4.6 Hz), 101.76 (d, *J*_C–F_ = 23.4 Hz), 75.08, 60.83, 42.49, 38.00, 30.88, 22.20, 7.69. MS (ESI^–^) *m*/*z*: 366 [M – H]^−^. HRMS (ESI-TOF) *m*/*z*: [M – H]^−^ calcd for C_22_H_21_FNO_3_^–^, 366.1511; found, 366.1515.

##### Methyl 2-(6-Bromo-5-fluoro-1*H*-indol-3-yl)-2-oxoacetate (**108**)

To a solution of 6-bromo-5-fluoro-1*H*-indole (**107**, 1 g, 4.7 mmol, 1 equiv) in dry Et_2_O (10 mL) at 0 °C, oxalyl chloride (0.47 mL, 5.61 mmol, 1.2 equiv) was added dropwise. The mixture was warmed to RT and stirred for 1 h. Methanol (1 mL, 23.5 mmol, 5 equiv) was then added and the mixture was stirred for 24 h at RT. H_2_O (10 mL) was added to the mixture to obtain a yellow solid which was then filtered and washed with Et_2_O. The solid was dried to afford **108** (1.08 g, 76% yield) which was used in the following step without further purification. ^1^H NMR (600 MHz, DMSO-*d*_6_): δ 12.50 (s, 1H, NH), 8.49 (d, *J* = 3.3 Hz, 1H, ArH_2_), 7.88 (d, *J* = 9.3 Hz, 1H, ArH_4_), 7.79 (d, *J* = 5.9 Hz, 1H, ArH_7_), 3.82 (s, 3H, CH_3_). ^13^C NMR (151 MHz, DMSO-*d*_6_): δ 178.28, 163.26, 154.82 (d, *J*_C–F_ = 238.1 Hz) 140.35, 133.68, 125.63 (d, *J*_C–F_ = 10.1 Hz), 117.07, 112.50 (d, *J*_C–F_ = 4.3 Hz), 107.29 (d, *J*_C–F_ = 25.9 Hz), 103.99 (d, *J*_C–F_ = 24.6 Hz), 52.72. MS (ESI^–^) *m*/*z*: 298/300 [M – H]^−^.

##### 2-(6-Bromo-5-fluoro-1*H*-indol-3-yl)­ethan-1-ol (**109**)

Compound **108** (1.08 g, 3.6 mmol, 1 equiv) was suspended in dry THF (10 mL) and BH_3_·DMS (2.0 M in THF, 7.17 mL, 14 mmol, 4 equiv) was added. The mixture was heated to reflux for 4 h and stirred under a nitrogen atmosphere. The solution was cooled to RT, H_2_O (10 mL) and a saturated aqueous Na_2_CO_3_ solution (10 mL) were added, and the solution was extracted with EtOAc (3 × 20 mL). The combined organic layers were washed with brine (30 mL), dried over Na_2_SO_4_ and evaporated to dryness. The crude product was purified by flash column chromatography (silica gel, DCM/MeOH 99:1) to afford **109** as a yellow solid (417 mg, 45% yield). ^1^H NMR (600 MHz, CDCl_3_): δ 8.13 (s, 1H, NH), 7.51 (d, *J* = 5.6 Hz, 1H, ArH_7_), 7.31 (d, *J* = 9.0 Hz, 1H, ArH_4_), 7.11 (d, *J* = 2.3 Hz, 1H, ArH_2_), 3.89 (t, *J* = 6.2 Hz, 2H, C*H*_2_OH), 2.95 (t, *J* = 6.4 Hz, 2H, C*H*_2_CH_2_OH), 2.10 (s, 1H, OH). ^13^C NMR (151 MHz, CDCl_3_): δ 153.73 (d, *J*_C–F_ = 236.5 Hz), 133.24, 127.35 (d, *J*_C–F_ = 8.7 Hz), 124.67, 115.32, 113.05 (d, *J*_C–F_ = 4.3 Hz), 104.92 (d, *J*_C–F_ = 24.6 Hz), 103.63 (d, *J*_C–F_ = 25.0 Hz), 62.93, 28.64. MS (ESI^–^) *m*/*z*: 256/258 [M – H]^−^.

##### Methyl 2-(7-Bromo-1-ethyl-6-fluoro-1,3,4,9-tetrahydropyrano­[3,4-*b*]­indol-1-yl)­acetate (**110**)

In a flame-dried two-necked-round-bottom flask, kept under N_2_ atmosphere, a solution of **109** (0.417 g, 1.62 mmol, 1 equiv) in anhydrous DCM was added via syringe. Methyl propionyl acetate (**74**, 0.24 mL, 1.94 mmol, 1.2 equiv) was added via a syringe and the mixture was cooled to 0 °C under stirring. BF_3_OEt_2_ (0.16 mL, 1.3 mmol, 0.8 equiv) was added dropwise using a syringe, then the reaction was stirred at RT until completion (5 h). The reaction was quenched with saturated aqueous NaHCO_3_ solution (20 mL) and extracted with DCM (3 × 30 mL). The combined organic layers were washed with brine (30 mL), dried over Na_2_SO_4_ and evaporated to dryness. The crude product was purified by flash column chromatography (silica gel, DCM) to afford **110** as a white solid (400 mg, 67% yield). ^1^H NMR (600 MHz, CDCl_3_): δ 9.16 (s, 1H, NH), 7.51 (d, *J* = 5.7 Hz, 1H, ArH_8_), 7.20 (d, *J* = 9.1 Hz, 1H, ArH_5_), 4.06–3.88 (m, 2H, CH_2_O−), 3.73 (s, 3H, COO*CH*_3_), 3.01 and 2.91 (two signals, d, *J* = 16.9 Hz, 2H, *CH*_2_COO−), 2.81–2.64 (m, 2H, CH_2_), 2.16–1.92 (m, 2H, C*H*_2_CH_3_), 0.81 (t, *J* = 7.4 Hz, 3H, CH_3_). ^13^C NMR (151 MHz, CDCl_3_): δ 173.36, 153.69 (d, *J*_C–F_ = 235.9 Hz), 138.90, 132.61, 126.24 (d, *J*_C–F_ = 8.7 Hz), 115.23, 108.51 (d, *J*_C–F_ = 4.3 Hz), 104.30 (d, *J*_C–F_ = 24.5 Hz), 102.72 (d, *J*_C–F_ = 24.5 Hz), 74.58, 60.50, 52.24, 42.56, 30.56, 22.24, 7.60. MS (ESI^–^) *m*/*z*: 368/370 [M – H]^−^.

#### General Procedure for the Preparation of Compounds **111a–c**

To a solution of **110** (1 equiv) in 1,4-dioxane, Pd­(PPh_3_)_2_(OAc)_2_ (0.1 equiv) was added and the mixture was vigorously stirred for 10 min under a nitrogen atmosphere. Then a solution of K_3_PO_4_ (2 equiv) in water (1.5 mL) was added and the reaction mixture was heated at 90 °C. The appropriate boronic acid **96a**–**c** (1 equiv), dissolved in a mixture of 1,4-dioxane/H_2_O 9:1 (3.5 mL), was added dropwise and the reaction was stirred for 3–5 h at 90 °C. The reaction was monitored by TLC and 0.7 equiv of the appropriate boronic acid were added to the mixture. After 2 h, the reaction was cooled to RT and filtered. The filtrate was extracted with EtOAc (3 × 20 mL), the organic extracts were washed with brine, dried over Na_2_SO_4_ and evaporated to dryness. The crude product was purified by flash column chromatography (silica gel, PE/EtOAc 9:1) and then repurified using semipreparative HPLC (column: LiChrospher C18e Merck, 250 × 25 mm, 5 μm; eluent: CH_3_CN/H_2_O 70:30 + 0.1% TFA; flow 20 mL/min, UV detection λ = 226 nm, with the indicated *t*_R_) to afford the corresponding product **111a**–**c**. The products were characterized by MS (ESI) and used directly in the next step.

##### Methyl 2-(1-Ethyl-6-fluoro-7-phenyl-1,3,4,9-tetrahydropyrano­[3,4-*b*]­indol-1-yl)­acetate (**111a**)

The reaction was run with **110** (0.100 g, 0.27 mmol), phenylboronic acid (**96a**, 0.033 g, 0.27 mmol), K_3_PO_4_ (0.115 g, 0.54 mmol), Pd­(PPh_3_)_2_(OAc)_2_ (0.020 g, 0.027 mmol), 1,4-dioxane/H_2_O (20 mL). Semipreparative HPLC: *t*_R_ = 18 min. Product **111a** was obtained as a pale-yellow oil (42 mg, 44% yield). MS (ESI^–^) *m*/*z*: 366 [M – H]^−^.

##### Methyl 2-(7-(2-Chlorophenyl)-1-ethyl-6-fluoro-1,3,4,9-tetrahydropyrano­[3,4-*b*]­indol-1-yl)­acetate (**111b**)

The reaction was run with **110** (0.100 g, 0.27 mmol), 2-chlorophenylboronic acid (**96b**, 0.042 g, 0.27 mmol), K_3_PO_4_ (0.115 g, 0.54 mmol), Pd­(PPh_3_)_2_(OAc)_2_ (0.020 g, 0.027 mmol), 1,4-dioxane/H_2_O (20 mL). Semipreparative HPLC: *t*_R_ = 17 min. Product **111b** was obtained as a pale-yellow oil (80 mg, 74% yield). MS (ESI^–^) *m*/*z*: 400/402 [M – H]^−^.

##### Methyl 2-(7-(3-Chlorophenyl)-1-ethyl-6-fluoro-1,3,4,9-tetrahydropyrano­[3,4-*b*]­indol-1-yl)­acetate (**111c**)

The reaction was run with **110** (0.100 g, 0.27 mmol), 3-chlorophenylboronic acid (**96c**, 0.042 g, 0.27 mmol), K_3_PO_4_ (0.115 g, 0.54 mmol), Pd­(PPh_3_)_2_(OAc)_2_ (0.020 g, 0.027 mmol), 1,4-dioxane/H_2_O (20 mL). Semipreparative HPLC: *t*_R_ = 25 min. Product **111c** was obtained as a pale-yellow oil (70 mg, 65% yield). MS (ESI^–^) *m*/*z*: 400/402 [M – H]^−^.

#### General Procedure for the Preparation of Compounds **22**, **24** and **26**

To a solution of the appropriate ester derivative **111a**–**c** (1 equiv) in 1,4-dioxane, an aqueous solution of LiOH 3 M (5 equiv) was added, and the mixture was stirred at RT for 16 h. The reaction was quenched by adding a solution of HCl 2 M and the mixture was extracted with EtOAc (3 × 20 mL). The extracts were washed with brine (30 mL), dried over Na_2_SO_4_ and evaporated to dryness. The crude product was purified by flash column chromatography to afford the corresponding product **22**, **24** and **26**.

##### 2-(1-Ethyl-6-fluoro-7-phenyl-1,3,4,9-tetrahydropyrano­[3,4-*b*]­indol-1-yl)­acetic Acid (**22**)

The reaction was run with **111a** (0.084 g, 0.23 mmol), LiOH 3 M (0.367 mL, 1.15 mmol), 1,4-dioxane (1.4 mL). After purification by flash column chromatography (silica gel, DCM/MeOH 98:2) the product **22** was obtained as a white solid (51 mg, 63% yield). ^1^H NMR (600 MHz, DMSO-*d*_6_): δ 12.02 (s, 1H, COOH), 10.79 (s, 1H, NH), 7.38 (d, *J* = 7.3 Hz, 2H, ArH_2′‑6′_), 7.25 (t, *J* = 7.4 Hz, 2H, ArH_3′–5′_), 7.18–7.15 (m, 2H, ArH_8–4′_), 7.13 (d, *J* = 11.5 Hz, 1H, ArH_5_), 3.84–3.74 (m, 2H, CH_2_O−), 2.78 and 2.52 (two signals, d, *J* = 14.0 Hz, 2H, *CH*_2_COOH), 2.50–2.36 (m, 2H, CH_2_), 1.95–1.75 (m, 2H, C*H*_2_CH_3_), 0.51 (t, *J* = 7.2 Hz, 3H, CH_3_). ^13^C NMR (151 MHz, DMSO-*d*_6_): δ 171.50, 154.21 (d, *J*_C–F_ = 235.1 Hz), 139.61, 137.29, 132.96, 129.23, 128.73, 127.20, 126.12 (d, *J*_C–F_ = 8.7 Hz), 122.36 (d, *J*_C–F_ = 17.7 Hz), 112.34, 107.37, 103.76 (d, *J*_C–F_ = 23.3 Hz), 75.46, 60.20, 43.19, 31.22, 22.03, 8.08. MS (ESI^–^) *m*/*z*: 352 [M – H]^−^.

##### 2-(7-(2-Chlorophenyl)-1-ethyl-6-fluoro-1,3,4,9-tetrahydropyrano­[3,4-*b*]­indol-1-yl)­acetic Acid (**24**)

The reaction was run with **111b** (0.080 g, 0.20 mmol), LiOH 3 M (0.332 mL, 1.0 mmol), 1,4-dioxane (1.3 mL). After purification by flash column chromatography (silica gel, DCM/MeOH 98:2) the product **24** was obtained as a white solid (52 mg, 68% yield). ^1^H NMR (600 MHz, CDCl_3_ + CD_3_OD): δ 10.44 (s, 1H, NH), 7.89–7.52 (m, 5H, ArH_8–3′‑4′‑5′‑6′_), 7.49 (d, *J* = 10.3 Hz, 1H, ArH_5_), 4.38–4.30 (m, 2H, CH_2_O−), 3.28 and 3.19 (two signals, d, *J* = 15.1 Hz, 2H, *CH*_2_COOH), 3.16–2.91 (m, 2H, CH_2_), 2.41 (q, *J* = 7.0 Hz, 2H, C*H*_2_CH_3_), 1.13 (t, *J* = 7.1 Hz, 3H, CH_3_). ^13^C NMR (151 MHz, CDCl_3_ + CD_3_OD): δ 173.89, 154.28 (d, *J*_C–F_ = 235.4 Hz), 138.52, 136.28, 133.78, 131.90, 131.81, 129.00, 128.39, 126.42 (d, *J*_C–F_ = 10.1 Hz), 126.14, 120.96 (d, *J*_C–F_ = 20.2 Hz), 112.75 (d, *J*_C–F_ = 4.3 Hz), 107.39 (d, *J*_C–F_ = 2.9 Hz), 102.66 (d, *J*_C–F_ = 24.3 Hz), 75.37, 60.39, 42.90, 30.53, 21.83, 7.15. MS (ESI^–^) *m*/*z*: 386/388 [M – H]^−^.

##### 2-(7-(3-Chlorophenyl)-1-ethyl-6-fluoro-1,3,4,9-tetrahydropyrano­[3,4-*b*]­indol-1-yl)­acetic Acid (**26**)

The reaction was run with **111c** (0.070 g, 0.18 mmol), LiOH 3 M (0.291 mL, 0.87 mmol), 1,4-dioxane (1.1 mL). After purification by flash column chromatography (silica gel, DCM/MeOH 99:1) and a second purification by semipreparative HPLC (column: LiChrospher C18e Merck, 250 × 25 mm, 5 μm; eluent: CH_3_CN/H_2_O 60:40 + 0.1% TFA; flow 20 mL/min, UV detection λ = 240 nm, *t*_R_ = 24 min) the product **26** was obtained as a white solid (8 mg, 12% yield). ^1^H NMR (600 MHz, CDCl_3_): δ 9.44 (s, 1H, NH), 7.54 (d, *J* = 1.4 Hz, 1H, ArH_2′_), 7.44 (d, *J* = 7.6 Hz, 1H, ArH_8_), 7.34 (t, *J* = 7.8 Hz, 1H, ArH_5′_), 7.31–7.28 (m, 2H, ArH_4′‑6′_), 7.20 (d, *J* = 11.2 Hz, 1H, ArH_5_), 4.08–3.96 (m, 2H, CH_2_O−), 2.97 and 2.92 (two signals, d, *J* = 16.5 Hz, 2H, *CH*_2_COOH), 2.83–2.71 (m, 2H, CH_2_), 2.15–1.98 (m, 2H, C*H*_2_CH_3_), 0.83 (t, *J* = 7.3 Hz, 3H, CH_3_). MS (ESI^–^) *m*/*z*: 386/388 [M – H]^−^.

##### 2-(7-Bromo-1-ethyl-6-fluoro-1,3,4,9-tetrahydropyrano­[3,4-*b*]­indol-1-yl)­acetic Acid (**112**)

To a stirred solution of **110** (0.220 g, 0.60 mmol, 1 equiv) in 1,4-dioxane (3.5 mL), an aqueous solution of LiOH 3 M (0.995 mL, 3.0 mmol, 5 equiv) was added and the mixture was stirred at RT for 16 h. The reaction was quenched by adding a solution of HCl 2 M (5 mL) and the mixture was extracted with EtOAc (3 × 20 mL). The extracts were washed with brine (30 mL), dried over Na_2_SO_4_ and evaporated to dryness. The crude product was purified by flash column chromatography (silica gel, DCM/MeOH 95:5) to afford **112** as a white solid (194 mg, 92% yield). ^1^H NMR (600 MHz, DMSO-*d*_6_): δ 12.04 (s, 1H, COOH), 10.95 (s, 1H, NH), 7.53 (d, *J* = 5.8 Hz, 1H, ArH_8_), 7.33 (d, *J* = 9.6 Hz, 1H, ArH_5_), 3.93–3.82 (m, 2H, CH_2_O−), 2.83 and 2.62 (two signals, d, *J* = 13.8 Hz, 2H, *CH*_2_COOH), 2.59–2.44 (m, 2H, CH_2_), 2.00–1.87 (m, 2H, C*H*_2_CH_3_), 0.59 (t, *J* = 7.3 Hz, 3H, CH_3_). ^13^C NMR (151 MHz, DMSO-*d*_6_): δ 171.28, 152.50 (d, *J*_C–F_ = 232.8 Hz), 139.73, 133.00, 126.04 (d, *J*_C–F_ = 8.7 Hz), 114.93, 107.62 (d, *J*_C–F_ = 4.3 Hz), 104.23 (d, *J*_C–F_ = 24.3 Hz), 100.53 (d, *J*_C–F_ = 24.5 Hz), 75.24, 59.88, 42.78, 31.04, 21.69, 7.92. MS (ESI^–^) *m*/*z*: 354/356 [M – H]^−^.

##### 2-(7-(Cyclopropylsulfonyl)-1-ethyl-6-fluoro-1,3,4,9-tetrahydropyrano­[3,4-*b*]­indol-1-yl)­acetic Acid (**52**)

To a solution of **112** (0.100 g, 0.28 mmol, 1 equiv) in 10 mL of NMP, cyclopropylsulfinic acid (**100**, 0.180 g, 1.4 mmol, 5 equiv) and CuI (0.269 g, 1.4 mmol, 5 equiv) were added and the mixture was stirred at 150 °C for 18 h under a nitrogen atmosphere. The mixture was cooled to RT, quenched with HCl 1 N (10 mL) and filtered. The filtrate was extracted with EtOAc (3 × 20 mL), the organic extracts washed with brine (30 mL), dried over Na_2_SO_4_ and evaporated to dryness. The crude product was purified by flash column chromatography (silica gel, DCM/MeOH 99:1) and then by semipreparative HPLC (column: LiChrospher C18e Merck, 250 × 25 mm, 5 μm; eluent: CH_3_CN/H_2_O 50:50 + 0.1% TFA; flow 20 mL/min, UV detection λ = 226 nm, *t*_R_ = 10 min) to afford **52** as a white solid (27 mg, 25% yield). ^1^H NMR (600 MHz, DMSO-*d*_6_): δ 12.11 (s, 1H, COOH), 11.45 (s, 1H, NH), 7.72 (d, *J* = 5.6 Hz, 1H, ArH_8_), 7.48 (d, *J* = 11.3 Hz, 1H, ArH_5_), 3.94–3.90 (m, 2H, CH_2_O−), 2.94–2.82 (m, 2H, *CH*_2_COOH), 2.72–2.67 (m, 3H, C*H*_2_CH_2_O- and –SO_2_C*H*-), 2.08–1.92 (m, 2H, C*H*_2_CH_3_), 1.12–1.03 (m, 4H, -C*H*_2_ *CH*_2_-), 0.66 (t, *J* = 7.2 Hz, 3H, CH_3_). ^13^C NMR (151 MHz, DMSO-*d*_6_): δ 162.39, 152.92 (d, *J*_C–F_ = 239.3 Hz), 143.72, 130.71, 129.90 (d, *J*_C–F_ = 9.8 Hz), 120.25 (d, *J*_C–F_ = 19.5 Hz), 112.19, 108.15 (d, *J*_C–F_ = 4.6 Hz), 104.64 (d, *J*_C–F_ = 22.7 Hz), 75.14, 59.76, 43.23, 32.51, 30.82, 21.62, 7.78, 5.30, 5.22. MS (ESI^–^) *m*/*z*: 380 [M – H]^−^.

##### Methyl 2-(7-Benzyl-1-ethyl-6-fluoro-1,3,4,9-tetrahydropyrano­[3,4-*b*]­indol-1-yl)­acetate (**113**)

To a stirred solution of **110** (0.100 g, 0.27 mmol, 1 equiv) in a mixture of 1,4-dioxane/H_2_O 9:1 (1.2 mL), a solution of benzylboronic acid (**105**, 0.074 g, 0.54 mmol, 2 equiv) in 1,4-dioxane/H_2_O 9:1 was added. An aqueous solution of K_2_CO_3_ (0.110 g, 0.81 mmol, 3 equiv, in 0.5 mL of H_2_O) and Pd­(dppf)­Cl_2_ (0.030 g, 0.04 mmol, 0.15 equiv) were added and the mixture was heated at 90 °C and stirred overnight under a nitrogen atmosphere. The mixture was then cooled to RT and filtered, the filtrate was extracted with EtOAc (3 × 20 mL), the organic extracts were washed with brine, dried over Na_2_SO_4_ and evaporated to dryness. The crude product was purified by flash column chromatography (silica gel, PE/DCM 6:4) and then by semipreparative HPLC (column: LiChrospher C18e Merck, 250 × 25 mm, 5 μm; eluent: CH_3_CN/H_2_O 70:30 + 0.1% TFA; flow 20 mL/min, UV detection λ = 226 nm, *t*_R_ = 18 min) to afford **113** as a pale-yellow oil (21 mg, 20% yield). ^1^H NMR (600 MHz, CDCl_3_): δ 8.94 (s, 1H, NH), 7.30–7.25 (m, 4H, ArH_2′‑3′‑5′‑6′_), 7.22–7.19 (m, 1H, ArH_4′_), 7.13 (d, *J* = 10.3 Hz, 1H, ArH_7_), 7.04 (d, *J* = 6.2 Hz, 1H, ArH_5_), 4.09 (s, 2H, *CH*_2_Ph), 4.05–3.90 (m, 2H, CH_2_O−), 3.71 (s, 3H, COOCH_3_), 2.99 and 2.90 (two signals, d, *J* = 16.5 Hz, 2H, *CH*_2_COO−), 2.80–2.67 (m, 2H, CH_2_), 2.15–1.94 (m, 2H, *CH*_2_CH_3_), 0.81 (t, *J* = 7.4 Hz, 3H, CH_3_). ^13^C NMR (151 MHz, CDCl_3_): δ 173.08, 156.24 (d, *J*_C–F_ = 235.5 Hz), 140.70, 137.59, 132.17, 128.85, 128.40, 126.01, 125.34 (d, *J*_C–F_ = 10.1 Hz), 122.78 (d, *J*_C–F_ = 20.2 Hz), 112.37 (d, *J*_C–F_ = 5.8 Hz), 108.03 (d, *J*_C–F_ = 4.3 Hz), 103.15 (d, *J*_C–F_ = 24.6 Hz), 74.52, 60.46, 51.95, 42.56, 35.48, 30.51, 22.26, 7.50. MS (ESI^–^) *m*/*z*: 380 [M – H]^−^.

##### 2-(7-Benzyl-1-ethyl-6-fluoro-1,3,4,9-tetrahydropyrano­[3,4-*b*]­indol-1-yl)­acetic Acid (**53**)

To a stirred solution of **113** (0.050 g, 0.13 mmol, 1 equiv) in 1,4-dioxane (1 mL), an aqueous solution of LiOH 3 M (0.218 mL, 0.65 mmol, 5 equiv) was added and the mixture was stirred at RT for 16 h. The reaction was quenched by adding a solution of HCl 2 M (2 mL) and the mixture was extracted with EtOAc (3 × 20 mL). The extracts were washed with brine (30 mL), dried over Na_2_SO_4_ and evaporated to dryness. The crude product was purified by flash column chromatography (silica gel, DCM/MeOH 98:2) to afford **53** as a white solid (34 mg, 71% yield). ^1^H NMR (600 MHz, DMSO-*d*_6_): δ 12.00 (s, 1H, COOH), 10.68 (s, 1H, NH), 7.27 (t, *J* = 7.4 Hz, 2H, ArH_3′–5′_), 7.21 (d, *J* = 7.2 Hz, 2H, ArH_2′‑6′_), 7.17 (t, *J* = 7.2 Hz, 1H, ArH_4′_), 7.11–7.06 (m, 2H, ArH_5–8_), 3.97 (s, 2H, *CH*_2_Ph), 3.91–3.80 (m, 2H, CH_2_O−), 2.82 and 2.63 (two signals, d, *J* = 13.5 Hz, 2H, *CH*_2_COOH), 2.56–2.43 (m, 2H, CH_2_), 2.00–1.82 (m, 2H, C*H*_2_CH_3_), 0.58 (t, *J* = 7.3 Hz, 3H, CH_3_). ^13^C NMR (151 MHz, DMSO-*d*_6_): δ 171.24, 155.30 (d, *J*_C–F_ = 232.4 Hz), 140.87, 138.17, 132.44, 128.57, 128.40, 125.96, 125.00 (d, *J*_C–F_ = 10.1 Hz), 121.29 (d, *J*_C–F_ = 20.0 Hz), 112.43 (d, *J*_C–F_ = 5.3 Hz), 106.87 (d, *J*_C–F_ = 4.3 Hz), 102.79 (d, *J*_C–F_ = 24.2 Hz), 75.14, 59.92, 42.97, 34.91, 30.94, 21.83, 7.87. MS (ESI^–^) *m*/*z*: 366 [M – H]^−^.

### Pharmacological Screening

#### Isolation of Human Platelets and Analysis of Platelet Aggregation

Platelets were obtained from human buffy coat (Ospedale Niguarda blood-bank, both genders donors, age 18–60 years), 40 mL buffy coat diluted with Hanks’ Balanced Salt Solution (HBSS 1:1, KCl 0.40 g/L; KH_2_PO_4_ 0.06 g/L; NaCl 8.00 g/L; NaHCO_3_ 0.35 g/L; Na_2_HPO_4_ 0.048 g/L; d-glucose 1.00 g/L) was centrifuged at 280*g* for 15 min at room temperature to obtain platelet-rich plasma (PRP), which was further centrifuged at 650*g* for 10 min at room temperature. The pelleted platelets were suspended in 10–15 mL washing buffer (mM composition: citric acid monohydrate 39, glucose monohydrate 5, KCl 5, CaCl_2_ 2, MgCl_2_·6H_2_O 1, NaCl 103, pH 6.5), again centrifuged at 650*g* for 15 min at room temperature, and finally resuspended in HBSS added with CaCl_2_·2H_2_O (0.185 g/L), MgCl_2_·6H_2_O (0.10 g/L), MgSO_4_·7H_2_O (0.10 g/L). Platelet concentration was adjusted to approximately 2 × 10^8^ cell mL^–1^ and aggregation assessed by the Born turbidimetric assay (Pap-8 aggregometer, Bio/Data Corporation, Sentinel Diagnostics, Milano, Italy), at 37 °C in a 0.5 mL sample. After incubation with drug, or vehicle as control (Dimethyl sulfoxide -DMSO- maximum 0.2%, v/v), for 5 min at 37 °C, platelet aggregation was induced by U-46619 (0.1 μM), under continuous stirring, and monitored for 6–8 min. Experiments were repeated at least in triplicate using platelets from different subjects. Due to the intersubject variability in platelet response to the agonist challenge, the activity of each compound was expressed as percent inhibition versus its corresponding control aggregation.

#### COX-1 Inhibitory Activity (Human Platelets)

Human platelets samples (0.5 mL) were treated with increasing concentrations of the tested compounds or vehicle (DMSO), incubated at 37 °C in a Dubnoff bath for 30 min and challenged with 2 μM calcium ionophore A23187 for 15 min at 37 °C. Reaction was stopped by centrifugation at 1500*g* (4 °C) for 5 min. TXA_2_ metabolite (TXB_2_) production was evaluated in the supernatant by mass spectrometry. The activity of each compound was expressed as percent inhibition versus its corresponding control challenge.

#### COX-2 Inhibitory Activity (Lymphomonocytes)

The study of COX-2 activity was carried out in a lymphomonocytes suspension isolated from buffy coat (diluted in HBSS 1:1). PRP deprived Buffy coat was carefully layered on same volume of Ficoll–Paque for gradient density centrifugation (400*g* for 30 min at 10 °C); enriched cell ring was collected and washed twice with HBSS and centrifuged (280 *g* for 15 min at 10 °C) in order to remove the remaining suspended platelets. Soon after, a lysis buffer (NaCl 0.2% weight/volume, w/v) was added to remove the remaining erythrocytes, immediately balanced with an equal volume of equilibrating solution (NaCl 1.6% + saccharose 0.2% w/v). Lymphomonocytes were finally resuspended in HBSS added with CaCl_2_·2H_2_O (0.185 g/L), MgCl_2_·6H_2_O (0.10 g/L), MgSO_4_·7H_2_O (0.10 g/L). 0.5 mL samples (cell concentration adjusted at approximately 2–4 × 10^6^ cell mL^–1^) were pretreated (30 min, 37 °C) with increasing concentrations of the tested compound, or vehicle as control, and then challenged with LPS (10 μg/mL, 24 h, 37 °C). COX-2 activity was evaluated quantifying PGE_2_ production. The activity of each compound was expressed as percent inhibition of PGE_2_ production versus its corresponding control after 24 h of challenge. PGE_2_ determination was carried out by mass spectrometry.

#### Mass Spectrometry Determination of Eicosanoids

Quantitative determinations of TXB_2_ and PGE_2_ were assessed by LC–MS/MS; for each eicosanoid 2 different transitions were selected: TXB_2_ *m*/*z* 369 > 169 and *m*/*z* 369 > 195; PGE_2_ *m*/*z* 351 > 271 and *m*/*z* 351 > 195, the most abundant was used as quantifier and the second as qualifier. Quantitation was made using the isotopic dilution of the deuterated internal standards *d*_4_-TXB_2_ and *d*_4_-PGE_2_, monitoring the transitions *m*/*z* 373 > 173 and *m*/*z* 355 > 275, respectively. The MRM (multiple reaction monitoring) analyzes were carried out using a triple quadrupole spectrometer LC-(ESI)-MS/MS (ABSciex API 4000) operated in negative ion mode. Chromatographic separation was obtained using a reverse phase column (EVO Kinetex C18, 2.1 × 150 mm × 5 μm, Phenomenex), with a flow of 0.4 mL/min and a gradient from 70% solvent A (H_2_O + 0.05 mL/L acetic acid pH = 5.7) to 100% solvent B (65% ACN + 35% methanol) in 3.5 min.

### Effects of Selected Compounds on the Inhibition of COX-1 and COX-2 Activities in Human Whole Blood

Peripheral venous blood samples were drawn from healthy volunteers (*n* = 6, 23–50 years old) when they had not taken any nonsteroidal anti-inflammatory drug (NSAID) during the 2 weeks preceding the study. This study was carried out following the recommendations of the Declaration of Helsinki after approval by the local Ethics Committee of “G. d’ Annunzio” University of Chieti-Pescara, and informed consent was obtained from each subject.

Compounds (5–20 mM) were dissolved in DMSO, and 2 μL aliquots of the solutions were pipetted directly into test tubes to give final concentrations of 0.01–40 μM in blood. Different concentrations of each compound or the vehicle (DMSO) were incubated with heparinized whole blood samples in the presence of LPS (10 μg/mL) for 24 h as previously described.^62^ Plasma was separated by centrifugation (10 min at 2000 rpm, at 4 °C) and kept at −80 °C until assayed for PGE_2_, as an index of LPS-induced monocyte COX-2 activity by a specific radioimmunoassay.^62^ Parallelly, different concentrations of each compound or the vehicle (DMSO) were incubated with whole blood samples (drawn from the same donors in different occasions) and allowed to clot at 37 °C for 1 h. At the end of incubation, serum was obtained by centrifugation (1560*g*, 10 min at 4 °C) and kept at −80 °C until assayed for TXB_2_, measured as a reflection of maximally stimulated platelet COX-1 activity in response to endogenously formed thrombin. TXB_2_ levels were measured by previously described and validated enzyme-immunoassay (EIA).^62,63^

#### Ex Vivo Inhibition of Platelet Aggregation

C57BL/6 male mice (12–13 weeks old) supplied by Charles River, housed in a temperature-controlled, 12 h light/dark cycle environment with ad libitum access to water and fed on standard pellet diet, were randomized in different groups. Compounds were dissolved in Carboxymethylcellulose (CMC) 1% at the concentrations of 5 mg/mL **51** and 20 and 40 mg/mL **50** and vortexed to attain complete suspension. Mice were immediately treated orally by gavage with **51** (50 mg/kg), **50** (200 mg/kg and 100 mg/kg) or vehicle (CMC). After 2 h, mice were anesthetized with ketamine hydrochloride (75 mg/kg; Intervet) and medetomidine (1 mg/kg; Virbac) and blood was collected into 3.8% sodium citrate (1:10 v/v) by cardiac venipuncture, pooled and immediately centrifuged at 350 *g* for 15 min at RT in order to obtain platelet-rich plasma. All experiments were approved and authorized by the National Ministry of Health—University of Milan Committee (approval number 12/12-30012012).

#### Docking & Computational Studies

For molecular docking, Molecular Operating Environment software (MOE 2019.0102, Chemical Computing Group, Montreal, Canada) was used. The PDB files of the complex structures (COX-2:diclofenac—1PXX, TP:ramotraban—6IIU) were loaded and subsequently prepared for docking using QuickPrep routine with default settings. The binding site was defined around the cocrystallized ligands and the acidic headgroup of the cocrystallized ligands was used for the initial placement inside Phamacophore Query and London dG scoring function. After generation of 30 poses per ligand, refinement was performed with flexible receptor settings using GBVI/WSA dG scoring function. Top 5 poses were remained and the highest scored pose was used for visualization.

#### PK Studies

A total number of 3 adult male RjOrl:Swiss (CD-1) mice were housed in a temperature-controlled room (20–24 °C) and maintained in a 12 h light/12 h dark cycle. Food and water were provided ad libitum. All experimental procedures were approved by and conducted in accordance with the regulations of the local Animal Welfare authorities (Landesamt für Gesundheit and Verbraucherschutz, Abteilung Lebensmittel- and Veterinärwesen, Saarbrücken). At each time point a volume of 100–150 μL Li-heparin whole blood, i.e. 40–50 μL Li-heparin plasma, was collected from the retrobulbar venous plexus under short isoflurane anesthesia. The plasma samples were prepared within 45 min after collection, frozen at −20 °C and stored at this temperature until processed for LC–MS analysis. The PK studies were performed by the CRO Pharmacelsus GmbH under the protocol number 2021UFM002.

#### TP Antagonism in Rat Aorta

All experiments were performed in accordance with Italian and International Guidelines (DL 26/2014 implementation of directive 2010/63 UE). The experimental protocol was approved by the Turin University Bioethical Committee and the Italian Ministry of Health (approval n. 56105.N.WSP). Male Wistar rats were anaesthetized with isoflurane, decapitated and exsanguinated. The aorta was removed immediately, dissected free of fat and connective tissue and cut into rings of about 3–4 mm in width. Endothelium-intact rings were mounted in organ baths containing 30 mL of Krebs–Ringer Bicarbonate Buffer (KRB) with the following composition (mM): NaCl 111.2, KCl 5.0, CaCl_2_ 2.5, MgSO_4_ 1.2, KH_2_PO_4_ 1.0, NaHCO_3_ 12.0, glucose 11.1, maintained at 37 °C and gassed with 95% O_2_ −5% CO_2_ (pH 7.4). The aortic rings were mounted on lower and upper organ hooks, connected to the isometric force–displacement transducers, under 1.0 g tension. Changes in smooth-muscle tension in the preparations, that is vascular smooth-muscle contractions and relaxations, were recorded and displayed by a computerized data acquisition system connected to a “Power MacLab” Bridge Amplifier. The rings were allowed to equilibrate for 60 min and endothelium integrity was assessed with 10 μM acetylcholine (ACh) in rings precontracted by 1 μM phenylephrine. A relaxation ≥ 75% of phenylephrine-induced tone was considered sign of functional endothelium. Aortic rings were then washed and equilibrated for another 60 min period. After a pretreatment for 20 min with 10 μM indomethacin, cumulative concentration–response curves for U-46619 were established in control or in the presence of each compound, added to the organ bath fluid 20 min before the concentration–response curves for U-46619 was determined. All responses were expressed as percent of the maximal contraction induced by KCl 50 mM, and IC_50_ values were calculated by nonlinear regression analysis. For each inhibitor, *pA*_2_ value was calculated with Gaddum equation: *pA*_2_ = log­[CR – 1] – log­[*B*], where CR = ratio of EC_50_ with and without antagonist; [*B*] = antagonist concentration. At least three experiments for each compound were performed.

#### TP Antagonism in Platelet-Rich Plasma

Venous blood samples were obtained from healthy volunteers who had not taken any platelet inhibitory drug for at last 1 week. Volunteers were treated according to Helsinki protocol for biomedical experimentation after approval by the local Ethics Committee of “Università degli Studi di Torino” and gave their informed consent to the use of blood samples for research purposes. To study the antagonism to the thromboxane receptor, human blood was anticoagulated with citrate solution and treated with acetylsalicylic acid (10 μg/mL). Platelet rich plasma (PRP) was prepared by centrifugation of citrated blood at 200*g* for 20 min. Aliquots (500 μL) of PRP were added into aggregometer (Chrono-log 4902D) cuvettes and aggregation was recorded as increased light transmission under continuous stirring (1000 rpm) at 37 °C for 10 min after addition of the stimulus. Compounds under study were preincubated with PRP 10 min before addition of the agonist U-46619 (0.5–2.5 μM). Vehicle alone (0.5% DMSO) was added to PRP to verify platelet function in control samples. At least three experiments for each compound have been performed. The antiaggregatory activity of tested compounds was evaluated as % inhibition of platelet aggregation compared to control samples. IC_50_ values were calculated by nonlinear regression analysis.

#### Solubility of 1, 21, 25, 32, 49, 50, and 51 in PBS (pH 7.4)

Selected compounds (2 mg) were added to 1 mL of phosphate buffered saline (PBS: 137 mM NaCl, 2.7 mM KCl, 10 mM Na_2_HPO_4_, and 1.8 mM KH_2_PO_4_, pH 7.4). The samples were shaken with an orbital shaker at 25 °C for 24 h. These suspensions were filtered through a PTFE 0.45 μm filter (VWR) and the solutions were analyzed by HPLC. HPLC analysis were performed with a HP 1100 chromatograph system (Agilent Technologies, Palo Alto, CA, USA) equipped with a quaternary pump (model G1311A), a membrane degasser (G1379A), a diode-array detector (DAD) (model G1315B) integrated in the HP1100 system. Data analysis were processed using a HP ChemStation system (Agilent Technologies). The analytical column was a ZORBAX SB-Phenyl (250 × 4.6 mm, 5 μm; Agilent). The samples were analyzed using an isocratic method employing a mobile phase consisting of acetonitrile/water + HCOOH 0.1%, 65/35 (v/v) at flow rate = 1.0 mL/min. The injection volume was 20 μL (Rheodyne, Cotati, CA). The column effluent was monitored at 226 and 280 nm referenced against 800 nm wavelength. Quantitation was done using calibration curves of compounds; the linearity of the calibration curves was determined in a concentration range of 1–200 μM (*r* ^2^ > 0.99).

#### Determination of Dissociation Constants and Lipophilicity Descriptors

The ionization constants of compounds were determined by potentiometric titration with the GLpKa apparatus (Sirius Analytical Instruments Ltd., Forest Row, East Sussex, UK) following a previously described procedure.^54^ Calculated partition coefficient of compounds in neutral form (*C* log *P*) werexy obtained by using Bio-Loom for Windows v.1.5 (BioByte Corp. Claremont, CA, USA). The distribution coefficient at pH 7.4 (log *D* ^7.4^) of the compounds between *n*-octanol and phosphate buffer was experimentally obtained by shake-flask technique at room temperature according to the published procedure.^54^

#### In Vitro Plasma Protein Binding

The amount of compounds bound to serum proteins was determined by ultrafiltration. Briefly, a solution of selected compound in DMSO was added to human serum (sterile-filtered from human male AB plasma, Sigma-Aldrich), to obtain 200 μM final concentration (2% of cosolvent); 1 mL of this solution was inserted in the sample reservoir of the ultrafiltration device (Centrifree ultrafiltration devices with ultracel YM-T membrane, Merck) and gently shaken in an orbital shaker at 37 °C for 1 h. The tube was then centrifuged at 1000*g* for 15 min. The concentration of the compound in the ultrafiltrate and filtrate was determined using RP-HPLC in the chromatographic conditions described above. The quantitation of the compound in the filtrate was performed using the calibration curves (linearity determined in a concentration range of 1–200 μM; *r* ^2^ > 0.99); the quantitation of compound in the ultrafiltrate was performed using calibration curves (linearity determined in a concentration range of 0.2–10.0 μM; *r* ^2^ > 0.99). The recovery of the ultrafiltration process was calculated to check whether any compound was lost during ultrafiltration. Medium recovery was 98% for all tested compounds.

#### Animals

Experiments were performed in 6–12 week-old male and female C57BL/6N mice (Charles River, Sulzfeld, Germany). Animals were housed on a 12 h light/dark cycle with access to food and water *ad libitum*. The studies were performed by an observer blinded for treatment of the animals. All experiments adhered to the guidelines of the International Association for the Study of Pain and to the ARRIVE (Animals in Research: Reporting In Vivo Experiments) guidelines, and were approved by our local Ethics Committee for Animal Research (Regierungspräsidium Darmstadt, Germany, approval number V54-19c20/15-FR/1004).

#### Drugs

Compound **51** and diclofenac (Sigma-Aldrich, Darmstadt, Germany) were suspended at a concentration of 50 mg/mL in phosphate buffered saline (PBS) containing 3% methylcellulose (Methocel A15LV, Sigma-Aldrich, Darmstadt, Germany). To improve the solubility, the mixtures were vortexed for 5 min immediately before administration.

#### CFA-Induced Inflammatory Pain

Mechanical sensitivity of a hindpaw was assessed using a dynamic plantar aesthesiometer (Ugo Basile, Comerio, VA, Italy). Animals were placed on a wire mesh grid and habituated to the apparatus chamber for 1 h. A thin probe (0.5 mm diameter) was applied against the plantar surface of the paw from beneath with increasing force from 0 to 5 g within 10 s and a constant force of 5 g for additional 10 s until a strong withdrawal occurred.^67^ The paw withdrawal latency was recorded automatically and calculated as the average of 6–8 measurements. After baseline measurements, 20 μL of Complete Freund’s adjuvant (CFA, containing 1 mg/mL heat-killed *Mycobacterium tuberculosis* in paraffin oil 85% and mannide monooleate 15%; Sigma-Aldrich, Darmstadt, Germany) was injected into the plantar surface of the hindpaw.^66^ Mechanical sensitivity was again determined 24 h after the CFA injection. Immediately thereafter, drugs or vehicle were administered by oral gavage and the mechanical sensitivity was determined over 2 h.

#### Statistical Analysis

Statistical analysis was performed using Prism 9 (GraphPad). Data were analyzed using two-way repeated measures ANOVA followed by Dunnett’s *post hoc* test for multiple comparisons. Concentration–response curves were analyzed using the four-parameter logistic model. Parameter errors are all expressed in percentage coefficient of variation (% CV). Data are presented as the mean ± standard error of the mean (SEM) and a probability value *p* < 0.05 was considered as statistically significant.

## Supplementary Material











## Notes

HPLC chromatograms, NMR and HRMS spectra of compounds **21**, **25**, **32**, **48**, **50** and **51** (PDF)
21_TP (PDB)
51_COX2 (PDB)
51_TP (PDB)
Molecular formula strings (CSV)

## ○

Marta Balestra: Sorbonne Université, École normale supérieure, PSL Université, CNRS, Laboratoire de Chimie Physique et Chimie du Vivant (CPCV), 75005, Paris; Maria Luisa Introvigne: Dipartimento di Scienze della Vita, Università di Modena e Reggio Emilia, Via Campi 287, 41125 Modena

## Notes

This article is dedicated to the memory of our dear friend and colleague Enrico Rovati, who passed away prematurely in September 2023.

## Abbreviations

AA: arachidonic acid
CFA: complete Freund’s adjuvant
CMC: carboxymethylcellulose
COX: cyclooxygenase
COXTRANs: COX inhibitors
DMSO: dimethyl sulfoxide
GPCRs: G protein-coupled receptors
HBSS: Hank’s balanced salt solution
KRB: Krebs–Ringer bicarbonate buffer
LPS: lipopolysaccharide
NSAIDs: Nonsteroidal anti-inflammatory drugs
PBS: phosphate buffered saline
PG: prostaglandin
PK: pharmacokinetics
PRP: platelet-rich plasma
TP: thromboxane receptor
TX: thromboxane
UGIB: upper gastrointestinal bleeding
