# Supplementary material for: Exploiting the 2‑(1,3,4,9-tetrahydropyrano[3,4‑b]indol-1-yl)acetic Acid Scaffold to Generate COXTRANs: A New Class of Dual Cyclooxygenase Inhibitors-Thromboxane Receptor Antagonists
Source: J Med Chem. 2025 Oct 22;68(21):23185–219. doi: 10.1021/acs.jmedchem.5c02068 (PMC12621201; doi:10.1021/acs.jmedchem.5c02068)

Supporting Information

Exploiting the 2-(1,3,4,9-tetrahydropyrano[3,4-b]indol-1-yl)acetic acid scaffold to generate COXTRANs: a new class of dual cyclooxygenase inhibitors-thromboxane receptor antagonists

Federica Blua,[†,‖] Francesca Boccato,[†,‖] Carola Buccellati,[‡] Patrizia Risè, [‡] Silvia Barbieri,[¥] Laura Castiglioni,[‡] Annika Balzulat,[Ψ] Barbara Rolando,[†] Elisabetta Marini,[†] Marta Balestra,[†] Maria Luisa Introvigne,[†] Marta Giorgis,[†] Luigi Sironi,[‡] Stefania Tacconelli, [§,¶] Kerstin Hiesinger,[#] Paola Patrignani,[§,¶] Achim Schmidtko,[Ψ] Dieter Steinhilber,[#] Ewgenij Proschak,[#] Angelo Sala,[‡] G. Enrico Rovati,[‡] and Massimo Bertinaria[†,*]

†Department of Drug Science and Technology, University of Turin, Via P. Giuria 9 – 10125 Torino, Italy.

#Institute of Pharmaceutical Chemistry, Goethe-University, Max-von-Laue-Str. 9, D-60438 Frankfurt am Main, Germany.

§ Department of Neuroscience, Imaging and Clinical Sciences, "G. d'Annunzio" University, 66100 Chieti, Italy.

‡ Department of Pharmaceutical Sciences, University of Milan, 20122 Milan, Italy.

¶ Laboratory of Systems Pharmacology and Translational Therapies, Center for Advanced Studies and Technology (CAST), "G. d'Annunzio" University, 66100 Chieti, Italy.

¬ Institute of Pharmacology and Clinical Pharmacy, Goethe University, Max-von-Laue-Str. 9, D-60438 Frankfurt am Main, Germany.

¥ Centro Cardiologico Monzino IRCCS, Milan, Italy

Corresponding Author

Massimo Bertinaria, Email: Massimo.bertinaria@unito.it

Table of Contents

Protocol for assessing the purity of final compounds…………………………………………………………………………….S3

HPLC trace for purity of selected final compounds…………………………………………………………………………S4-S9

^{1}H and ^{13}C NMR spectra of selected final compounds……………………………………………………………..S10-S15

HRMS spectra of selected final compounds………………………………………………………………………………………S16

Determination of purity by HPLC.

The purity of target compounds was assessed by RP-HPLC. Analyses were performed on a HP1100 chromatograph system (Agilent Technologies, Palo Alto, CA, USA) equipped with a quaternary pump (G1311A), a membrane degasser (G1379A), a diode-array detector (DAD) (G1315B) integrated in the HP1100 system. Data analyses were processed by HP ChemStation system (Agilent Technologies). The analytical columns were LiChrospher® 100 C18-e (250×4.6mm, 5µm) (Merck KGaA, 64271 Darmstadt, Germany) and Zorbax SB-Phenyl (4.6 × 250 mm, 5 µm, Agilent Technologies) eluted with CH_3CN 0.1% TFA (solvent A) and water 0.1% TFA (solvent B) in gradient mode (from 40% to 100% A on 20 minutes). All compounds were dissolved in the mobile phase at a concentration of about 0.3 mg/mL and injected through a 20 µL loop (Rheodyne, Cotati, CA). HPLC retention times (t_R) were obtained at flow rates of 1.0 mL/min, and the column effluent was monitored at 210, 226 and 254 nm referenced against 800 nm.

The purity of the compounds was evaluated as a percentage ratio between the areas of the main peak and of possible impurities at the three wavelengths and also using DAD purity analysis of the chromatographic peak.

Compound **21**_ LiChrospher® 100 C18-e; t_R = 12.6 min, %purity = 100%

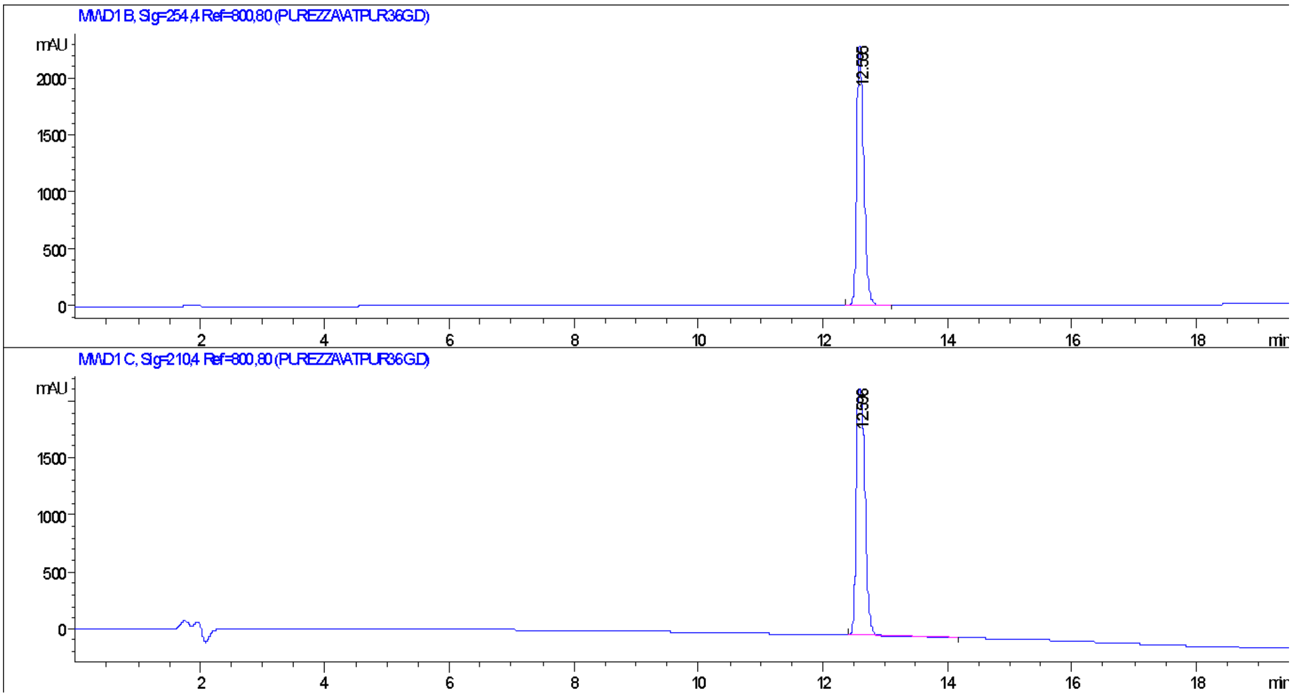

Compound **21**_ Zorbax SB-Phenyl; t_R = 10.5 min, %purity = 100%

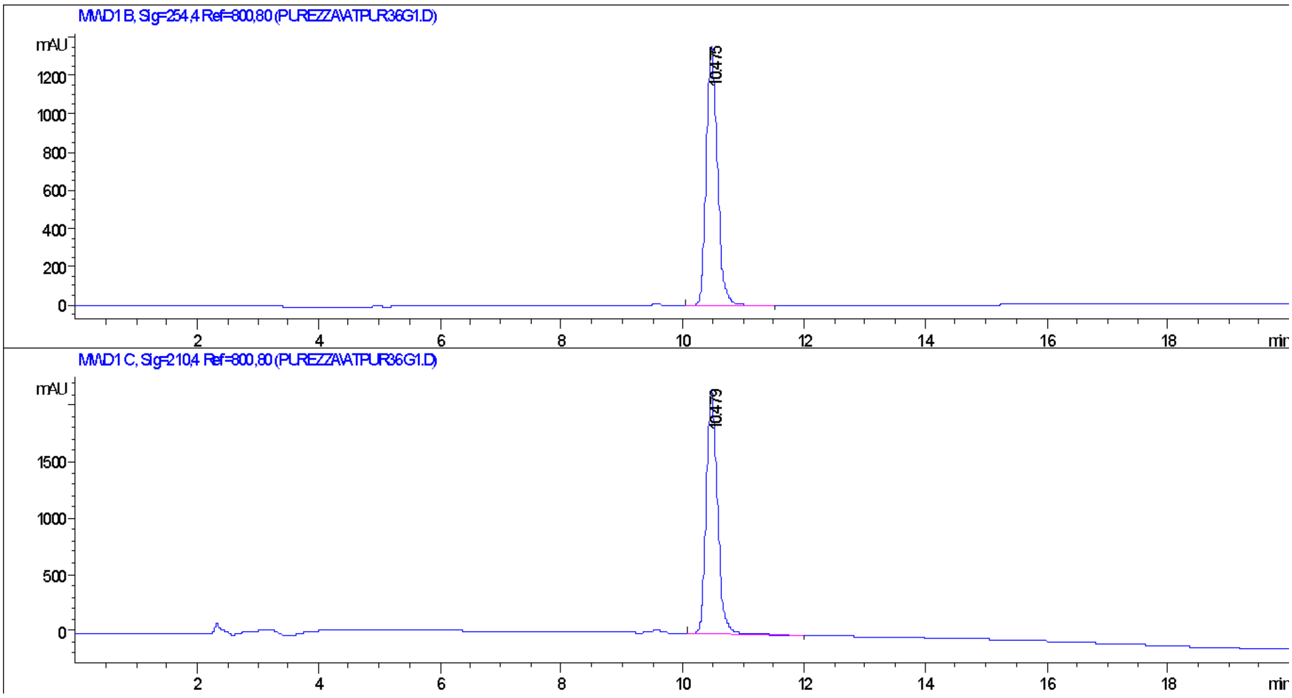

Compound **25**_ LiChrospher® 100 C18-e; t_R = 14.3 min, %purity = 96%

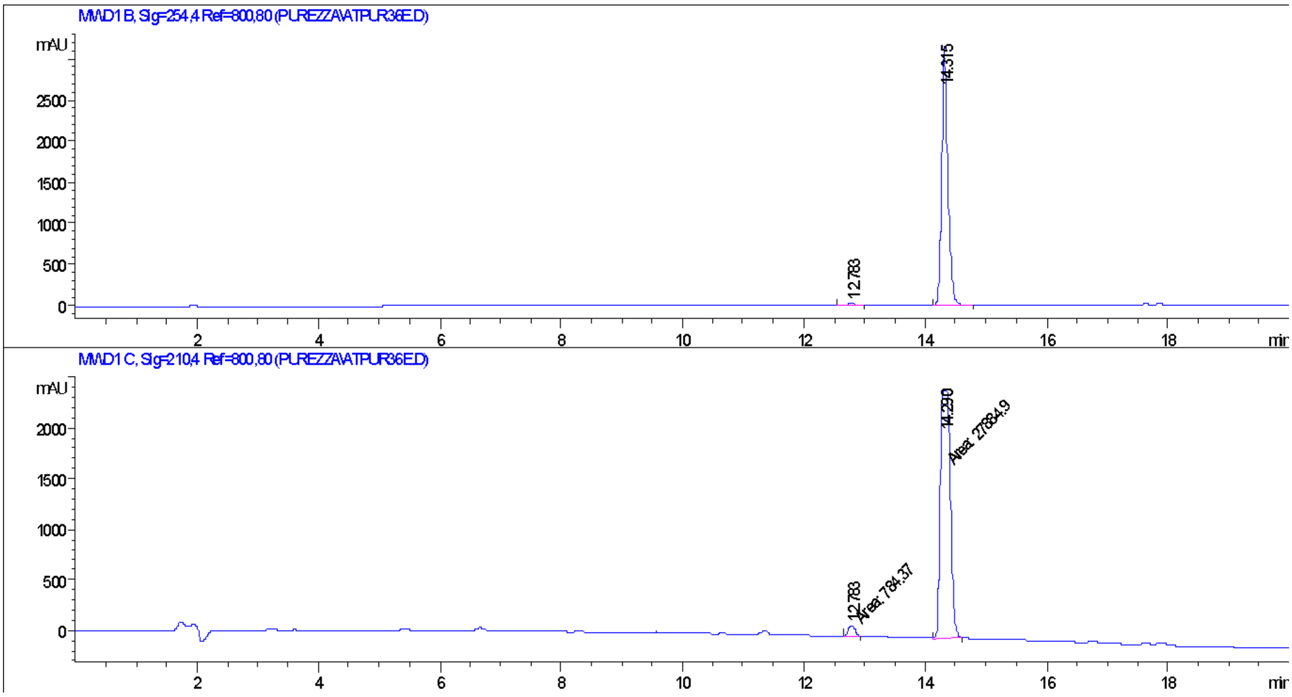

Compound **25**_Zorbax SB-Phenyl; t_R = 11.7 min, %purity = 96%

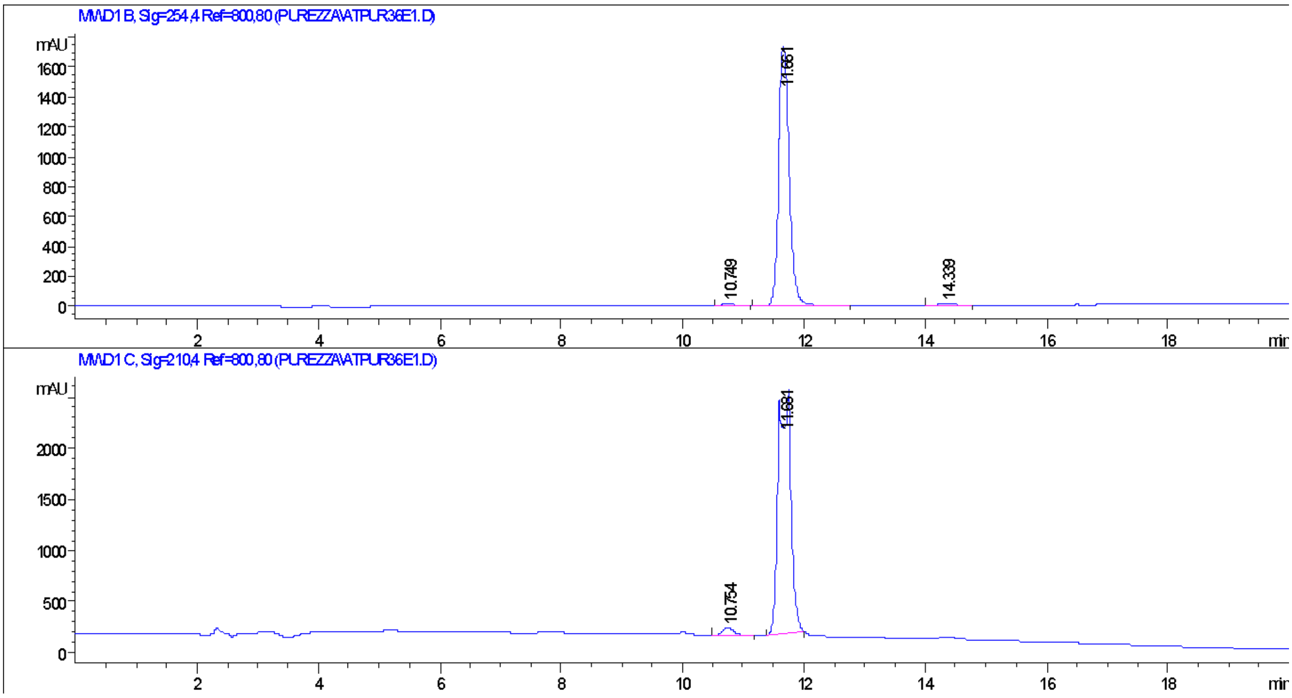

Compound **32**_ LiChrospher® 100 C18-e; t_R = 9.5 min, %purity = 98%

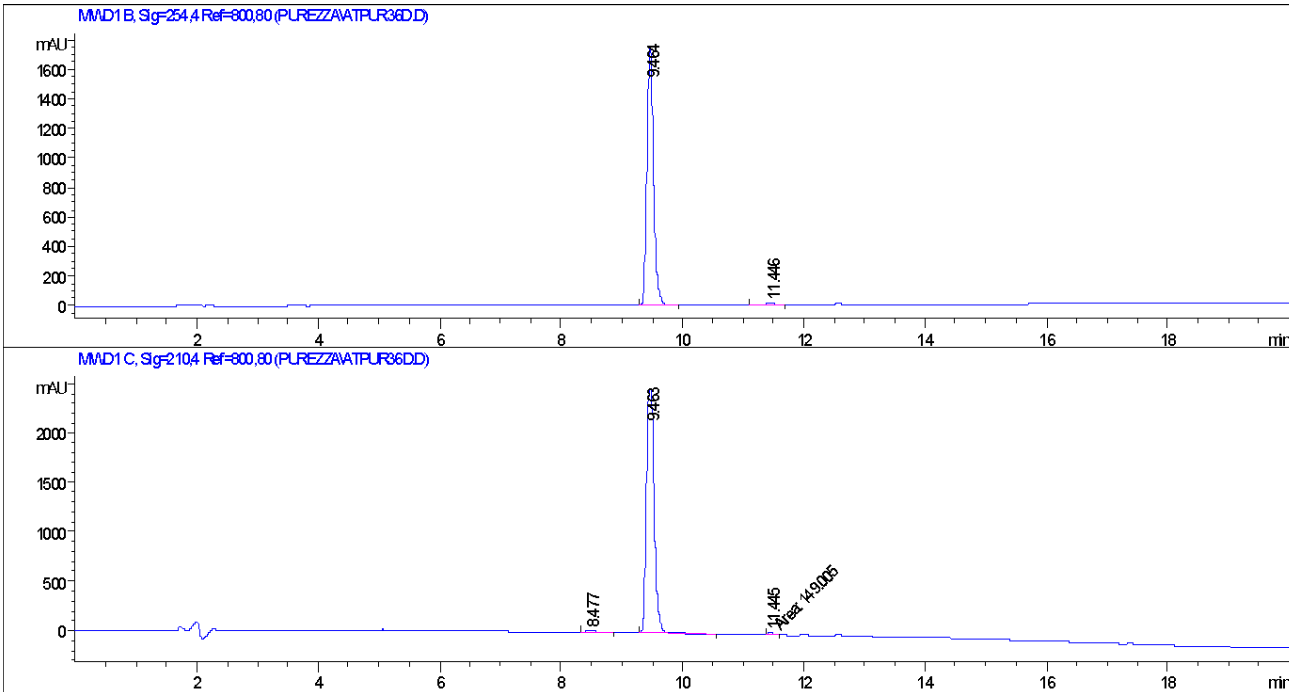

Compound **32**_ Zorbax SB-Phenyl; t_R = 7.7 min, %purity = 98%

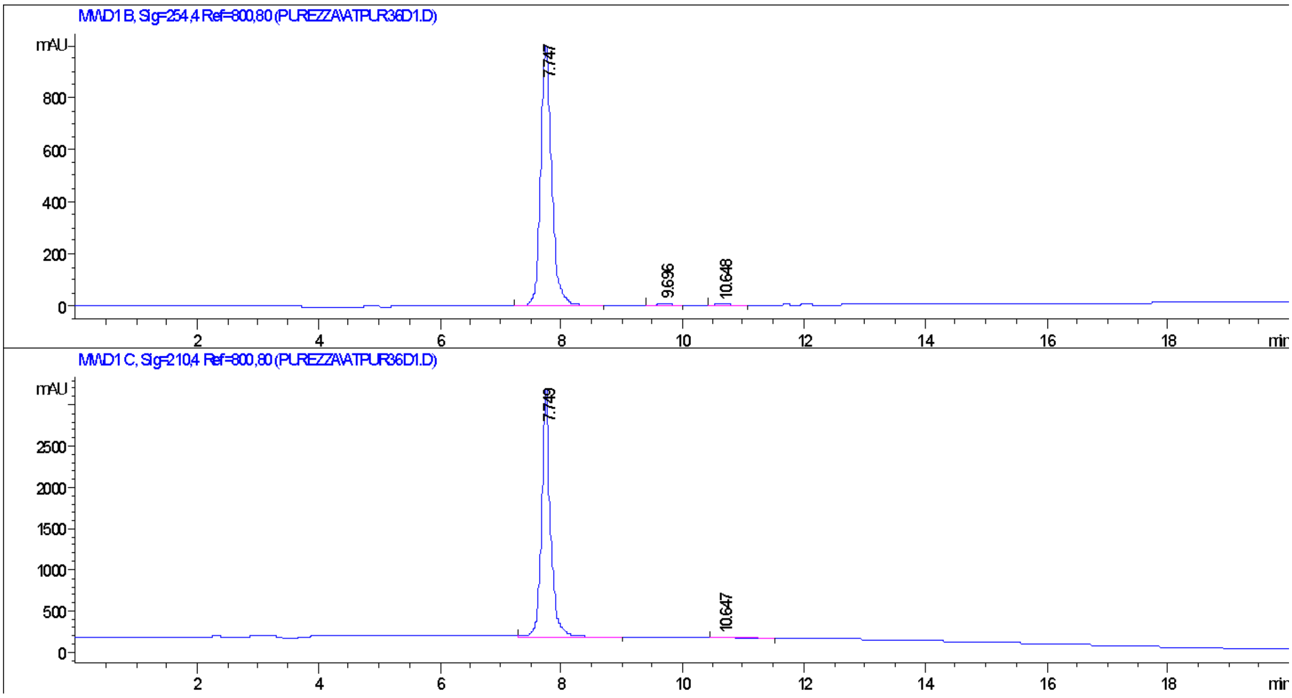

Compound **48**_ LiChrospher® 100 C18-e; t_R = 9.0 min, %purity = 100%

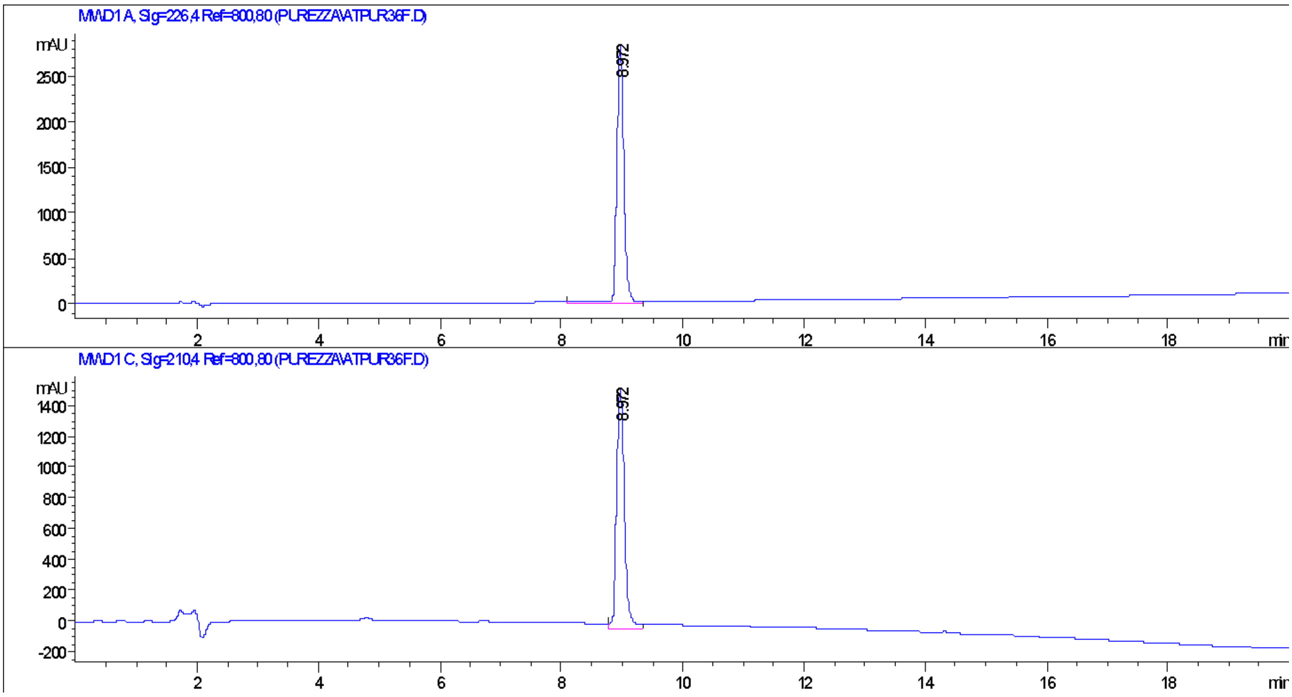

Compound **48**_ Zorbax SB-Phenyl; t_R = 7.7 min, %purity = 100%

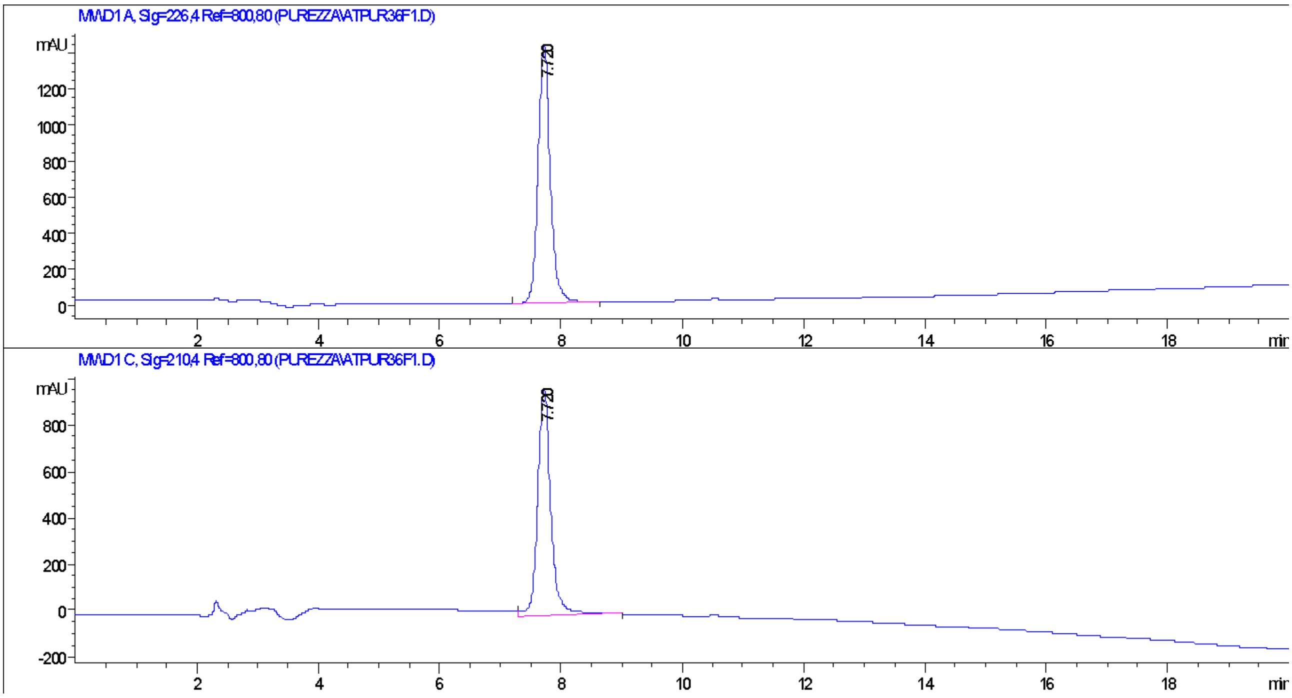

Compound **50**_LiChrospher® 100 C18-e; t_R = 11.3 min, %purity = 100%

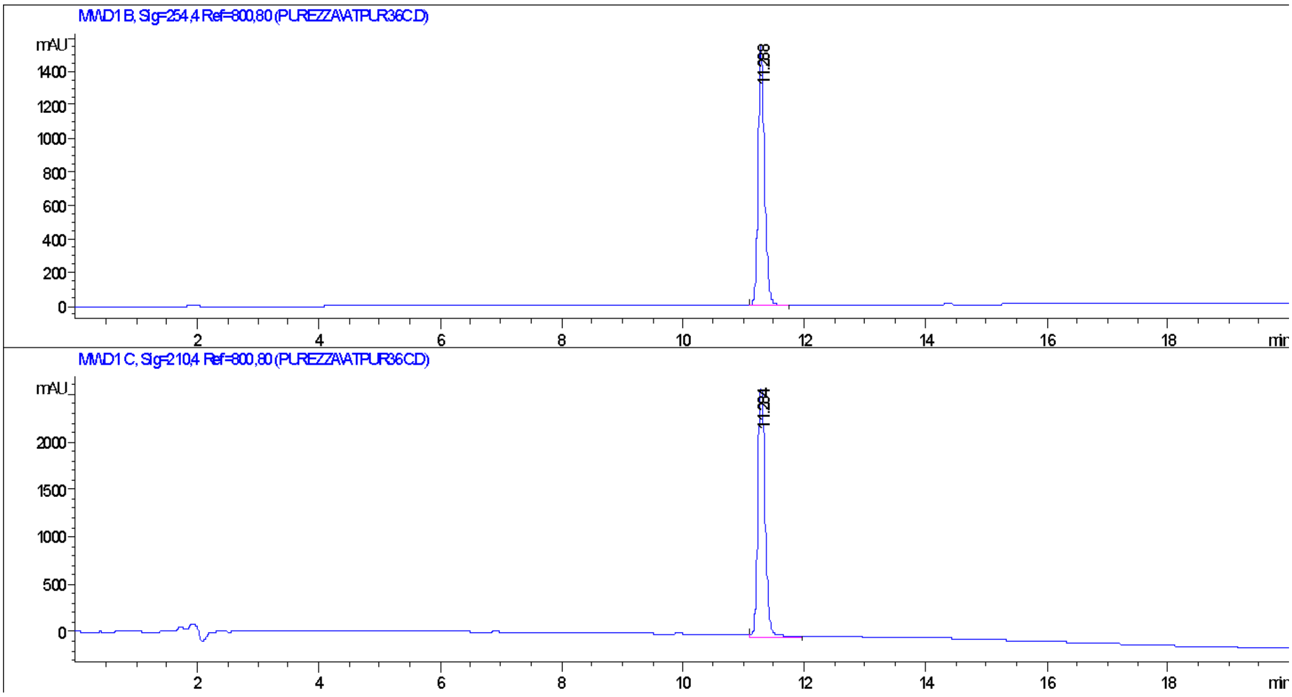

Compound **50**_ Zorbax SB-Phenyl; t_R = 10.4 min, %purity = 100%

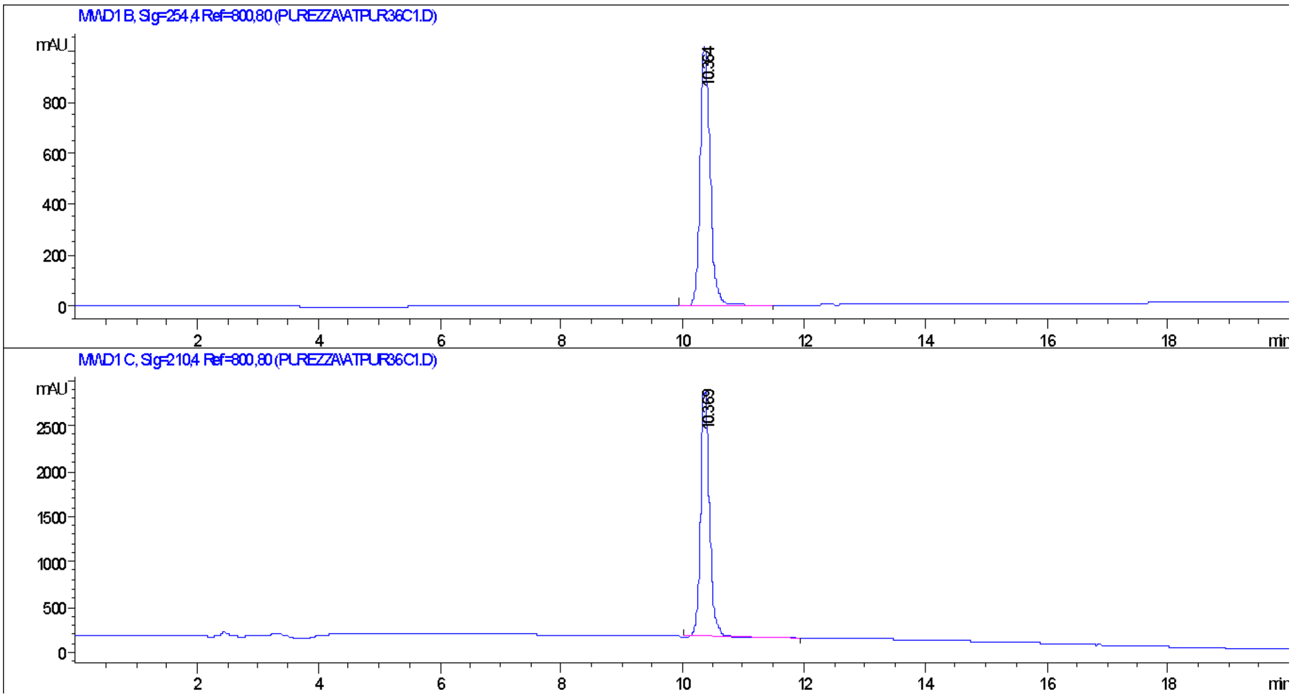

Compound **51**_ LiChrospher® 100 C18-e; t_R = 12.1 min, %purity = 97%

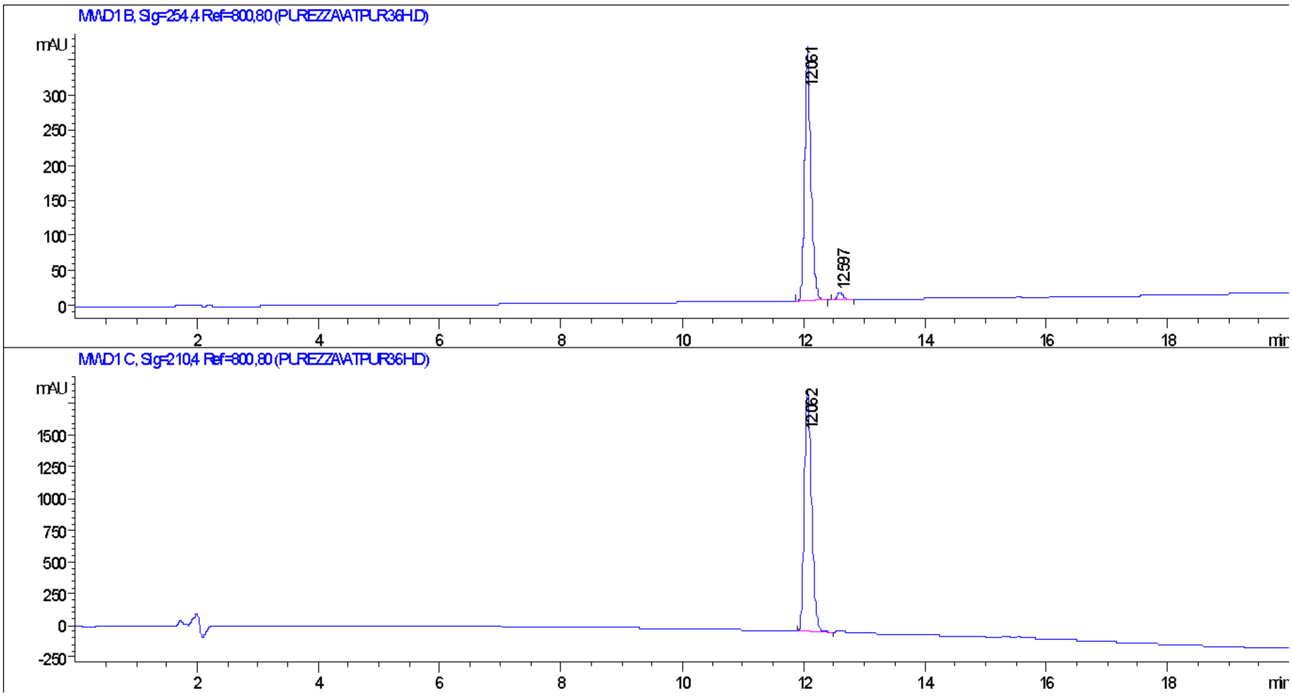

Compound **51**_ Zorbax SB-Phenyl; t_R = 10.5 min, %purity = 100%

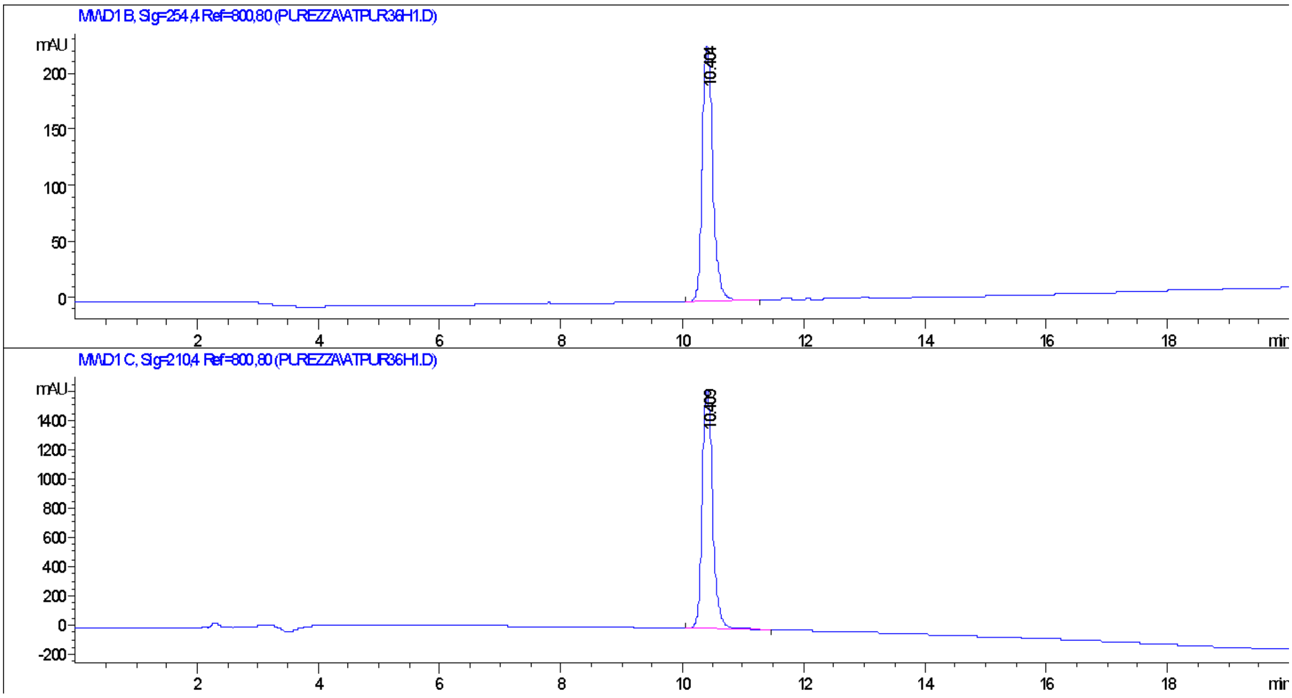

^{1}H and ^{13}C NMR spectra of selected final compounds.

^{1}H and ^{13}C NMR spectra were registered on Bruker Avance 300 spectrometer, at 300 and 75 MHz respectively, or on Jeol ECZ 600 M30 at 600 or 150 MHz respectively. Chemical shifts (δ) are given in ppm, calibrated to tetramethylsilane (TMS) or to solvent signal as internal standard.

Compound **21**_ ^{1}H NMR (300 MHz, $(CD_3)_2CO$).

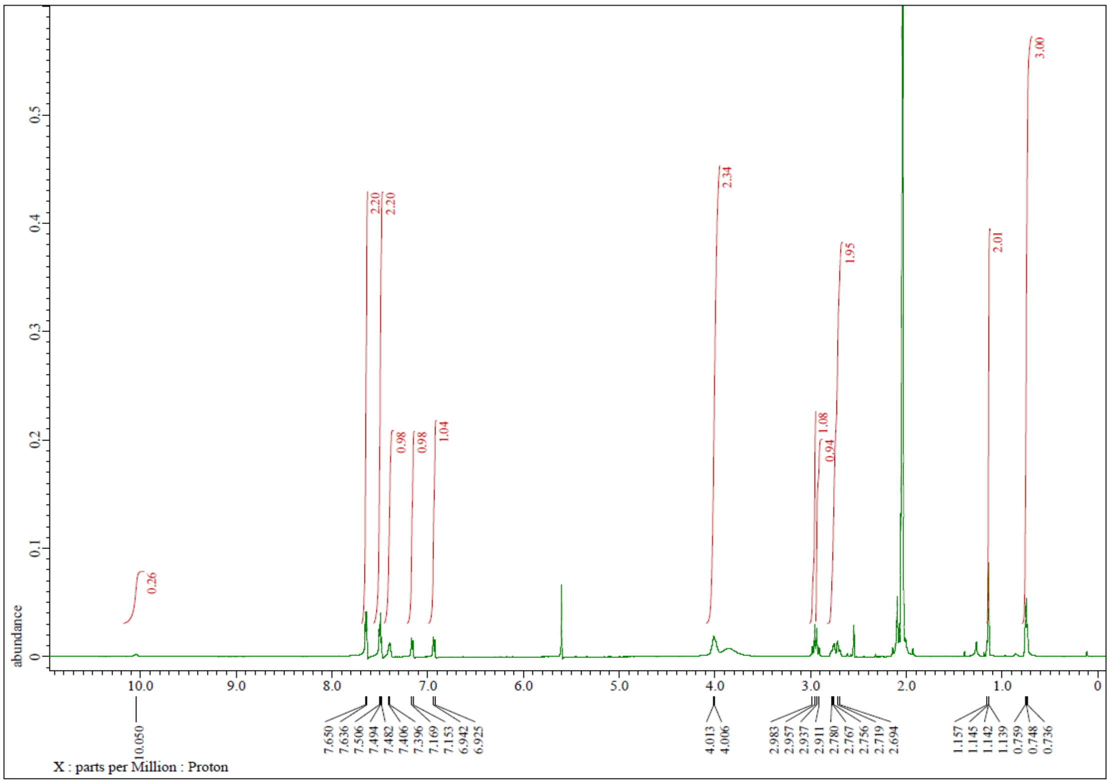

Compound **21**_ ^{13}C NMR (75 MHz, $(CD_3)_2CO$).

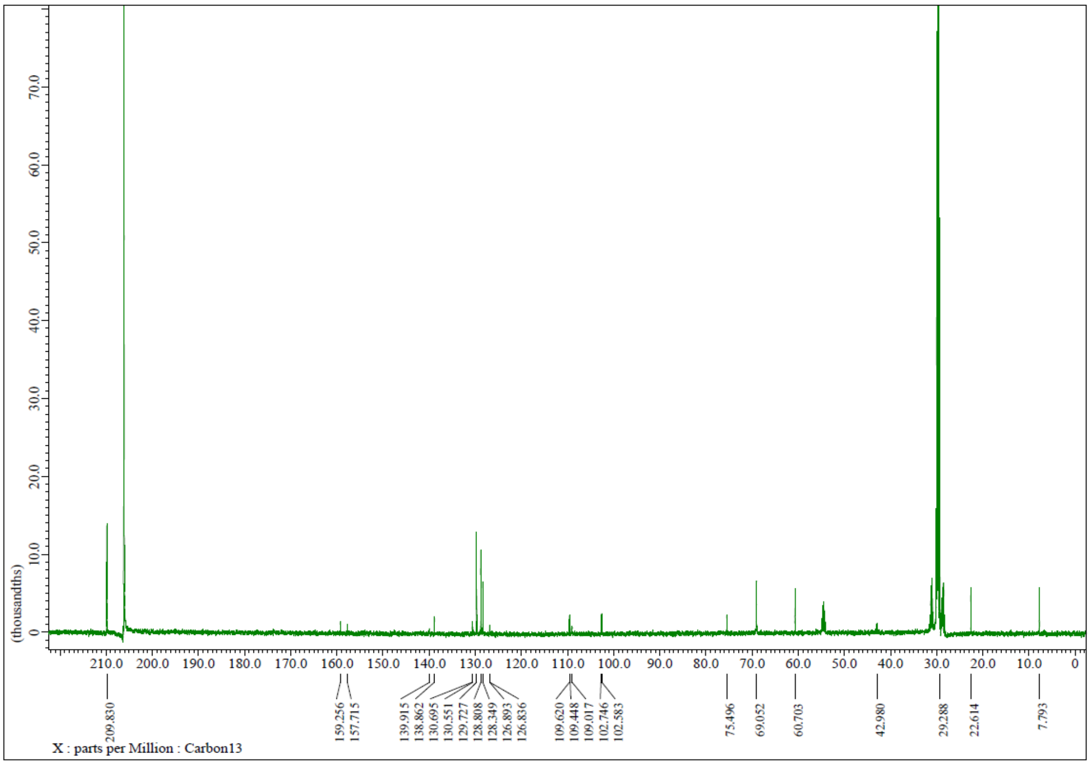

Compound **25**_ ^{1}H NMR (300 MHz, DMSO-d$_6$).

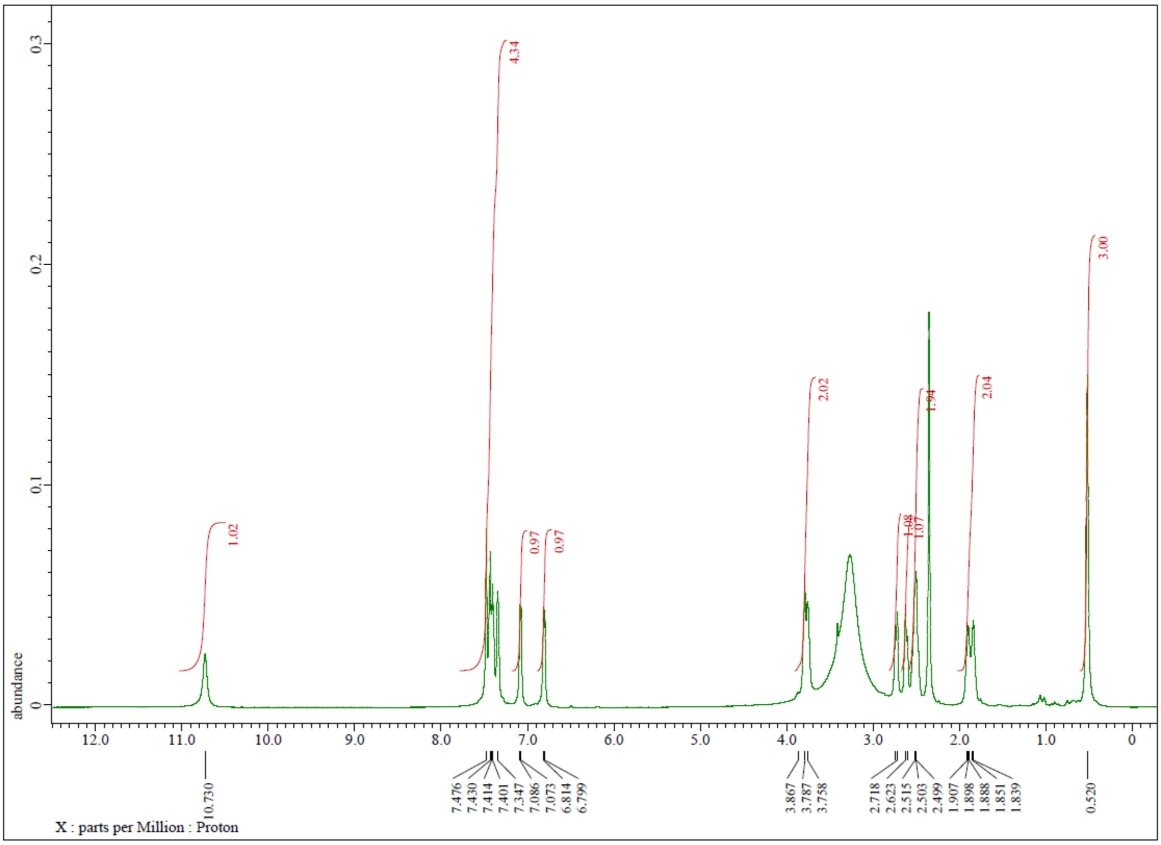

Compound **25**_ ^{13}C NMR (151 MHz, DMSO-d$_6$).

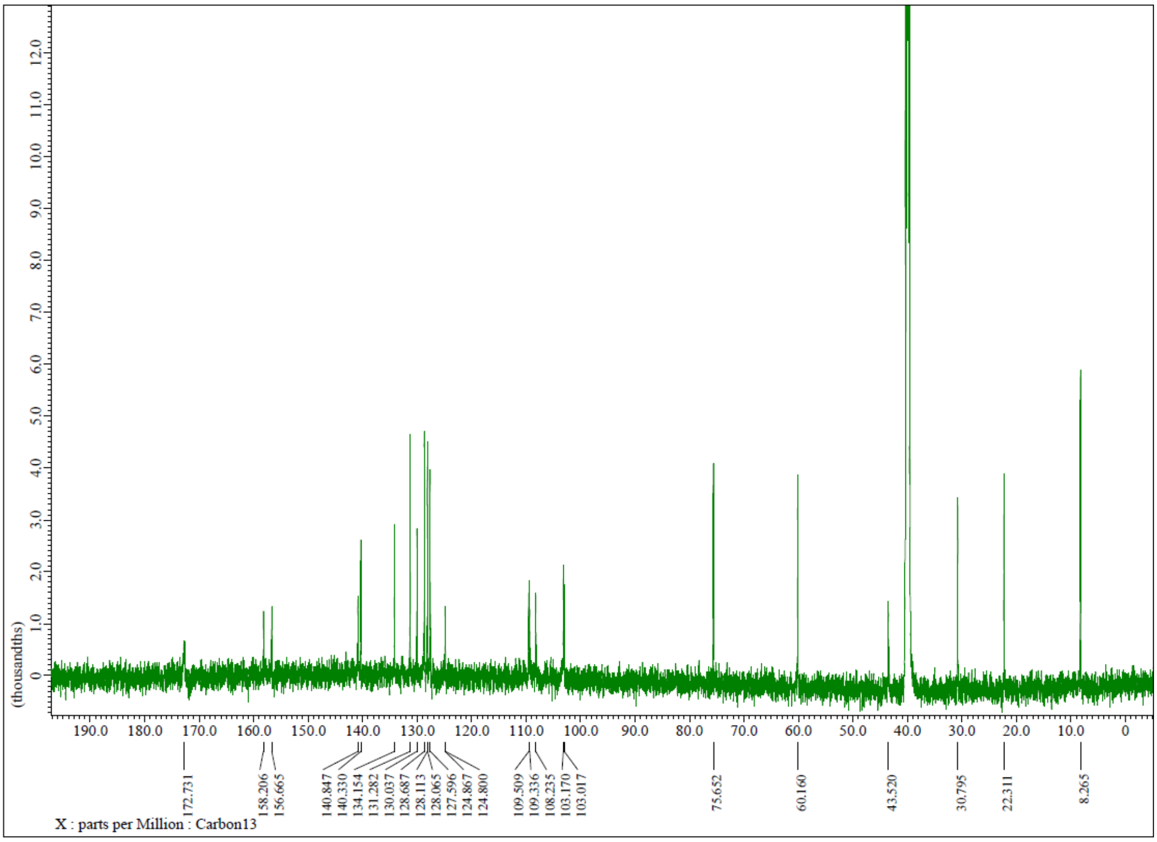

Compound **32**_ ^{1}H NMR (600 MHz, DMSO-d_6).

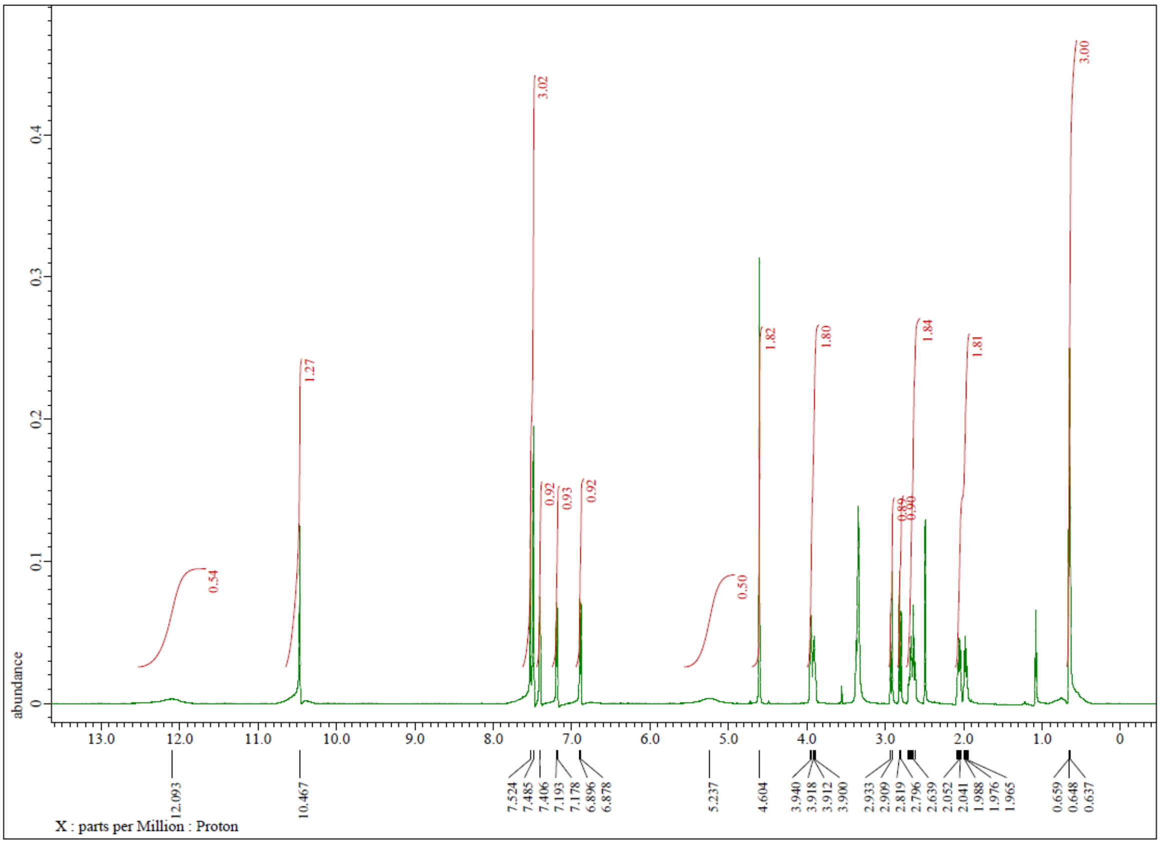

Compound **32**_ ^{13}C NMR (151 MHz, DMSO-d_6).

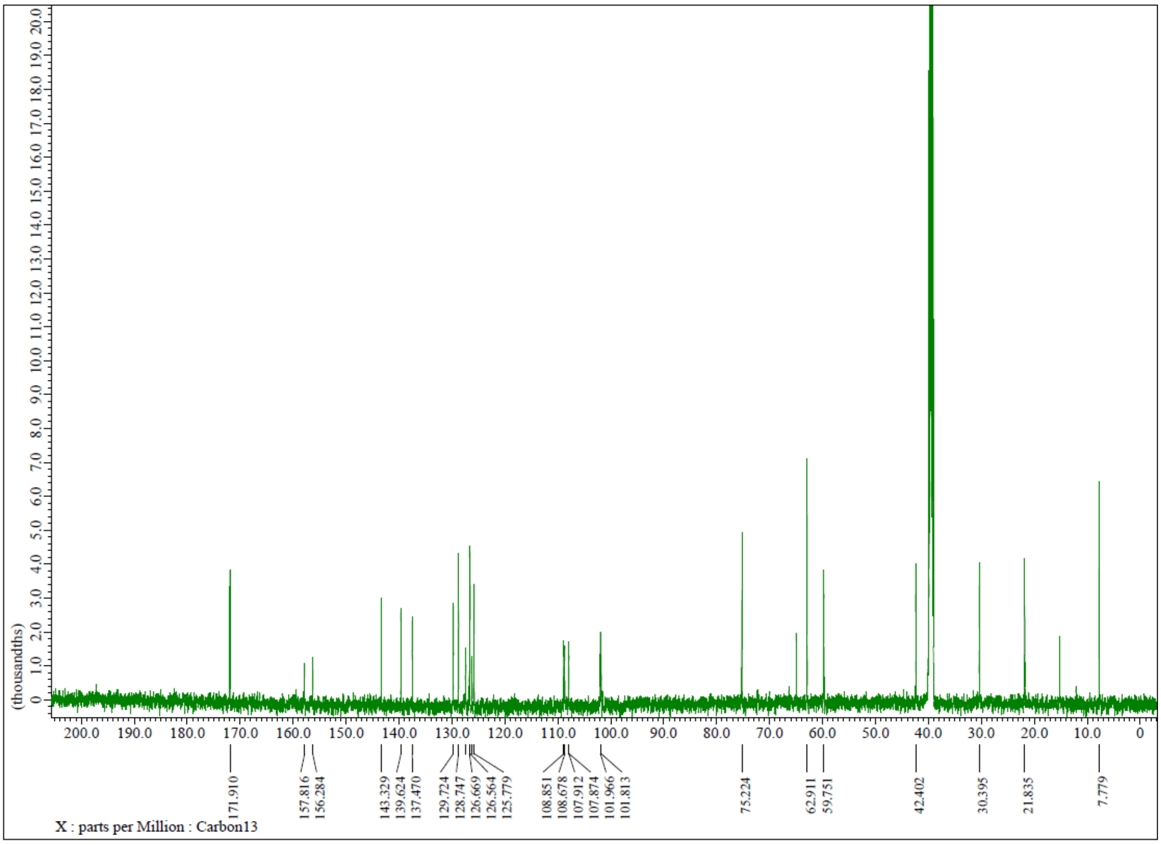

Compound **48**_ ^{1}H NMR (600 MHz, DMSO-d$_6$).

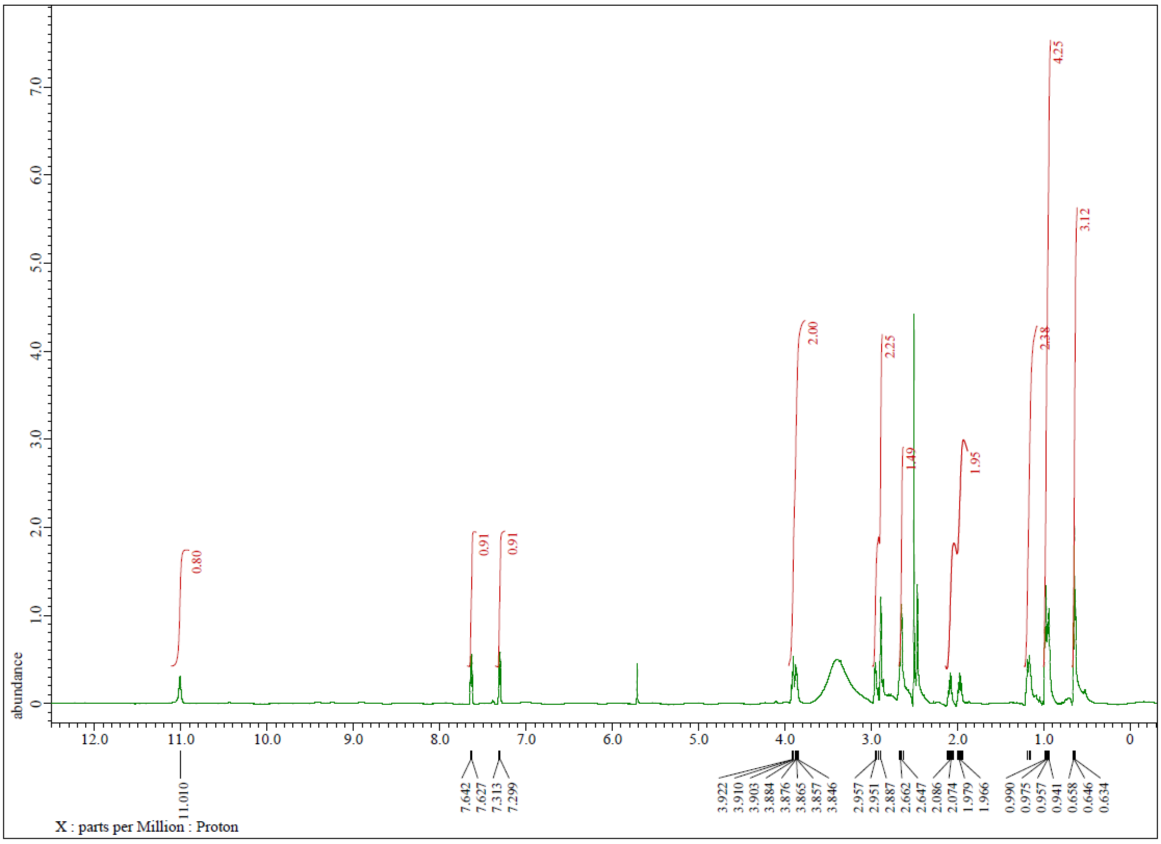

Compound **48**_ ^{13}C NMR (151 MHz, DMSO-d$_6$).

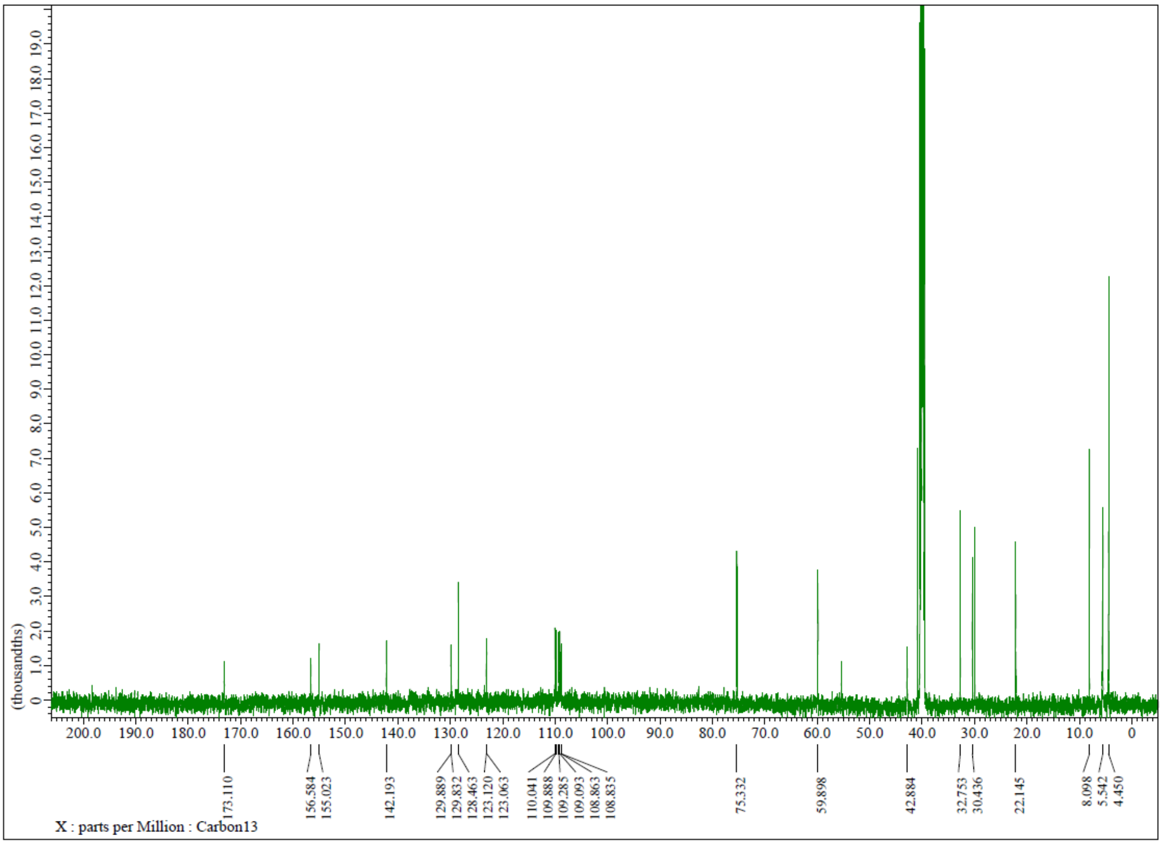

Compound **50**_ ^{1}H NMR (300 MHz, DMSO-d_6).

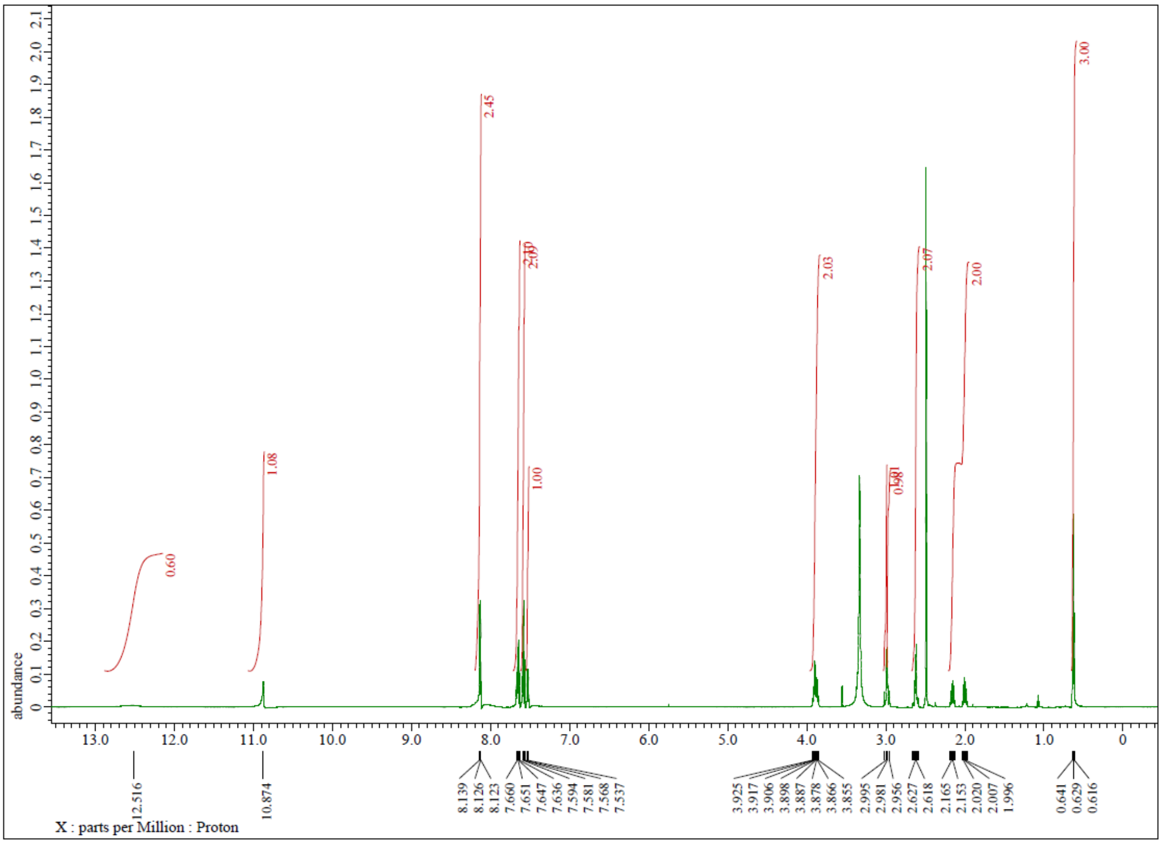

Compound **50**_ ^{13}C NMR (151 MHz, DMSO-d_6).

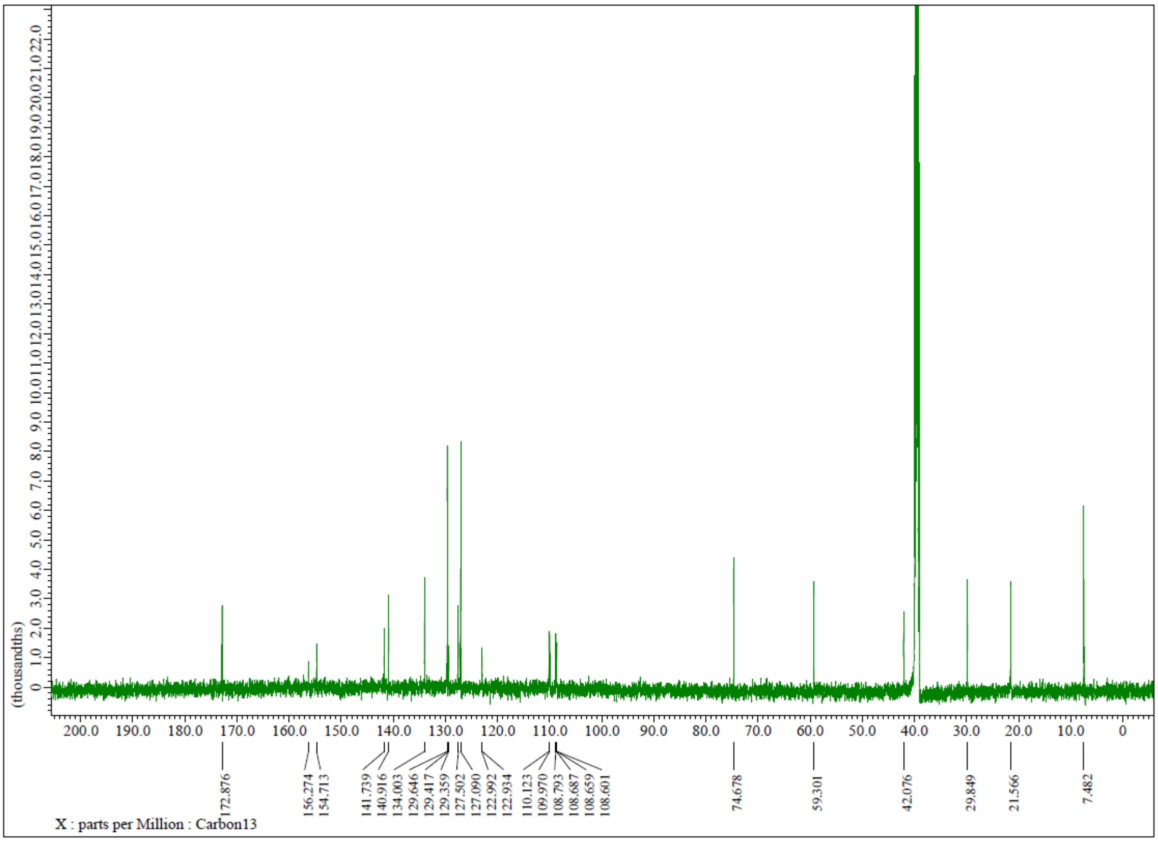

Compound **51**_ ^{1}H NMR (300 MHz, DMSO-d_6).

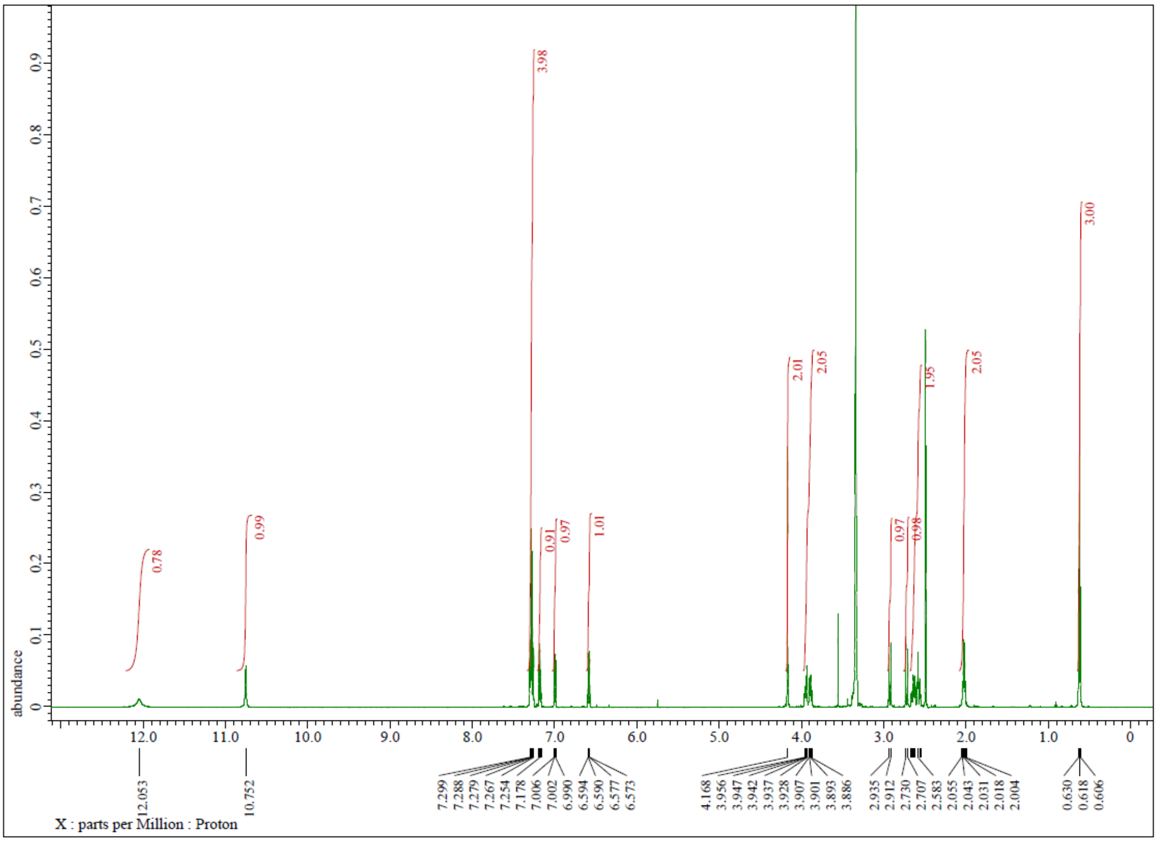

Compound **51**_ ^{13}C NMR (151 MHz, $CDCl_3$).

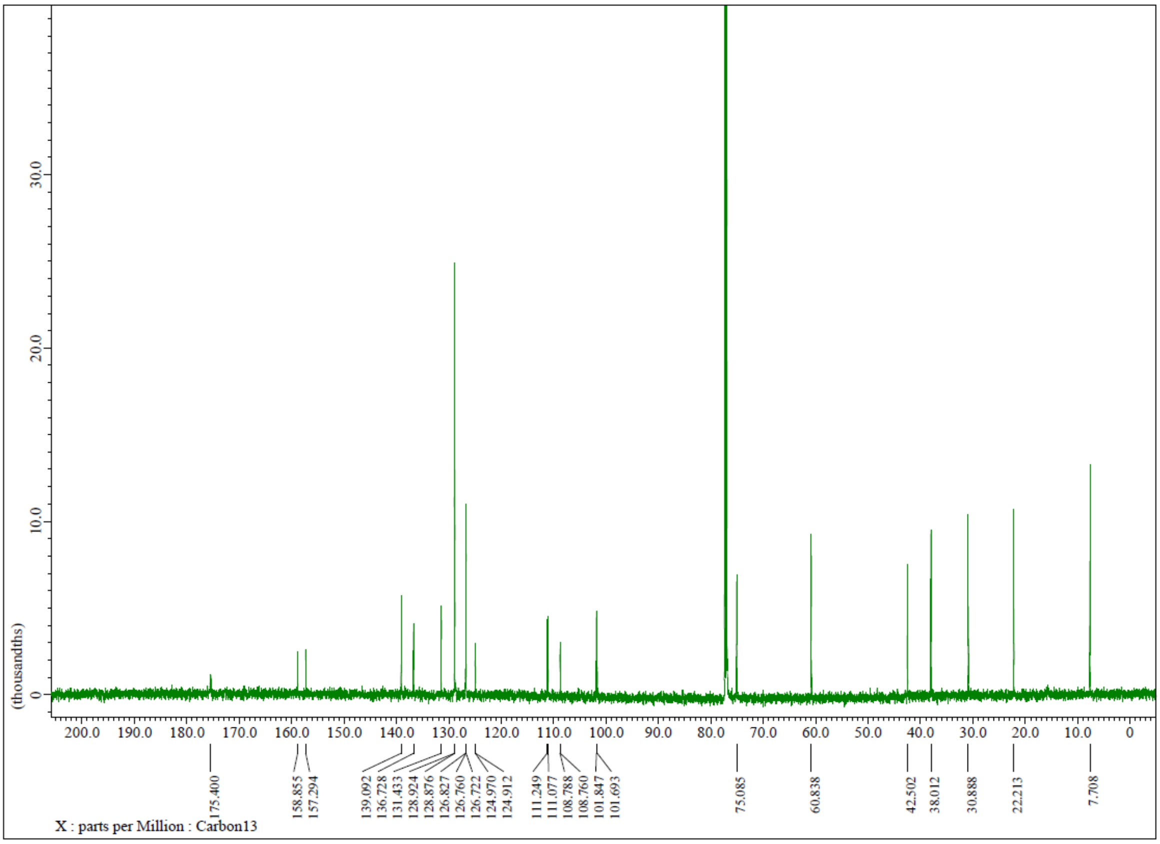

*HRMS spectra of compounds **21**, **25**, **32**, **48**, **50** and **51***

HRMS spectra were determined using a ZenoToF 7600 from Sciex (Framingham, MA) high-resolution mass spectrometer. Samples were infused at the concentration of 1 mg/L in methanol in the mass spectrometer operating in negative ESI mode with capillary voltage set to -4500 V and temperature set to 350 °C. Declustering potential was -80 ± 30 V and collision energy was 10 V. A full scan was performed from 100 to 1500 Da monitoring $[M-H]^-$ ions.

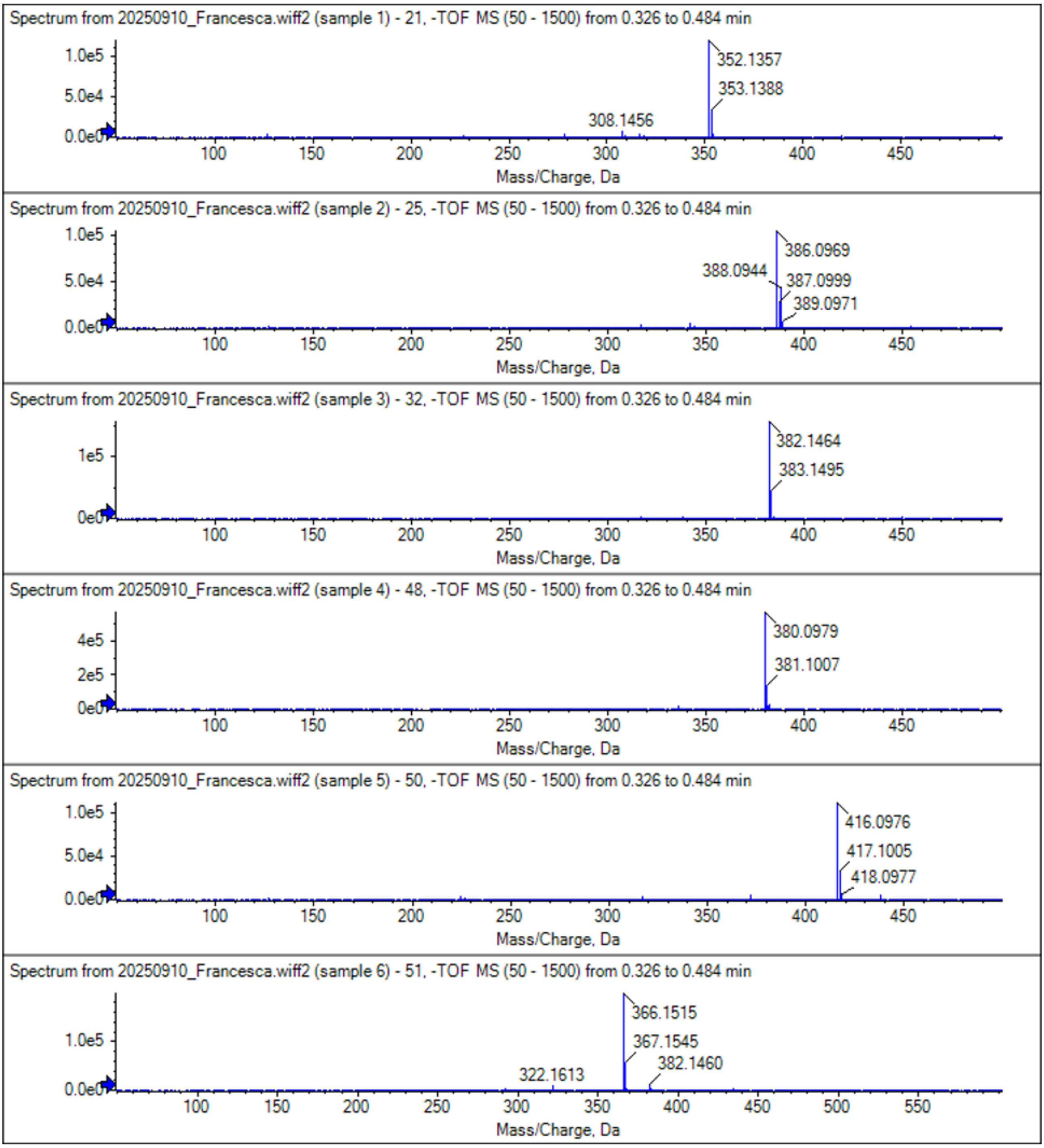

Supplement: Supplementary file 1 [file jm5c02068_si_001.pdf]
